# Does intrauterine crowding affect locomotor development? A comparative study of motor performance, neuromotor maturation and gait variability among piglets that differ in birth weight and vitality

**DOI:** 10.1371/journal.pone.0195961

**Published:** 2018-04-24

**Authors:** Charlotte Vanden Hole, Peter Aerts, Sara Prims, Miriam Ayuso, Steven Van Cruchten, Chris Van Ginneken

**Affiliations:** 1 Laboratory of Applied Veterinary Morphology, Department of Veterinary Sciences, Faculty of Biomedical, Pharmaceutical and Veterinary Sciences, University of Antwerp, Wilrijk, Belgium; 2 Laboratory of Functional Morphology, Department of Biology, Faculty of Sciences, University of Antwerp, Wilrijk, Belgium; 3 Department of Movement and Sports Sciences, Faculty of Medicine and Health Sciences, University of Ghent, Ghent, Belgium; INIA, SPAIN

## Abstract

In polytocous species, such as pigs, the growth of an individual fetus is affected by competition from its littermates and the sow. This intrauterine competition greatly influences postnatal traits such as birth weight and vitality (physical strength). A lowered vitality is most often observed among low birth weight piglets. Since it has been argued that locomotion might be key to unraveling vitality-related differences, we compared gait development in piglets with a low birth weight and low vitality (L piglets) with piglets with a normal birth weight and normal vitality (N piglets) by means of spatio-temporal gait analysis during locomotion at self-selected speed. Video recordings of L and N piglets walking along a corridor at ten time points (between birth and 96 h after birth) were made and the footfalls were digitized. Hence, self-selected speed, spatio-temporal characteristics and gait symmetry were analyzed to compare motor performance, neuromotor maturation (motor task, interlimb and intralimb coordination) and gait variability for L and N piglets. The analysis included both absolute and normalized data (according to the dynamic similarity concept), to distinguish neuromotor maturation from effects caused by growth. Results indicate that intrauterine crowding affects locomotion, mainly by impairing growth *in utero*, with a lowered motor performance during the first 96 h of age as a consequence. A difference in neuromotor skills was also visible, though only for swing and stance duration, implying a difference in neuromotor development *in utero*. However, further maturation during the first days after birth does not seem to be affected by intrauterine crowding. We can therefore conclude that L piglets might be considered a smaller and fictitious younger version of N piglets.

## Introduction

With regard to litter size, animals are generally subdivided into monotocous and polytocous species [1]. Monotocous animals produce, as a rule, one young at a time, though multiple births (twins, triplets, etc.) can occur. Humans, sheep, horses and cows are examples of monotocous animals. Polytocous animals, on the other hand, give birth to multiple young at a time. Mice, rats, rabbits and pigs belong to this category.

The study of polytocous species is particularly interesting because the growth of an individual fetus is affected by competition from its littermates and the mother. This competition can occur on a systemic (due to the total number of fetuses in the litter, growth of the mother), regional (due to the number of fetuses in a particular uterine horn) or local (due to the immediate proximity of neighboring fetuses) level [2]. The effect of uterine crowding on all these levels has been vastly studied in rats (e.g. [3, 4]), guinea pigs (e.g. [5]), mice (e.g. [2, 6]) and pigs (e.g. [7–11]).

In pigs, both uterine capacity [12–15] and placental function (size, vascularity and surface area; [16–18]) become the limiting factor for fetal survival and development after day 30 of gestation (total gestation takes 115 days). Père and Etienne [7] found that as litter size goes up in pigs, so does uterine blood flow, but not to an equivalent degree. Consequently, this (relatively) reduced blood flow leads to a reduced nutrient supply per fetus and hence an overall lighter body mass in large litters [7]. Moreover, from 30 days of gestation onwards, differences in placental weight start to occur in relation to uterine position. Given the competition among littermates for both nutrients and uterine space, this can lead to a heterogeneous body weight, especially in large litters, resulting in an increased presence of pigs with a low birth weight [8].

Needless to say, intrauterine competition greatly influences postnatal traits. In pigs, research has focused mainly on the effect on birth weight (e.g. [19]), body mass (e.g. [20–22], morphometric measurements (e.g. [7]) and sex ratios (e.g. [23, 24]). Previous studies also showed that uterine overcrowding has an effect on the muscle tissue, with low birth weight animals showing an altered muscle composition and reduced muscle fiber size [25]. Taken together, uterine overcrowding can lead to a lower birth weight, resulting in an impaired energy metabolism and a reduced physical performance [19, 26–29]. Piglets with reduced physical strength (or vigor) are referred to as low vitality piglets [30]. This lowered vitality is most often (though not exclusively) observed among low birth weight piglets [31]. Muns *et al*. [30] argue that the study of locomotion might be key to unraveling vitality-related differences. However, what these differences in locomotion exactly entail and whether these differences between low birth weight piglets with a low vitality (L piglets) and piglets with a normal birth weight and normal vitality (N piglets) are solely a question of the degree of morphological maturation (e.g. muscles) or energy levels, and/or also a question of differences in neuromotor control, has not been investigated up to this day.

As argued in our previous study [32], spatio-temporal characteristics can be considered the most basic collective output emerging from the entire underlying neuro-mechanical system (musculo-skeletal system and its control), with relative phasing of the limbs revealing aspects of interlimb coordination and single limb behavior (during stance and swing) as a collective measure of intralimb coordination. However, in order to attribute changes in spatio-temporal gait characteristics to neuromotor maturation, one needs to be able to distinguish between maturation on the one hand and growth or postural changes on the other hand. To this end, normalization according to the dynamic similarity principle [33] is applied (cf. [32]). If the dynamics of the motor task do not change during development, we assume no underlying neuromotor maturation. In this case, differences in spatio-temporal gait characteristics are considered to be an effect of growth. If there are, however, changes in normalized spatio-temporal gait characteristics over age, this would imply neuromotor maturation on top of growth effects.

Our earlier study [32] showed that N piglets undergo a very rapid (within a few hours after birth) neuromotor maturation, with a rapidly decreasing variability of the gait pattern (within 8 hours after birth). We expect that L piglets show a lower level of neuromotor skill at birth and afterwards show a differential/slower neuromotor maturation, compared to N piglets. To this end, we compare spatio-temporal gait characteristics (both absolute and normalized) and gait variability of L piglets to that of N piglets.

More specifically, we addressed the following questions:

- Is motor performance different for L and N piglets at birth and during early development? We hypothesize a reduced motor performance for L piglets, compared to N piglets, both at birth as during early development. To this end we check for differences in (absolute values) of *self-selected speed* (*u*), *stride frequency* (*f*) and *stride length* (*l*_stride_).
- Are neuromotor skills different at birth and is the associated neuromotor maturation different for L and N piglets? This question entails three hypotheses, which can be answered by studying different aspects of spatio-temporal coordination.
  - Is voluntary locomotion for L piglets at *u* dynamically similar to N piglets after a few hours [32] and is, in that case, the locomotion of both groups identical? We hypothesize that normalized walking at birth is different for L piglets compared to N piglets and that this is followed by a slower maturation. For this purpose we will normalize *u* and its components (*f* and *l*_stride_) according to the dynamic similarity principle, generating *u’*, *f’*, *l*_stride_*’* (cf. [32, 33]). We checked for differences in the development of *u’*, *f’* and *l*_stride_*’* between L and N piglets.
  - Is interlimb coordination at birth and its postnatal development different for L and N piglets? In N piglets interlimb coordination is shown to be innate [32]. Therefore we hypothesize a similar postnatal development for both groups, although the level of interlimb coordination at birth might be different due to unfavorable intrauterine conditions in case of L piglets. For this purpose we checked for differences in development of relative phasing of the limbs between N and L piglets. If the relative timings of the footfalls (i.e. the front lag (f-lag), hind lag (h-lag), pair lags (p-lag) and diagonal lags (d-lag), see [34]) are the same for L and N piglets, the hypothesis is confirmed.
  - Is intralimb coordination (individual limb behavior) at birth and its postnatal development different for L piglets, compared to N piglets? We hypothesize this is the case. To this end we compared the development with age of normalized swing (*t*_sw_*’*) and stance duration (*t*_st_*’*), duty factor (df), normalized step length (*l*_step_*’*) and normalized maximum swing height (*h*_swmax_*’*) between L and N piglets.
- Is the variability of the gait pattern different for L and N piglets at birth? Do L piglets take a longer time to achieve a stable gait pattern than N piglets? We hypothesize a higher variability of the gait pattern at birth for L piglets, compared to N piglets. In addition we hypothesize L piglets will take a longer time to achieve a stable gait pattern. To this end we compared left-right asymmetries for L and N piglets between developmental stages.
- If, contradictory to our abovementioned expectations, neuromotor maturation does appear to be similar for L and N piglets, this would imply that observed absolute differences in spatio-temporal gait characteristics are mainly a consequence of growth. In this case it is worth investigating whether L piglets can actually be considered a smaller version of N piglets. To this end we compare *u*, *l*_stride_, *l*_step_ and *f* in function of body mass instead of age.

## Materials and methods

### Selection of piglets

Institutional and national guidelines for the care and use of animals were followed and all experimental procedures involving animals were approved by the Ethical Committee of Animal Experimentation, University of Antwerp, Belgium (approval number 2015–26). Twenty-five domestic piglets (*Sus scrofa*, Topigs x Belgian Piètrain) from 11 litters were selected between February and May 2015 on a local farm. The average number of piglets born alive within these litters was 16.5 (± 3.8) (mean ± SD, here and throughout). Between 1 and 5 healthy piglets per litter were selected immediately post-partum and ear notched upon selection. Sex was not used as a criterion. In total 16 females and 9 males were selected, for more details on the selected piglets, see Table 1. Piglets were chosen, based on their body mass at birth and vitality score [32]. Each piglet was weighed at birth and its vitality was scored based on respiration (0–2, no to regular respiration) and movement (0–2, no movement to taking a few steps). Animals that scored 0 out of 4 were dead, a score 1 or 2 out of 4 was considered indicative of a low vitality, while animals that scored 3 or 4 were considered to have a normal vitality. Piglets with a body mass at birth that was within the limits of the average birth weight in the litter ± 1 SD and with a normal vitality were classified as N piglets (n = 14). These N piglets were the same piglets as used in Vanden Hole *et al*. [32]. Piglets with a birth weight smaller than the average body mass at birth—1 SD and with a low vitality were classified as L piglets (n = 11). This method of classifying L and N piglets was chosen because of large between-litter variability in body mass at birth [21]. Animals with a normal birth weight and low vitality or with a low birth weight and normal vitality were excluded. The average birth weight for the L category was 0.79 kg (± 0.17) and 1.19 kg (± 0.22) for N piglets. Every piglet was weighed before each walking session.

**Table 1:** Selected piglets per sow, including category (N or L piglet) and sex.

Sow	N piglets		L piglets		Total number of piglets per sow
	Male	Female	Male	Female	
1	-	1	-	-	1
2	2	-	-	1	3
3	-	-	-	1	1
4	-	-	1	-	1
5	1	1	1	2	5
6	1	2	-	1	4
7	1	-	-	-	1
8	2	-	-	-	2
9	-	-	-	2	2
10	-	2	-	2	4
11	-	1	-	-	1

### Video sequences

Lateral video recordings were made from animals walking through a custom made corridor (provided with a reference grid for scaling purposes, for more details see Vanden Hole, Goyens (32)), perpendicular to the camera (3.3 megapixel, 50 Hz deinterlaced, JVC GZ-V515, JVC Kenwood Corporation, Kanagawa, Japan).

Animals were recorded at ten points in time (0, 1, 2, 4, 6, 8, 24, 26, 28 and 96 h after birth). The close spacing of these time points allowed for a detailed description and comparison of motor performance, neuromotor maturation and variability of the gait pattern during early development. As explained in Vanden Hole *et al*. [32] age 96 h was chosen as a control age (further referred to as CA 96 h), to which all other ages were compared. For each time point 2 video recordings were analyzed, which would have led to 500 analyzed trials. However, 4 animals (all L) died between the age of 24 h and CA 96 h and an additional 36 trials were discarded due to the lack of a complete stride in the recorded sequence. Of these 36 discarded trials, 24 belonged to L piglets, and 12 to N piglets. Most (20) discarded trials were recorded at age 0 h. The final number of trials that were included in the analysis was 440.

### Gait analysis

All video sequences were digitized field-by-field using Matlab (Mathworks, Natick, Massachusetts, USA), following the method described in Vanden Hole *et al*. [32]. This method entails the digitization of 5 body points: the most distal point of the distal phalanx of each leg and either the eye or the ear notch (used as a proxy for the overall displacement of the body throughout a stride, for more details see Vanden Hole *et al*. [32]). For the normalization procedure, it was also necessary to digitize a locomotion-related linear dimension. We opted to use the functional hind limb length/height (*H*), i.e. the distance between the most distal part of the distal phalanx and a fixed point on the pelvis, i.e. the tail base. *H* was determined for each sequence, in the frame coinciding with midstance of the hind limb closest to the camera.

One extra variable, the clearance of the limb during swing (*h*_swmax_) was added to the 19 gait-variables included by Vanden Hole *et al*. [32], see Table 1. For *u*, *f*, *l*_stride_ and *l*_step_, both absolute and normalized (according to the dynamic similarity principle) variables were studied. For all other spatio-temporal gait variables only the normalized (according to the dynamic similarity principle) variables were studied. The dataset of the N piglets is the same as the one used in Vanden Hole *et al*. [32], though in our current study the data were (statistically) analyzed anew, together with the L piglet data. Table 2 includes all used variables, abbreviations, definitions and normalization formulas. All calculated variables, both normalized and not normalized, can be found in S1–S7 Tables.

**Table 2:** Summary of all used variables (abbreviations, definitions and formulas, including normalization procedure; adapted from Vanden Hole *et al*.[32]. If normalized, variables are indicated with ‘ in the text.

Variable	Abbreviation	Definition	Formula	Normalization
Gravitational acceleration	*g*	NA	NA	NA
Self-selected speed	*u*	The movement of the center of mass (COM) during one cycle divided by the duration of the cycle. Animals are able to move in an unrestrained, voluntary way.	*fl*_stride_	$\frac{u}{\sqrt{H\;g}}$
Stride frequency	*f*	Inverse of the period between two consecutive footfalls of a certain leg.	$\frac{u}{l_{stride}}$	$f\;\sqrt{H\;g}$
Stride length	*l*_stride_	The forward movement during one stride or cycle.	$\frac{u}{f}$	$\frac{l_{stride}}{H}$
Stance duration	*t*_*st*_	The period of contact between a limb and the ground.	NA	$\frac{t_{st}}{\sqrt{H/g}}$
Swing duration	*t*_sw_	The period of limb flight.	NA	$\frac{t_{sw}}{\sqrt{H/g}}$
Step length	*l*_step_	The movement of the COM during one step (stance phase only).	NA	$\frac{l_{step}}{H}$
Duty factor	*df*	The fraction of the cycle for which the limb is in contact with the ground.	NA	NA
Maximum swing height	*h*_swmax_	The maximum amount the leg is lifted from the ground during the swing phase.	NA	$\frac{h_{swmax}}{H}$
Front lag	f-lag	The time lag between the two front footfalls in function of the average cycle duration of the front leg pair (d_front_). Adapted from Abourachid [34].	$\frac{f-lag}{d_{front}}$	NA
Hind lag	h-lag	The time lag between the two hind footfalls in function of the average cycle duration of the hind leg pair (d_hind_). Adapted from Abourachid [34].	$\frac{h-lag}{d_{hind}}$	NA
Pair lag	p-lag	The time lag between the two ipsilateral footfalls in function of the average cycle duration of the ipsilateral leg pair (d_ipsi_). Adapted from Abourachid [34].	$\frac{p-lag}{d_{ipsi}}$	NA
Diagonal lag	d-lag	The time lag between the two diagonal footfalls in function of the average cycle duration of the diagonal leg pair (d_dia_). Adapted from Abourachid [34].	$\frac{d-lag}{d_{dia}}$	NA
AI stride frequency	AI_*f*_	Asymmetry index of the stride frequency. Adapted from Robinson *et al*. [35].	$\frac{\left(f_{R}-f_{L}\right)}{0.5\;(f_{R}+f_{L})}\;100\%$	NA
AI stride length	AI_*l*stride_	Asymmetry index of the stride length. Adapted from Robinson *et al*. [35].	$\frac{\left(l_{stride,R}-l_{stride,L}\right)}{0.5\;(l_{stride,R}+l_{stride,L})}\;100\%$	NA
AI stance duration	AI_*t*st_	Asymmetry index of the stance duration. Adapted from Robinson *et al*. [35].	$\frac{\left(t_{st,R}-t_{st,L}\right)}{0.5\;(t_{st,R}+t_{st,L})}\;100\%$	NA
AI swing duration	AI_*t*sw_	Asymmetry index of the swing duration. Adapted from Robinson *et al*. [35].	$\frac{\left(t_{sw,R}-t_{sw,L}\right)}{0.5\;(t_{sw,R}+t_{sw,L})}\;100\%$	NA
AI step length	AI_*t*sl_	Asymmetry index of the step length. Adapted from Robinson *et al*. [35].	$\frac{\left(l_{step,R}-l_{step,L}\right)}{0.5\;(l_{step,R}+l_{step,L})}\;100\%$	NA
AI duty factor	AI_df_	Asymmetry index of the duty factor. Adapted from Robinson *et al*. [35].	$\frac{\left({df}_{R}-{df}_{L}\right)}{0.5\;({df}_{R}+{df}_{L})}\;100\%$	NA

### Statistics

To evaluate the effect of age, category (L or N) and leg on the outcome variables, linear mixed models were fitted. Fixed factors included age, category and leg, though the latter was only included if it was relevant for the parameter in question. To avoid the model becoming too complex and because it was not the focus of this study, sex was not included as a covariate. In addition, our previous study showed it to have no significant effect on any of the investigated variables in N piglets ([32], section Statistics). Due to the experimental design, we had to account for the dependence between observations within each litter and within the same animal (10 time points). For this purpose, random factors were included for sow and piglet (nested in sow), plus random slopes for age, leg and piglet (nested in sow). This starting model was gradually simplified, using stepwise backwards modelling.

To evaluate the effect of category on *u*, *l*_stride_, *l*_step_ and *f* across all age categories and whether it covaried with body mass, linear mixed models were also used. Category was added as a fixed factor, while body mass (and the interaction with category) was included as a covariate. Age was included as a repeated measure. To account for the dependence between observations between littermates and within the same animal, random factors were included for sow and piglet (nested in sow), plus random slopes for leg and piglet (nested in sow). Stepwise backwards modelling was again used to simplify the starting model.

To meet normality and/or homoscedasticity assumptions, some outcome variables required transformations. *f’*, *t*_st_*’*, AI_*f*_, AI_*l*stride_, AI_*l*step_ and AI_df_ were log transformed, while *f*, *l*_stride_, *u’*, *l*_stride_*’*, *l*_step_*’*, *t*_sw_*’*, *h*_swmax_*’*, AI_*t*st_ and AI_*t*sw_ were square root transformed. *u*, *l*_step_ and df required no transformations.

JMP^®^ Pro 12 (SAS Institute Inc., USA) was used for the entire analysis. Values were considered statistically significant if *p* ≤ 0.05. *Post hoc* analysis with Dunnett’s correction was used to compare the different age groups to CA 96 h, while *post hoc* analysis with Tukey’s correction was applied for the comparison of different legs.

## Results

### Morphometrics

**Fig 1:**
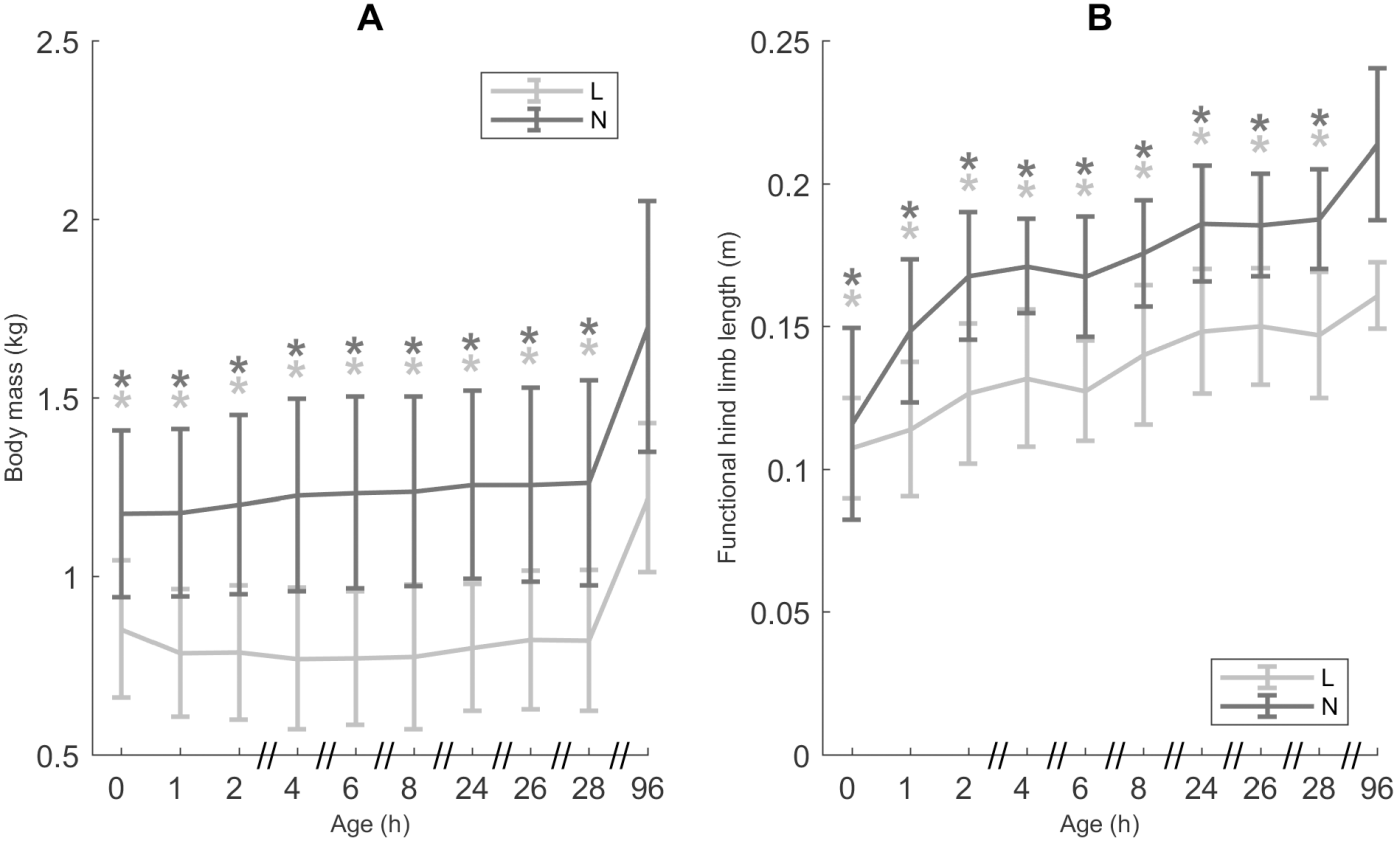
Morphometrics. A. Body mass (n = 25, data points = 226). B. Functional hind limb length (*H*, n = 25, data points = 226). All values are mean ± SD. Mean values indicated with * differ significantly from CA 96 h (linear mixed models, *post hoc* analysis with Dunnett’s correction, *p* ≤ 0.05). Mean values for body mass and *H* differ significantly between low birth weight/low vitality (L) and normal birth weight/normal vitality (N) piglets (linear mixed models, *p* ≤ 0.05).

The change in body mass and *H* with age are shown in Fig 1. Overall body mass was lower for L piglets than for N piglets. During the first 28 h body mass changes were negligible for both N (+ 0.46%) and L piglets (- 0.96%). However, in both L and N piglets body mass increased from age 28 h to CA 96 h (+ 48.78% for L piglets; + 34.92% for N piglets; *p* < 0.0001). The lack of a significant interaction effect between age and category indicates that the growth pattern was not significantly different for L and N piglets.

Similar to body mass, *H* was shorter for L piglets than for N piglets. A fairly gradual increase in *H* between the age of 0 h and 96 h was visible for both groups (L: 0.11 m (± 0.02) to 0.16 m (± 0.01); N: 0.12 m (± 0.03) to 0.21 m (± 0.03); *p* < 0.0001). Since body mass remained constant during the first 28 h, the increase of *H* after 28 h could be attributed to growth, while the increase in *H* up to the age of 28 h could be attributed to acquiring a more erect posture. As mentioned in Vanden Hole *et al*. [32] the largest increase during the first 28 h in N pigs seemed to occur in the first hours after birth. For L piglets it seemed that the increase in *H* via attaining a more erect limb posture took longer, i.e. the entire period between age 0 h and 28 h.

### Motor performance

**Fig 2:**
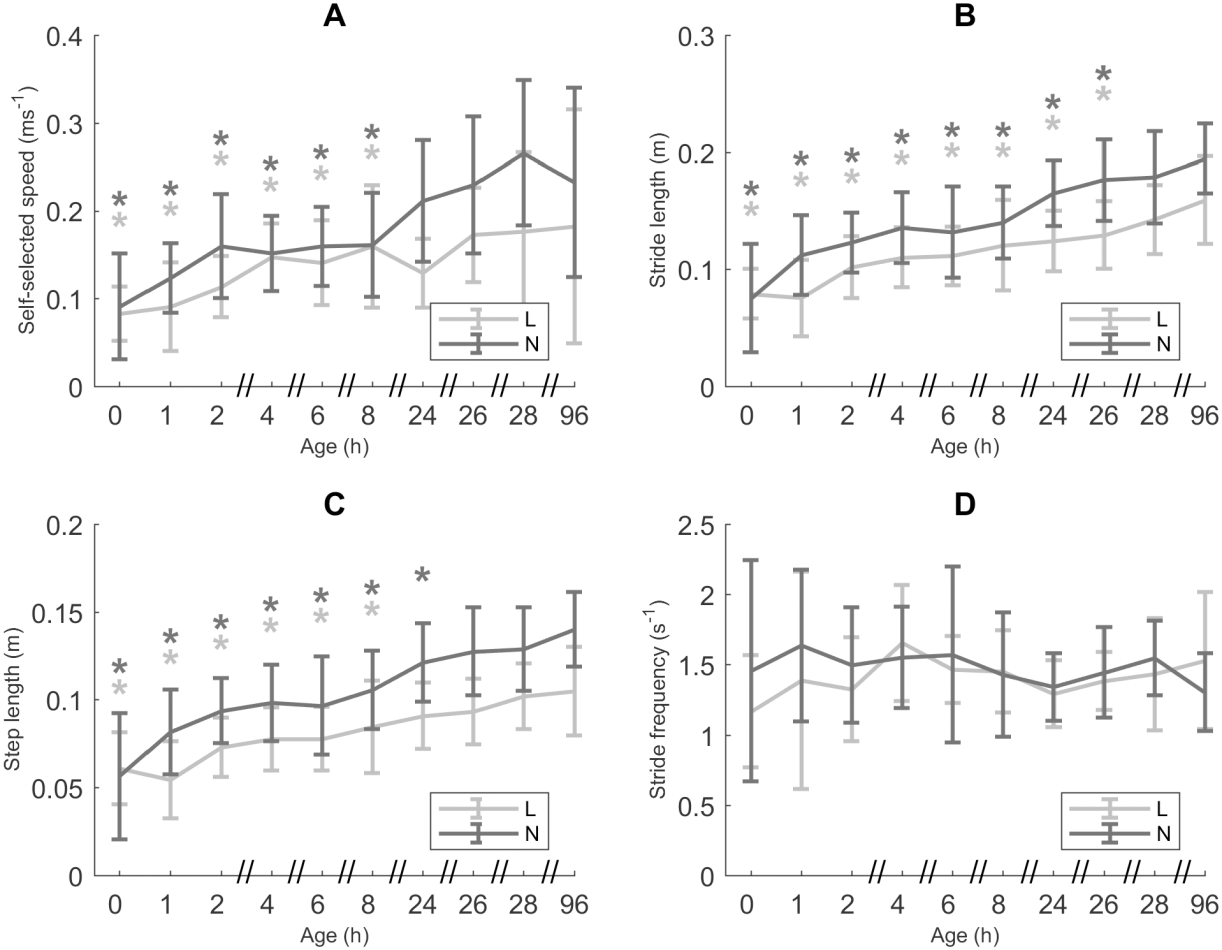
Motor performance. A. Self-selected speed (*u*, n = 25, data points = 226). B. Stride length (*l*_stride_, n = 25, data points = 904). C. Step length (*l*_step_, n = 25, data points = 904). D. Stride frequency (*f*, n = 25, data points = 904). All values are mean ± SD. Mean values indicated with * differ significantly from CA 96 h (linear mixed models, *post hoc* analysis with Dunnett’s correction, *p* ≤ 0.05). Mean values for *u*, *l*_stride_ and *l*_step_ differ significantly between low birth weight/low vitality (L) and normal birth weight/normal vitality (N) piglets (linear mixed models, *p* ≤ 0.05).

A significantly lower *u* was found at ages 0–8 h, compared to CA 96 h (*p* < 0.0001 (0 h and 1 h), 0.0013 (2 h), 0.0072 (4 h), 0.0095 (6 h) and 0.0380 (8 h); Fig 2A). For L piglets a significantly lower mean *u* was found (0.14 ms^-1^ (± 0.07)), than for N piglets (0.17 ms^-1^ (± 0.08), *p* = 0.0122). Similarly, *l*_stride_ was shorter for L piglets, compared to N piglets (0.11 m (± 0.04) vs 0.14 m (± 0.05), *p* = 0.0021; Fig 2B). Compared to CA 96 h, *l*_stride_ was shorter from age 0 h up to and including 26 h (*p* < 0.0001 (0 h to 8 h), *p* = 0.0019 (24 h) and *p* = 0.0435 (26 h)). L piglets generally showed a shorter *l*_step_ compared to N piglets (*p* = 0.0282; Fig 2C). In addition, in L piglets *l*_step_ seemed to increase up to and including 8 h (*p* < 0.0001 (0 h and 1 h), *p* = 0.0004, 0.0036, 0.0040 and 0.0422 for 2, 4, 6 and 8 h, respectively), while in N piglets *l*_step_ continued to increase for a longer time (up to and including 24 h, *p* < 0.0001 and *p* = 0.0210 (24 h)). For L piglets we saw an increase in *l*_step_ from 0.06 m (± 0.02) to 0.08 m (± 0.03) between the age 0 h and 8h (*p* < 0.0001 (0 h and 1 h), *p* = 0.0004 (2 h), *p* = 0.0036 (4 h), *p* = 0.0040 (6 h) and *p* = 0.0422 (8 h)), while N piglets showed an increase from 0.06 m (± 0.04) to 0.12 (± 0.02) between 0 h and 24 h (*p* < 0.0001 (0 h– 8 h) and *p* = 0.0210 (24 h). In contrast, no significant effects were found for *f* (Fig 2D).

### Neuromotor control

#### Motor task

**Fig 3:**
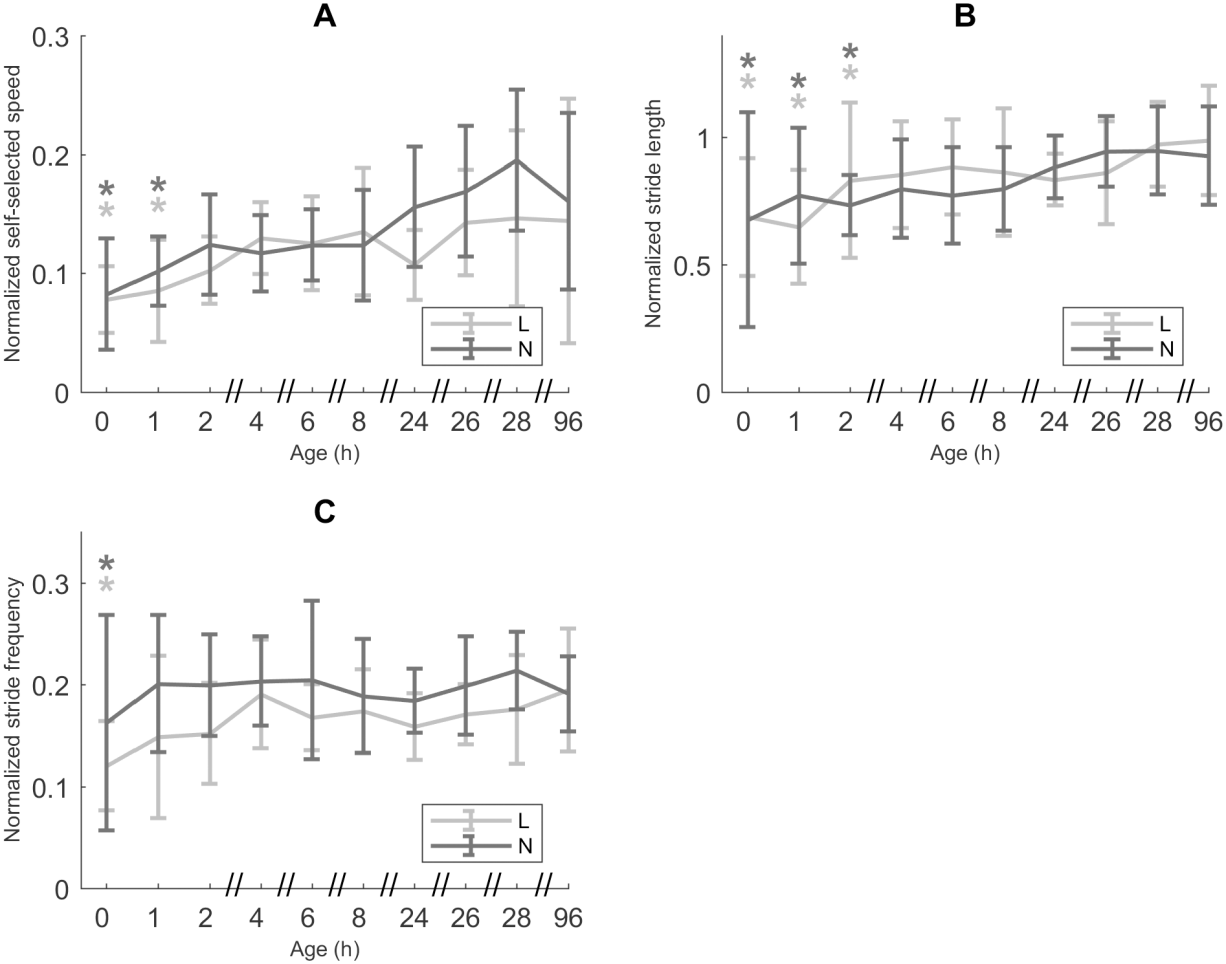
Motor task. A. Normalized self-selected speed (*u’*, n = 25, data points = 226). B. Normalized stride length (*l*_stride_’, n = 25, data points = 904) C. Normalized stride frequency (*f’*, n = 25, data points = 904). All values are mean ± SD. Mean values indicated with * differ significantly from CA 96 h (linear mixed models, *post hoc* analysis with Dunnett’s correction, *p* ≤ 0.05). Mean values for *f* differ significantly between low birth weight/low vitality (L) and normal birth weight/normal vitality (N) piglets (linear mixed models, *p* ≤ 0.05).

The statistical analysis revealed *u’* did not differ significantly between L and N piglets (Fig 3A). For both categories, *u’* at ages 0 h and 1 h was significantly lower than *u’* at CA 96 h, comprising respectively only 52.44% and 61.62% of the *u’* at CA 96 h(*p* < 0.0001 and 0.0008, respectively). Similarly, *l*_stride_*’* was not significantly different for L and N piglets (Fig 3B). *l*_stride_*’* was significantly lower at ages 0 to 2 h than at CA 96 h (*p* < 0.0001 (0 h and 1 h) and 0.0026 (2 h)). *l*_stride_*’* at ages 0 h, 1 h and 2 h made up 71.59%, 76.21% and 81.40% of *l*_stride_*’* at CA 96 h, respectively. However, for *f’* we did find a lower mean value for L piglets (14.13% lower than for N piglets, *p* = 0.0131; Fig 3C). *f’* stabilized within the hour for all four legs, evidenced by the fact that for the left front (LF), right front (RF) and right hind (RH) leg the *f’* was only significantly different from the *f’* at CA 96 h at age 0 h (*p* < 0.0001 (LF, RF) and *p* = 0.0010 (RH)).

#### Relative limb-phasing

The time lags did not differ significantly between categories and ages. Values for f-lag, h-lag, p-lag (left), p-lag (right), d-lag (LH-RF), d-lag (RH-LF) were 0.51 (± 0.15), 0.49 (± 0.15), 0.68 (± 0,17), 0.34 (± 0.16); 0.26 (± 0.19) and 0.20 (± 0.11), respectively.

#### Limb-specific coordination

**Fig 4:**
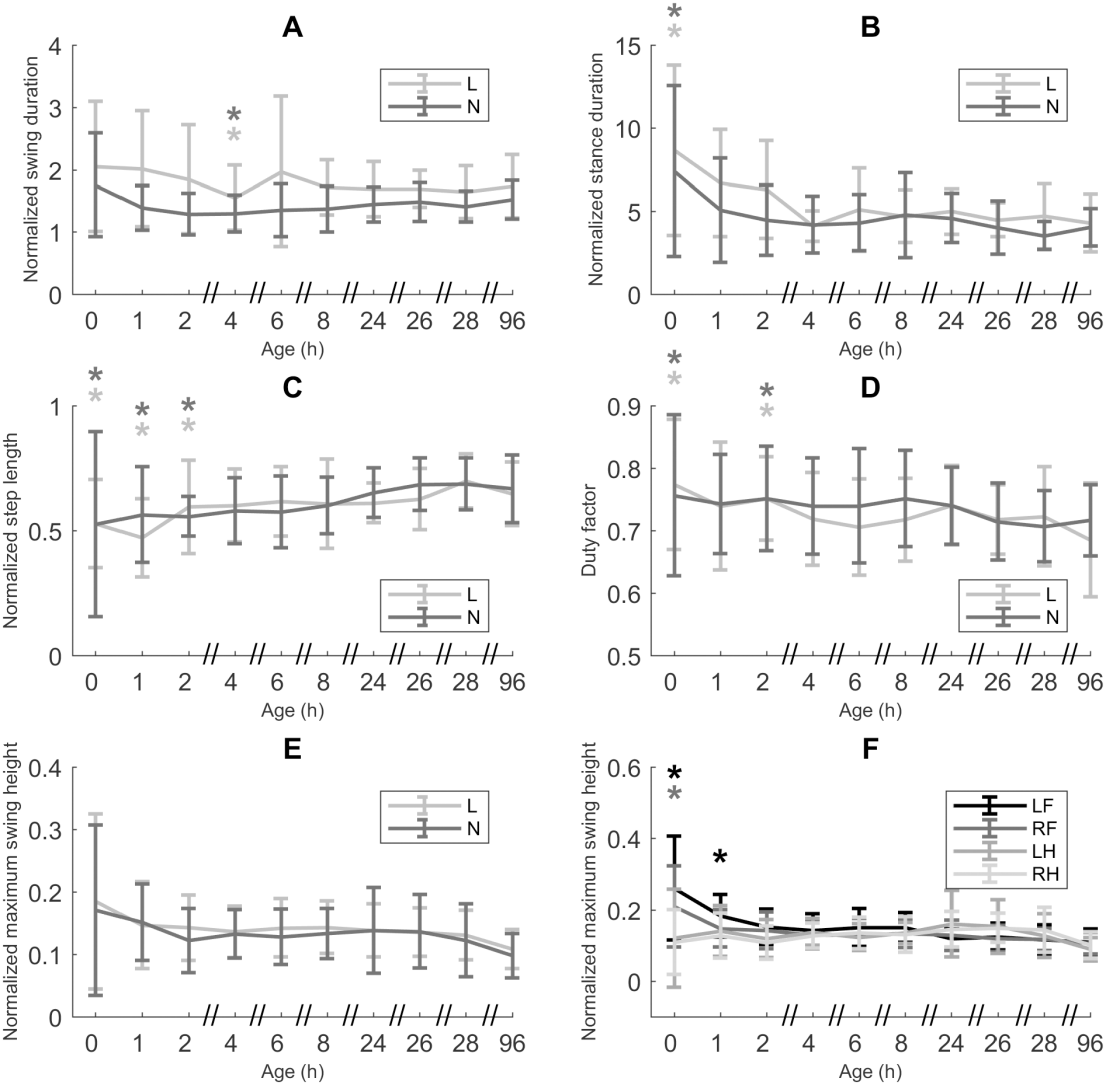
Limb-specific coordination. A. Normalized swing duration (*t*_*sw*_*’*, n = 25, data points = 904). B. Normalized stance duration (*t*_*st*_*’*, n = 25, data points = 904). C. Normalized step length (*l*_step_’, n = 25, data points = 904). D. Duty factor (df, n = 25, data points = 904). E. Normalized maximum swing height (*h*_swmax_*’*, n = 25, data points = 904). F. Normalized maximum swing height (*h*_swmax_*’*, n = 25, data points = 904) per leg (left front (LF), right front (RF), left hind (LH), right hind (RH)). All values are mean ± SD. Mean values indicated with * differ significantly from CA 96h (linear mixed models, *post hoc* analysis with Dunnett’s correction, *p* ≤ 0.05). Mean values for *t*_*sw*_*’* and *t*_*st*_*’* differ significantly between low birth weight/low vitality (L) and normal birth weight/normal vitality (N) piglets (linear mixed models, *p* ≤ 0.05).

*t*_sw_*’* was 24.65% longer for L piglets than for N piglets (*p* = 0.0010; Fig 4A). At the age of 4h, *t*_sw_*’* was significantly shorter than the *t*_sw_*’* at CA 96 h, for both L and N piglets (*p* = 0.0046). For *t*_st_*’* we also saw an overall difference between L and N piglets (Fig 4B). The *t*_st_*’* of L piglets was 13.31% longer than that of N piglets (*p* = 0.0392). Compared to CA 96 h, only age 0 h showed a significantly longer *t*_st_*’* (87.91% longer, *p* < 0.0001), indicating that *t*_st_*’* reached a stable value already within the first hour after birth. No significant differences between L and N piglets were found for *l*_step_*’*. *l*_step_*’* reached a stable value between the age of 2 h and 4 h, since smaller mean values were observed between ages 0–2 h (*p* < 0.0001, *p* = 0.0002 and 0.0370, respectively; Fig 4C). For these early ages, *l*_step_*’* was 79.63%, 79.82% and 86.59% of the *l*_step_*’* at CA 96 h. The df was not significantly different for L and N piglets. A higher df was observed for ages 0 h and 2 h (*p* = 0.0028 and 0.0120, respectively; Fig 4D), compared to CA 96 h. *h*_swmax_*’* for both L and N piglets hind legs showed no significant effect of age, while front legs did (Fig 4E and 4F). In other words, the *h*_swmax_*’* of the hind legs did not show any change with age, while the *h*_swmax_*’* of the front legs did. For LF we saw a higher *h*_swmax_*’* at ages 0 h and 1 h, compared to CA 96 h (287.38% and 160.28% of CA 96 h, *p* < 0.0001 and *p* = 0.0289 for L piglets; 217.58% and 169.25% of CA 96 h, *p* = 0.0001 and 0.0079 for N piglets). For RF the *h*_swmax_*’* was only different for age 0 h, being 215.79% of the *h*_swmax_*’* at CA 96 h for L piglets (*p* = 0.0002) and 188.99% of CA 96 h for N piglets (*p* < 0.0001).

### Gait stability

**Fig 5:**
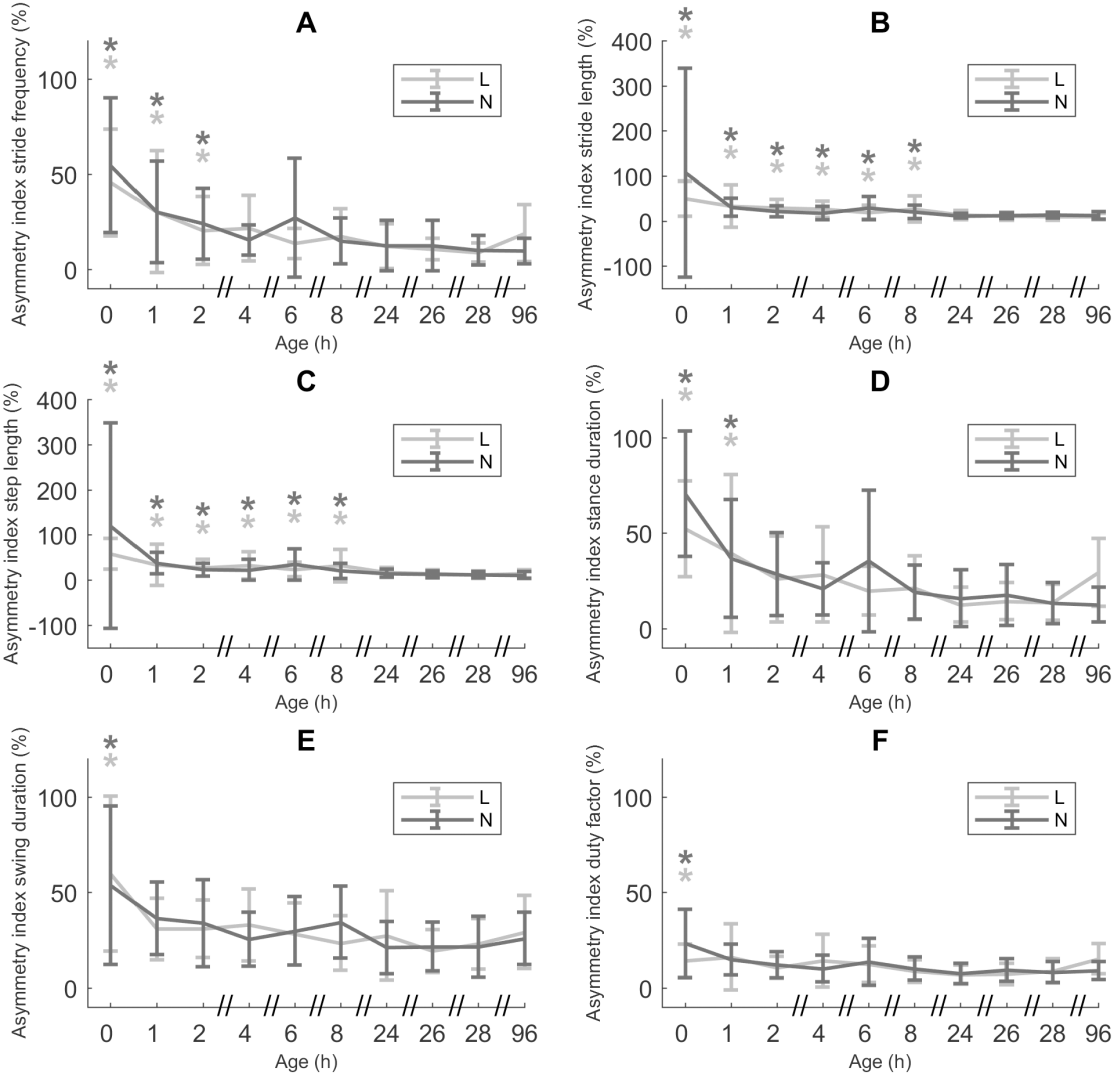
Gait stability. A. Asymmetry index stride frequency (AI_*f*_, n = 25, data points = 452). B. Asymmetry index stride length (AI_*l*stride_, n = 25, data points = 452). C. Asymmetry index step length (AI_*l*step_, n = 25, data points = 452). D. Asymmetry index stance duration (AI_*t*st_, n = 25, data points = 452). E. Asymmetry index swing duration (AI_*t*sw_, n = 25, data points = 452). F. Asymmetry index duty factor (AI_df_, n = 25, data points = 452). All values are mean ± SD. Mean values indicated with * differ significantly from CA 96h (linear mixed models, *post hoc* analysis with Dunnett’s correction, *p* ≤ 0.05). No mean values differ significantly between low birth weight/low vitality (L) and normal birth weight/normal vitality (N) piglets (linear mixed models).

None of the asymmetry indices were significantly different between L and N piglets (Fig 5). AI_*f*_ showed a higher value at ages 0 h, 1 h and 2 h, compared to CA 96 h (*p* < 0.0001, p = 0.0016 and 0.0167, respectively; Fig 5A). Differences were very substantial, with mean values for ages 0 h, 1 h and 2 h being 52.19% (± 33.30), 30.19% (± 28.46) and 22.61% (± 18.18). At CA 96 h AI_*f*_ had diminished to 13.01% (± 11.16). AI_*l*stride_ was significantly higher for ages 0 h up to and including 8 h, compared to CA 96 h (*p* < 0.0001 for ages 0 h and 1 h, *p* = 0.0002 for age 2 h, 0.0156 for age 4 h, 0.0050 for age 6 h and 0.0305 for age 8 h; Fig 5B). Values for AI_*l*stride_ at ages 0, 1, 2, 4, 6 and 8 h were 91.05% (± 198.92), 31.32% (± 32.83), 24.11% (± 15.91), 21,27% (± 16.80), 24.40% (± 21.90) and 22.48% (± 22.05), while at CA 96 h AI_*l*stride_ decreased to 11.56% (± 7.71). Similar results were found for AI_*l*step_, with AI_*l*step_ for ages 0, 1, 2, 4, 6 and 8 h showing a significantly higher value than at CA 96 h (*p* < 0.0001 (ages 0 h and 1 h), *p* = 0.0010, 0.0040, 0.0006 and 0.0118, respectively; Fig 5C). Mean values were 103.09% (± 195.32), 35.66% (± 33.41), 24.63% (± 15.97), 26.71% (± 26.64), 29.22% (± 27.80) and 25.64% (± 26.80), respectively, while the mean value at CA 96 h was 12.06% (± 8.54). AI_*t*st_ was higher at ages 0 h (65.50% (± 31.61)) and 1 h (37.72% (± 34.85)), compared to an AI_*t*st_ of 18.42% (± 15.04) at CA 96 h (p < 0.0001 and *p* = 0.0035; Fig 5D). AI_*t*sw_ at age 0 h was significantly higher (55.50% (± 40.74)) than at CA 96 h (27.13% (± 15.64), *p* < 0.0001; Fig 5E). Similarly, AI_df_ was also significantly higher at age 0 h (20.82% (± 16.29)), compared to CA 96 h (11.29% (± 6.65)) (*p* = 0.026; Fig 5F).

**Fig 6:**
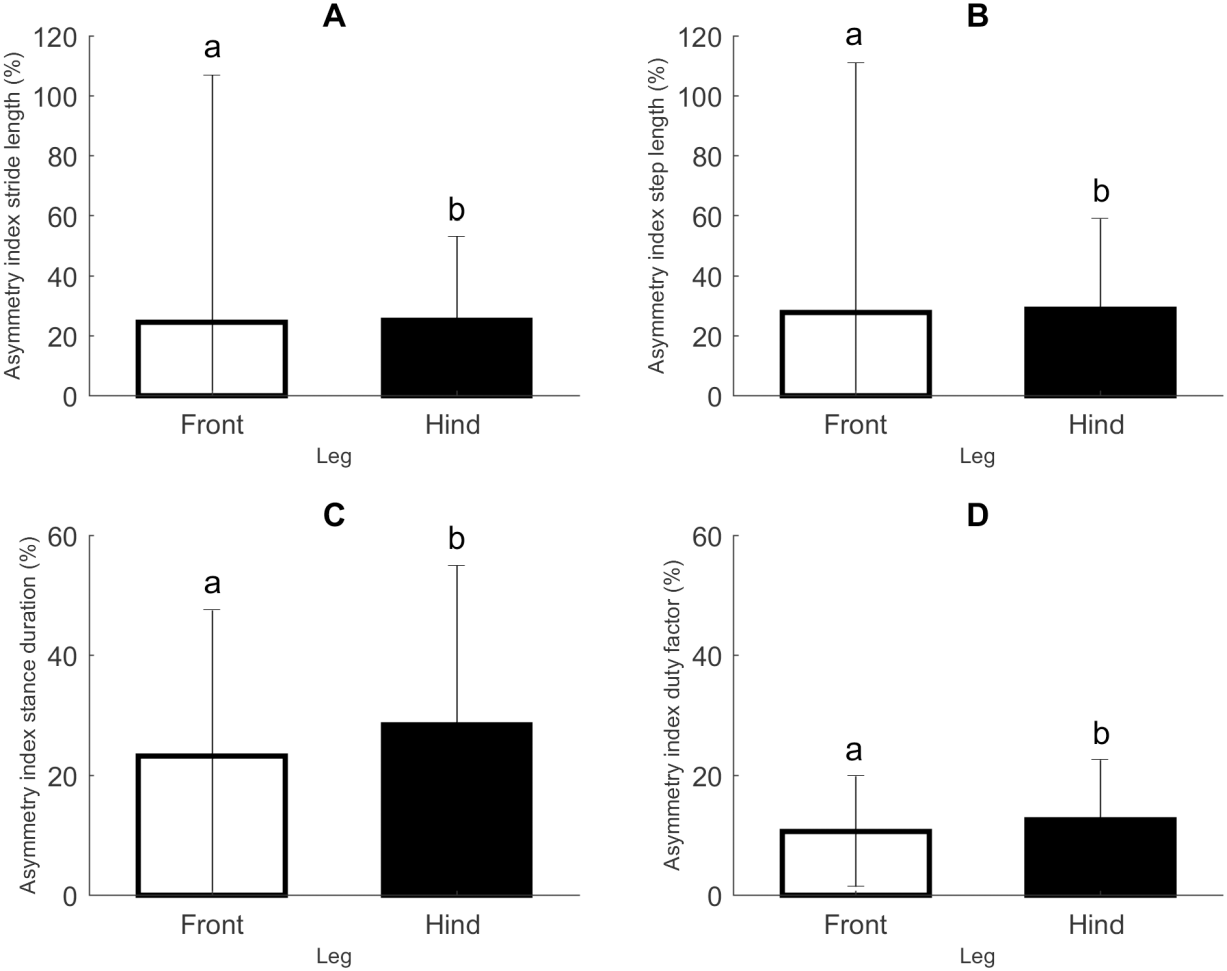
Gait stability—Leg differences. A. Asymmetry index stride length (AI_*l*stride_, n = 25, data points = 452). B. Asymmetry index step length (AI_*l*step_, n = 25, data points = 452). C. Asymmetry index stance duration (AI_*t*st_, n = 25, data points = 452). D. Asymmetry index duty factor (AI_df_, n = 25, data points = 452). All values are mean ± SD. Significant differences (linear mixed models, *p* ≤ 0.05) are indicated by different letters.

Taking a closer look at the leg pairs, we saw differences for AI_*f*_, AI_*l*stride_, AI_*l*step_, AI_*t*st_ and AI_df_ (Fig 6). However, for AI_*f*_ we only saw a difference among leg pairs for L piglets. In this group the front leg pair showed a lower AI_*f*_ of 15.07% (± 12.21), compared to the hind leg pair (22.38% (± 22.94); *p* = 0.0033). AI_*l*stride_ also showed a difference between the front and the hind leg pair (Fig 6A), though not limited to L piglets. The value for the front leg pair was 24.44% (± 82.45), while the hind leg pair showed an AI_*l*stride_ of 25.21% (± 27.99) (*p* = 0.0014). The front leg pair also showed a lower AI_*l*step_ (27.82% (± 83.18)) compared to the hind leg pair (28.95% (± 29.98), *p* = 0.0006, Fig 6B). Similar to AI_*l*stride_ and AI_*l*step_, front legs showed a lower AI_*t*st_ than hind legs (*p* = 0.0001; Fig 6C), 23.56% (± 24.31) and 28.42% (± 26.59), respectively. Contrary to this, we saw no effect of leg for AI_*t*sw_. With regard to AI_df_, the front leg pair again showed a higher degree of symmetry than the hind leg pair (AI_df_ = 10.71% (± 9.25) and 12.60% (± 10.08), respectively; Fig 6D).

### Spatio-temporal gait variables in relation to body mass

**Fig 7:**
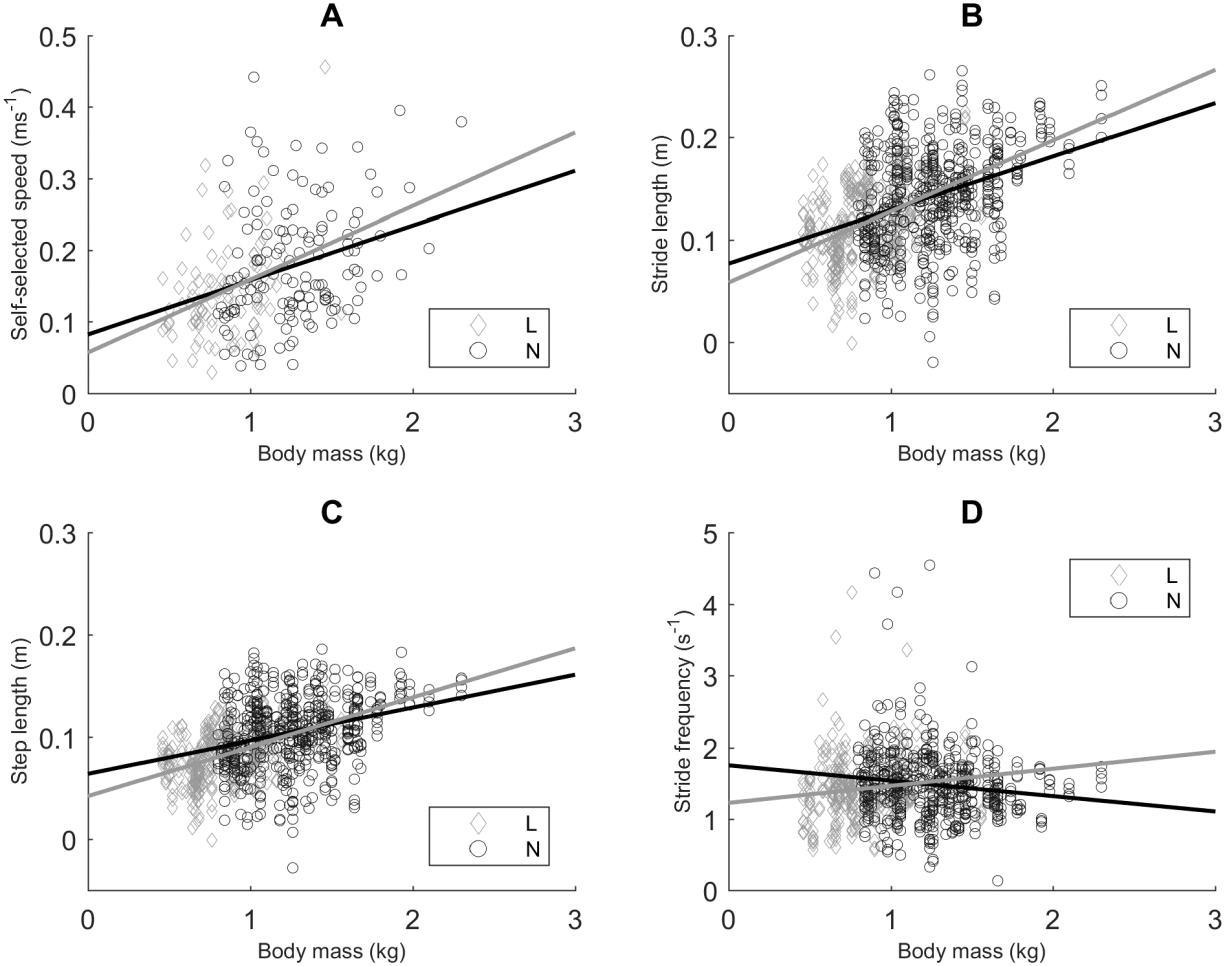
Spatio-temporal gait variables in function of body mass. A. Self-selected speed (*u*, n = 25, data points = 226). B. Stride length (*l*_stride_, n = 25, data points = 904). C. Step length (*l*_step_, n = 25, data points = 904). D. Stride frequency (*f*, n = 25, data points = 904). Scatter plot with regression lines for low birth weight/low vitality (L) and normal birth weight/normal vitality (N) piglets (linear mixed models).

Taking a closer look at the absolute variables in function of body mass (regardless of age, Fig 7), we saw that *u* for L and N piglets did not differ significantly, but in fact seemed to form a continuum, with *u* increasing with a greater body mass (*p* < 0.0001, Fig 7A). This significant effect of body mass was also visible for *l*_stride_ and *l*_step_, which also increased with body mass (*p* < 0.0001 for both variables; Fig 7B and 7C). However, for *l*_stride_ and *l*_step_ we did observe a significantly higher mean value for N piglets (0.14 m (± 0.05) and 0.11 m (± 0.04), respectively), compared to L piglets (0.12 m (± 0.04) and 0.08 m (± 0.03), respectively; *p* = 0.004 for *l*_stride_ and *p* = 0.0277 for *l*_step_). *f* in function of body mass was different for L and N piglets. For L piglets, body mass had no significant effect on *f*, i.e. *f* remained constant. However, for N piglets, we observed a decrease in *f* as body mass increased (*p* = 0.0126; Fig 7D).

## Discussion

Before addressing the specific questions and hypotheses mentioned in the Introduction section of this paper, it is important to take a closer look at the morphometrics of both groups because these inevitably influence several of the investigated gait variables.

During the first four days after birth the body mass of L piglets remains lower than that of N piglets, with the body mass for L piglets being on average 28.11% lower than that of N piglets. According to several studies, body mass at birth has a substantial impact on growth performance in later life [21, 22, 36, 37]. Moreover Dwyer *et al*. [38] and Gondret *et al*. [39] state that the difference in growth performance between lighter and heavier piglets may be attributed to a difference in food intake right after birth. However, our results indicate that body mass at birth does not impact growth performance in the first four days of life as the changes in body mass do not differ between L and N piglets. During the first four days after birth the milk production of the sow is ample for the whole litter, while, after day 8 of lactation (in case of a litter of 10 piglets and more) milk production of the sow begins to limit progeny growth [40]. From this point on, differences in growth between L and N piglets might be more apparent.

The observed difference in *H* between L and N mainly reflects size differences, with a higher body mass leading to a longer *H*. For both groups, changes in *H* during the first 28 hours are likely due to postural changes, since body mass remains constant during this period. Between 28 h and 96 h growth will be the main contributor to a longer *H* as was already suggested by Vanden Hole *et al*. [32]. An increase in *H*, be it by growth or postural changes, goes along with an increase in *u*, *l*_stride_ and *l*_step_ (see also Vanden Hole *et al*. [32]).

### Is motor performance different for L and N piglets at birth and during early development?

Consistent with our hypothesis, we see a reduced motor performance for L piglets, indicated by a lower *u* for L than for N piglets. This lower *u* can be attributed to a shorter *l*_stride_ for L piglets, in view of the equal *f* for both groups. Likely, this difference in motor performance can be attributed to the difference in *H* (because of size) between both groups. A longer *H* for N piglets leads to a larger *l*_stride_, which in turn leads to a higher *u*, compared to L piglets (given that L and N piglets show the same *f*). However, it might be advantageous for L piglets to attain a similar *u* to N piglets by increasing *f* in order to compensate for the shorter *l*_stride_. A first advantage of increasing their *u* might be a more balanced competition for teats with N piglets. Selection-related increase in litter size has led to a more severe teat competition [41]. L piglets tend to lose this competition to their heavier littermates due to their size and lack of vigor and, as a consequence, have a reduced milk intake [42]. A second advantage of L piglets being able to increase *u*, is that the chance of being crushed by the sow might be reduced. So then why don’t L piglets increase their *f* in order to attain a higher *u*?

The identical motor task of L and N piglets (*u’*, *f’* and *l*_stride_’) suggests that muscle recruitment (the fraction of activated fibers) takes place at the same level for both groups. If, however, L piglets were to increase *u* by means of a higher *f*, this recruitment level should increase disproportionally because faster walking would not only imply a higher relative load for the muscles, but faster walking would also mean faster contractions, hence less force produced per fiber. As such, the locomotor cost would be influenced directly [43], but the metabolic effect could even be more detrimental because of the decrease in efficiency (mechanical power/metabolic power) when contraction speed goes up [44]. Therefore we believe that, even if there would be room for a higher recruitment level (after all it seems plausible that in the newborn, feeble piglets, at *u* all fibers are already recruited, anyway) L piglet muscles may simply lack the required energy flow to increase *f*. Piglets, both L and N, are born with low energy reserves. A newborn pig is devoid of brown fat [45–48] but they do have a limited amount of glycogen pools in the muscles and the liver [45, 49, 50]. However, these energy reserves in the muscles seem to be proportional to size [49] which implies that L piglets start off their life with a smaller energy reserve than N piglets (Vanden Hole, preliminary observations). During the early postnatal period, energy levels increase rapidly [51], mainly via the intake of fatty acids [52–55]. However, as mentioned earlier, due to an increase in teat competition, the intake of milk (and hence fatty acids) is reduced in L piglets. The combination of no brown fat, lower glycogen reserves and a reduced fatty acid intake, complies and explains the observation that *f* remains unaffected in L piglets.

### Are neuromotor skills different at birth and is the associated neuromotor maturation different for L and N piglets?

To answer this question, we need to take a closer look at our three specific subquestions regarding the motor task, interlimb coordination and intralimb coordination.

#### Is voluntary locomotion for L piglets at *u* dynamically similar to N piglets after a few hours and is, in that case, the locomotion of both groups identical?

The motor task is similar for L and N piglets and stabilizes very quickly, which is indicated by *u’* reaching a stable value within 2 h after birth. For both categories, *u’* increases during these first 1 to 2 h after birth, which is accomplished by an increase in both its components *l*_stride_*’* and *f’*. *l*_stride_ increases up to and including 26 h, which can be attributed to an increase in *H* (due to postural changes and growth). *l*_stride_*’*, after an initial increase, already stabilizes between 2 h and 4 h after birth. During this short period of increase, neuromotor maturation takes place. *f*, on the other hand, remains invariable during early development, while *f’* shows a short increase during the first hour after birth, due to an increase in *H*. Interestingly, *f’* is lower for L piglets than for N piglet across all ages, though this does not cause an overall difference in *u’* for both groups. Contrary to our hypothesis, these results indicate that with regard to motor task, L and N piglets show an identical and equally quick maturation process.

#### Is the relative limb phasing (interlimb coordination) at birth and its postnatal development different for L and N piglets?

The lack of changes during early development and between L and N piglets supports our hypothesis based on the results on N piglets (published also in Vanden Hole *et al*. [32]), that interlimb coordination is indeed completely innate in pigs.

#### Is intralimb coordination at birth and its postnatal development different for L and N piglets?

We hypothesized a difference (both at birth and during subsequent development) in individual limb behavior between L and N piglets, but this does not seem to be the case (completely). First off, *l*_step_*’* and *h*_swmax_*’* are not different for L and N piglets, thus not confirming our hypothesis. In other words the linear dimensions of their step, relative to size, are equal between both groups during early development. In addition, the evolution of both *l*_step_*’* and *h*_swmax_*’* during early development shows the same pattern, which means neuromotor maturation in this regard is the same for L and N piglets.

However, *t*_*st*_*’* and *t*_*sw*_*’* are longer for L piglets, compared to N piglets, though afterwards they show the same maturation for both groups. This confirms our hypothesis only partly, since we don’t see a differential maturation between L and N piglets and the difference that is present at birth is maintained throughout early development. These different levels of *t*_*st*_*’* and *t*_*sw*_*’* at birth indicate a difference in neuromotor development *in utero*. The prolonged *t*_*st*_*’* and *t*_*sw*_*’* for L piglets lead to a lower *f’*, but (as mentioned earlier) not to a lower *u’*.

Both L and N piglets show a neuromotor maturation with regard to *t*_st_*’* of less than 1 h. During this first hour the duration of the stance phase is longer than at later ages. In Vanden Hole *et al*. [32] we propose that one of the reasons of a longer stance phase might be an increased need for support. This might be necessary during this first hour after birth, because the higher *h*_swmax_*’* might lead to a decreased stability. This is especially visible for the front legs, which, during this first hour after birth show an increase in *h*_swmax_*’* and *t*_st_*’*. For a more extensive discussion on front versus hind legs, see below.

Neuromotor maturation of the *t*_sw_*’* seems to take longer, with an initial decrease until the age of 4 h, after which (between the age of 4 and 6 h) *t*_sw_*’* stabilizes at a slightly higher level. This is the same pattern as described for N piglets in Vanden Hole *et al*. [32] though in that study, the shortest swing phase was observed at the age of 2 h. This modest difference is likely due to the size difference of the datasets used in both studies, with the current study having a considerably larger dataset.

df (stance duration in function of total stride duration) is the same for L and N piglets. This shows that, though *t*_st_*’* in itself is longer for L piglets, the *t*_st_ relative to the duration of the entire stride, is the same for L and N piglets. This is in accordance with results by Biewener [56], who found df to remain constant for differently sized animals (mouse, chipmunk, squirrel, dog and horse) and Vilensky *et al*. [57] who found no correlation for hind limb df and size for vervet monkeys. With regard to neuromotor maturation, L and N piglets show the same pattern with a higher df during the first 2 h after birth and a stabilization afterwards. During these first 2 h, we also see a prolonged *t*_sw_*’*. When *t*_sw_*’* increases, the duration that the front or rear end is only supported by one leg, increases as well. These supporting legs experience an increased amount of force. However, an increase in df reduces the peak ground force that is exerted on a limb during the stance phase [58, 59]. This leads to a reduction in forces (bending and compressing) acting on a bone [59]. We propose this might be at play during the first 2 h, when the positioning of the legs (indicated by *H*) is not yet optimal for walking. Afterwards, *t*_sw_*’* decreases further, in order to walk as energy-efficient as possible [32].

### Is the variability of the gait pattern at birth and during early development different for L and N piglets?

Contrary to our hypothesis, L and N piglets show no difference in AIs, indicating the same development and degree of stability in both groups. In accordance with Vanden Hole *et al*. [32] the variability of the early gait pattern decreases with age, leading to a stable gait pattern (with an AI of around 10%, similar to adult pigs [60]) within a few hours of birth. In this earlier study, most AIs reach a consistent value around 8 h after birth, though with the larger dataset used in the current study, it looks like a stable gait develops already within 2 h of birth. The only exception is AI_*l*stride_, which seems to take a little longer (between 8 and 24 h) to reach a constant value.

One cannot discuss gait stability without also considering possible differences among leg pairs. As argued in our previous study [32] the front and hind legs show a difference in limb-specific characteristics because of their body build. With pigs having a body mass distribution of about 60% on the front limbs and 40% on the hind limbs [60, 61], this leads to a difference in df, *t*_s_*’* and *t*_sw_*’* between front and hind limbs. Front legs spend a longer time on the ground than hind legs. As a consequence they also exhibit a longer *l*_step_*’*, which is the displacement of the COM during the stance phase.

However, for both N and L piglets we see differences between front and hind legs that cannot solely be explained by this difference in body build. *h*_swmax_*’* shows a different development for front legs and hind legs. During the first hours piglets lift up their front legs higher than during the rest of the study period. In addition, we see that front legs display a higher degree of symmetry compared to hind legs (as indicated by values of AI_*l*stride_, AI_*l*step_, AI_*t*st_ and AI_df_), a difference that is most pronounced during the first few hours. This makes sense if we consider the front legs to take up an exploratory role. The front limbs are the first to encounter modifications to the environment (different substrates, obstacles etc.) and must be the first to adjust their movements [34]. An increase in *h*_swmax_*’* might help with exploring the environment, while a higher degree of symmetry increases stability even if obstacles are encountered.

### Can L piglets be considered a smaller version of N piglets?

The *u*, *l*_stride_ and *l*_step_ of L and N piglets increase in function of a higher body mass. For *l*_stride_ and *l*_step_ we see a lower mean value for L piglets than for N piglets. For *u* this is not this case, though this is likely due to a smaller number of data points, because *u* is calculated per piglet and not per leg. For these three variables we can state that L piglets behave as a smaller version of N piglets.

With regard to *f*, we see a different relation with body mass for L and N piglets. In L piglets *f* remains constant in spite of an increase in body mass, which is in accordance with our earlier proposed theory with regards to the inability of L piglets to adjust their *f*. However, for N piglets *f* decreases in function of body mass. This might indicate that N piglets, given that *l*_stride_ increases because of an increased body mass, lower *f* to increase the energetic efficiency of walking (also see the Discussion on ‘motor performance’).

## Conclusions

We noted a reduced motor performance for L piglets with a lower *l*_stride_ (caused by a shorter *H)*, leading to a lower *u*. For *u*, *l*_stride_ and *l*_*step*_ we observed an increase in function of body mass, indicating L piglets might be considered a smaller version of N piglets. At birth, the motor task (*u’*, *f’* and *l*_stride_’), the linear aspects of limb-specific coordination (*l*_step_’ and *h*_swmax_’), the relative limb-phasing and stability of the gait (left-right asymmetry) appear similar for L and N piglets, while we do see different levels of *t*_*st*_*’* and *t*_*sw*_*’*. During the four days after birth, neuromotor maturation shows the same pattern for L and N piglets.

As such, we believe that intrauterine crowding affects locomotion mainly by impairing growth *in utero* of L piglets, which after birth negatively affects motor performance. In addition, we see a slight difference in neuromotor skills at birth. This implies a difference in neuromotor development *in utero*, indicating that L piglets might be considered a fictitious younger version of N piglets. However, it does not seem that intrauterine crowding affects neuromotor maturation after birth.

## Supporting information

S1 Table Morphometrics. (PDF)

S2 Table Absolute self-selected speed. (PDF)

S3 Table Absolute spatio-temporal gait variables. (PDF)

S4 Table Normalized self-selected speed. (PDF)

S5 Table Normalized spatio-temporal gait variables. (PDF)

S6 Table Asymmetry indices. (PDF)

S7 Table Interlimb variables. (PDF)
