# Supplementary material for: Does intrauterine crowding affect locomotor development? A comparative study of motor performance, neuromotor maturation and gait variability among piglets that differ in birth weight and vitality
Source: PLoS One. 2018 Apr 24;13(4):e0195961. doi: 10.1371/journal.pone.0195961 (PMC5915318; doi:10.1371/journal.pone.0195961)
Supplement: S1 Table — (PDF) [file pone.0195961.s001.pdf]

S1. MORPHOMETRICS

PIGLET	SOW	CATEGORY	GENDER	VITALITY	AGE (h)	BODY MASS AT BIRTH (kg)	BODY MASS (kg)	FUNCTIONAL HIND LIMB LENGTH (m)
151301	F1816	L	F	2	1	0.64	0.64	0.104220921
151301	F1816	L	F	2	2	0.64	0.62	0.115379616
151301	F1816	L	F	2	4	0.64	0.6	0.120228173
151301	F1816	L	F	2	6	0.64	0.66	0.118968996
151301	F1816	L	F	2	8	0.64	0.64	0.116822734
151301	F1816	L	F	2	24	0.64	0.7	0.140755993
151301	F1816	L	F	2	26	0.64	0.7	0.141438645
151301	F1816	L	F	2	28	0.64	0.72	0.134487534
151301	F1816	L	F	2	96	0.64	1.04	0.147759391
151302	F1816	L	F	2	0	0.66	0.66	0.101070747
151302	F1816	L	F	2	1	0.66	0.66	0.121365388
151302	F1816	L	F	2	2	0.66	0.64	0.12643264
151302	F1816	L	F	2	4	0.66	0.68	0.134803939
151302	F1816	L	F	2	6	0.66	0.68	0.125411363
151302	F1816	L	F	2	8	0.66	0.68	0.136653253
151302	F1816	L	F	2	24	0.66	0.74	0.138748216
151302	F1816	L	F	2	26	0.66	0.76	0.134663575
151302	F1816	L	F	2	28	0.66	0.74	0.139496641
151302	F1816	L	F	2	96	0.66	1.04	0.153379161
151306	F1349	L	F	2	4	0.7	0.7	0.101646335
151306	F1349	L	F	2	6	0.7	0.68	0.099826782
151309	F943	L	F	2	1	0.54	0.52	0.085867385
151309	F943	L	F	2	2	0.54	0.52	0.086778868
151309	F943	L	F	2	4	0.54	0.5	0.101267227
151309	F943	L	F	2	6	0.54	0.5	0.103868387
151309	F943	L	F	2	8	0.54	0.5	0.090091662
151309	F943	L	F	2	24	0.54	0.48	0.107756742
151309	F943	L	F	2	26	0.54	0.46	0.113493001
151309	F943	L	F	2	28	0.54	0.46	0.102656103

151310	F943	L	F	2	0	0.64	0.64	0.08717499
151310	F943	L	F	2	1	0.64	0.66	0.077459219
151310	F943	L	F	2	2	0.64	0.66	0.096014315
151310	F943	L	F	2	4	0.64	0.68	0.100916887
151310	F943	L	F	2	6	0.64	0.68	0.1174368
151310	F943	L	F	2	8	0.64	0.68	0.121610689
151310	F943	L	F	2	24	0.64	0.72	0.125716726
151310	F943	L	F	2	26	0.64	0.76	0.133612874
151310	F943	L	F	2	28	0.64	0.76	0.131471009
151310	F943	L	F	2	96	0.64	1.16	0.145842198
152686	F1158	L	F	2	1	0.75	0.76	0.101037501
152686	F1158	L	F	2	2	0.75	0.76	0.12054223
152686	F1158	L	F	2	4	0.75	0.74	0.152861263
152686	F1158	L	F	2	6	0.75	0.76	0.144623682
152686	F1158	L	F	2	8	0.75	0.74	0.164921281
152686	F1158	L	F	2	24	0.75	0.78	0.172385224
152686	F1158	L	F	2	26	0.75	0.82	0.17762215
152686	F1158	L	F	2	28	0.75	0.8	0.173650395
154983	F1571	L	F	2	4	0.62	0.58	0.143236108
154983	F1571	L	F	2	6	0.62	0.58	0.131147486
154983	F1571	L	F	2	8	0.62	0.56	0.155714154
159811	F1541	L	M	1	1	0.91	0.9	0.129030184
159811	F1541	L	M	1	2	0.91	0.94	0.15182489
159811	F1541	L	M	1	4	0.91	0.98	0.151795536
159811	F1541	L	M	1	6	0.91	0.96	0.122345133
159811	F1541	L	M	1	8	0.91	0.94	0.150056621
159811	F1541	L	M	1	24	0.91	0.86	0.159478549
159811	F1541	L	M	1	26	0.91	0.86	0.159208769
159811	F1541	L	M	1	28	0.91	0.86	0.155476256
159811	F1541	L	M	1	96	0.91	1.08	0.165680345
159828	F1546	L	F	2	0	1.04	1.04	0.134794567
159828	F1546	L	F	2	1	1.04	1.02	0.148905626
159828	F1546	L	F	2	2	1.04	1.02	0.154411723

159828	F1546	L	F	2	4	1.04	1.1	0.171162888
159828	F1546	L	F	2	6	1.04	1.06	0.1565303
159828	F1546	L	F	2	8	1.04	1.1	0.165295049
159828	F1546	L	F	2	24	1.04	1.02	0.170012377
159828	F1546	L	F	2	26	1.04	1.06	0.167238712
159828	F1546	L	F	2	28	1.04	1.08	0.167467731
159828	F1546	L	F	2	96	1.04	1.46	0.169002504
160446	F1546	L	M	2	0	1.02	1.02	0.1023001
160446	F1546	L	M	2	1	1.02	1.02	0.136177281
160446	F1546	L	M	2	2	1.02	1.02	0.152710164
160446	F1546	L	M	2	4	1.02	1.04	0.148298417
160446	F1546	L	M	2	6	1.02	1.04	0.144393995
160446	F1546	L	M	2	8	1.02	1.04	0.159631519
160446	F1546	L	M	2	24	1.02	1.08	0.164712825
160446	F1546	L	M	2	26	1.02	1.12	0.163796296
160446	F1546	L	M	2	28	1.02	1.12	0.159132367
160446	F1546	L	M	2	96	1.02	1.56	0.168368474
160639	F1546	L	F	2	0	0.9	0.9	0.111454923
160639	F1546	L	F	2	1	0.9	0.88	0.121477258
160639	F1546	L	F	2	2	0.9	0.9	0.134208079
160639	F1546	L	F	2	4	0.9	0.86	0.124015579
160639	F1546	L	F	2	6	0.9	0.88	0.137334501
160639	F1546	L	F	2	8	0.9	0.86	0.139815228
160639	F1546	L	F	2	24	0.9	0.82	0.15480216
160639	F1546	L	F	2	26	0.9	0.86	0.158308126
160639	F1546	L	F	2	28	0.9	0.84	0.158762427
160639	F1546	L	F	2	96	0.9	1.2	0.175051844
151303	F943	N	F	4	0	0.84	0.84	0.084363432
151303	F943	N	F	4	1	0.84	0.84	0.109321582
151303	F943	N	F	4	2	0.84	0.8	0.129456718
151303	F943	N	F	4	4	0.84	0.84	0.135533384
151303	F943	N	F	4	6	0.84	0.84	0.129301922
151303	F943	N	F	4	8	0.84	0.82	0.135593568

151303	F943	N	F	4	24	0.84	0.86	0.142625956
151303	F943	N	F	4	26	0.84	0.86	0.141997668
151303	F943	N	F	4	28	0.84	0.86	0.14479637
151303	F943	N	F	4	96	0.84	1.18	0.238833598
151307	F943	N	F	3	0	1.06	1.06	0.088200336
151307	F943	N	F	3	1	1.06	1.06	0.155939118
151307	F943	N	F	3	2	1.06	1.08	0.16310486
151307	F943	N	F	3	4	1.06	1.1	0.163811296
151307	F943	N	F	3	6	1.06	1.14	0.158866163
151307	F943	N	F	3	8	1.06	1.16	0.161274859
151307	F943	N	F	3	24	1.06	1.18	0.162216733
151307	F943	N	F	3	26	1.06	1.2	0.172707507
151307	F943	N	F	3	28	1.06	1.18	0.188278832
151307	F943	N	F	3	96	1.06	1.66	0.287985246
152750	F1571	N	F	4	0	0.98	0.98	0.068045368
152750	F1571	N	F	4	1	0.98	0.98	0.126305084
152750	F1571	N	F	4	2	0.98	0.98	0.18918154
152750	F1571	N	F	4	4	0.98	1	0.177373908
152750	F1571	N	F	4	6	0.98	1.04	0.147434011
152750	F1571	N	F	4	8	0.98	1.04	0.194255302
152750	F1571	N	F	4	24	0.98	1.04	0.202779029
152750	F1571	N	F	4	26	0.98	1.02	0.199321913
152750	F1571	N	F	4	28	0.98	1	0.197286005
152750	F1571	N	F	4	96	0.98	1.32	0.211510622
152776	F1571	N	M	3	0	1.26	1.26	0.113696326
152776	F1571	N	M	3	1	1.26	1.26	0.121813482
152776	F1571	N	M	3	2	1.26	1.36	0.188230604
152776	F1571	N	M	3	4	1.26	1.34	0.181651502
152776	F1571	N	M	3	6	1.26	1.36	0.190002951
152776	F1571	N	M	3	8	1.26	1.4	0.195090726
152776	F1571	N	M	3	24	1.26	1.42	0.211775002
152776	F1571	N	M	3	26	1.26	1.4	0.204173687
152776	F1571	N	M	3	28	1.26	1.44	0.205186904

152776	F1571	N	M	3	96	1.26	1.93	0.222395711
154850	F998	N	F	3	0	1.06	1.06	0.106154242
154850	F998	N	F	3	1	1.06	1	0.153573645
154850	F998	N	F	3	2	1.06	1.02	0.179766505
154850	F998	N	F	3	4	1.06	1.04	0.178770874
154850	F998	N	F	3	6	1.06	1.04	0.192350189
154850	F998	N	F	3	8	1.06	1.06	0.183703948
154850	F998	N	F	3	24	1.06	1.1	0.197895335
154850	F998	N	F	3	26	1.06	1.1	0.189303928
154850	F998	N	F	3	28	1.06	1.08	0.205884475
154850	F998	N	F	3	96	1.06	1.66	0.216778913
155005	F1158	N	M	3	0	0.94	0.94	0.075241012
155005	F1158	N	M	3	1	0.94	0.9	0.131258169
155005	F1158	N	M	3	2	0.94	0.9	0.156358868
155005	F1158	N	M	3	4	0.94	0.88	0.159076102
155005	F1158	N	M	3	6	0.94	0.96	0.172913145
155005	F1158	N	M	3	8	0.94	0.92	0.154537769
155005	F1158	N	M	3	24	0.94	1.04	0.183468167
155005	F1158	N	M	3	26	0.94	1.04	0.182055912
155005	F1158	N	M	3	28	0.94	1.02	0.182401693
155005	F1158	N	M	3	96	0.94	1.14	0.194429827
155029	F1571	N	F	4	0	1.24	1.24	0.117557359
155029	F1571	N	F	4	1	1.24	1.22	0.141041793
155029	F1571	N	F	4	2	1.24	1.26	0.182684094
155029	F1571	N	F	4	4	1.24	1.24	0.205867398
155029	F1571	N	F	4	8	1.24	1.26	0.206332897
155029	F1571	N	F	4	24	1.24	1.22	0.212543038
155029	F1571	N	F	4	26	1.24	1.24	0.207846332
155029	F1571	N	F	4	28	1.24	1.26	0.206041877
155029	F1571	N	F	4	96	1.24	1.52	0.215428688
155362	F1158	N	M	3	0	0.96	0.96	0.15091696
155362	F1158	N	M	3	1	0.96	0.94	0.122702199
155362	F1158	N	M	3	2	0.96	0.96	0.118855062

155362	F1158	N	M	3	4	0.96	0.92	0.156524982
155362	F1158	N	M	3	6	0.96	0.9	0.152146281
155362	F1158	N	M	3	8	0.96	0.9	0.165710185
155362	F1158	N	M	3	24	0.96	0.88	0.170352951
155362	F1158	N	M	3	26	0.96	0.86	0.189330469
155362	F1158	N	M	3	28	0.96	0.84	0.177767284
160013	F1546	N	M	3	0	1.26	1.26	0.118718992
160013	F1546	N	M	3	1	1.26	1.22	0.15838951
160013	F1546	N	M	3	2	1.26	1.24	0.175732113
160013	F1546	N	M	3	4	1.26	1.3	0.174832464
160013	F1546	N	M	3	6	1.26	1.32	0.180990427
160013	F1546	N	M	3	8	1.26	1.32	0.174402484
160013	F1546	N	M	3	24	1.26	1.3	0.186360326
160013	F1546	N	M	3	26	1.26	1.34	0.179792622
160013	F1546	N	M	3	28	1.26	1.34	0.181890623
160013	F1546	N	M	3	96	1.26	1.78	0.196476529
160021	F1768	N	M	4	0	1.36	1.36	0.124909043
160021	F1768	N	M	4	1	1.36	1.36	0.15737483
160021	F1768	N	M	4	2	1.36	1.42	0.151515239
160021	F1768	N	M	4	4	1.36	1.48	0.159044509
160021	F1768	N	M	4	6	1.36	1.46	0.159820265
160021	F1768	N	M	4	8	1.36	1.44	0.16189204
160021	F1768	N	M	4	24	1.36	1.44	0.178756371
160021	F1768	N	M	4	26	1.36	1.46	0.15859578
160021	F1768	N	M	4	28	1.36	1.48	0.168150279
160021	F1768	N	M	4	96	1.36	1.98	0.189215793
160096	F1034	N	F	4	0	1.5	1.5	0.15770522
160096	F1034	N	F	4	1	1.5	1.52	0.196568706
160096	F1034	N	F	4	2	1.5	1.5	0.193750393
160096	F1034	N	F	4	4	1.5	1.6	0.184216913
160096	F1034	N	F	4	6	1.5	1.6	0.19024093
160096	F1034	N	F	4	8	1.5	1.6	0.184584688
160096	F1034	N	F	4	24	1.5	1.64	0.199470537

160096	F1034	N	F	4	26	1.5	1.64	0.190694992
160096	F1034	N	F	4	28	1.5	1.66	0.196518511
160096	F1034	N	F	4	96	1.5	1.92	0.210689165
160121	F1546	N	F	4	0	1.64	1.64	0.183833157
160121	F1546	N	F	4	1	1.64	1.6	0.186891109
160121	F1546	N	F	4	2	1.64	1.66	0.18290675
160121	F1546	N	F	4	4	1.64	1.64	0.180758293
160121	F1546	N	F	4	6	1.64	1.66	0.192272096
160121	F1546	N	F	4	8	1.64	1.68	0.185399033
160121	F1546	N	F	4	24	1.64	1.74	0.205462503
160121	F1546	N	F	4	26	1.64	1.78	0.20110215
160121	F1546	N	F	4	28	1.64	1.8	0.20719729
160121	F1546	N	F	4	96	1.64	2.3	0.211603902
160153	F1768	N	M	3	0	1.18	1.18	0.11737349
160153	F1768	N	M	3	1	1.18	1.2	0.168212656
160153	F1768	N	M	3	2	1.18	1.24	0.170530161
160153	F1768	N	M	3	4	1.18	1.3	0.166257984
160153	F1768	N	M	3	6	1.18	1.26	0.166482623
160153	F1768	N	M	3	8	1.18	1.26	0.173798196
160153	F1768	N	M	3	24	1.18	1.28	0.175162478
160153	F1768	N	M	3	26	1.18	1.22	0.189603373
160153	F1768	N	M	3	28	1.18	1.26	0.182811122
160153	F1768	N	M	3	96	1.18	1.6	0.187703249
160777	F1745	N	M	3	1	1.38	1.38	0.147867747
160777	F1745	N	M	3	2	1.38	1.38	0.163389816
160777	F1745	N	M	3	4	1.38	1.5	0.171415496
160777	F1745	N	M	3	6	1.38	1.46	0.142217652
160777	F1745	N	M	3	8	1.38	1.46	0.180587996
160777	F1745	N	M	3	24	1.38	1.44	0.175429626
160777	F1745	N	M	3	26	1.38	1.42	0.188987295
160777	F1745	N	M	3	28	1.38	1.44	0.181801372
160777	F1745	N	M	3	96	1.38	2.1	0.196697193
