# Supplementary material for: Does intrauterine crowding affect locomotor development? A comparative study of motor performance, neuromotor maturation and gait variability among piglets that differ in birth weight and vitality
Source: PLoS One. 2018 Apr 24;13(4):e0195961. doi: 10.1371/journal.pone.0195961 (PMC5915318; doi:10.1371/journal.pone.0195961)
Supplement: S2 Table — (PDF) [file pone.0195961.s002.pdf]

S2. ABSOLUTE SELF-SELECTED SPEED

PIGLET	SOW	CATEGORY	GENDER	AGE (h)	SELF-SELECTED SPEED (ms^{-1})
151301	F1816	L	F	1	0.136274038
151301	F1816	L	F	2	0.126327161
151301	F1816	L	F	4	0.222376072
151301	F1816	L	F	6	0.16753931
151301	F1816	L	F	8	0.097096593
151301	F1816	L	F	24	0.114747656
151301	F1816	L	F	26	0.283389038
151301	F1816	L	F	28	0.318102109
151301	F1816	L	F	96	0.086834113
151302	F1816	L	F	0	0.109881455
151302	F1816	L	F	1	0.064299801
151302	F1816	L	F	2	0.14663588
151302	F1816	L	F	4	0.156813427
151302	F1816	L	F	6	0.129999849
151302	F1816	L	F	8	0.115788437
151302	F1816	L	F	24	0.139692692
151302	F1816	L	F	26	0.10209276
151302	F1816	L	F	28	0.064278381
151302	F1816	L	F	96	0.059775569
151306	F1349	L	F	4	0.116489751
151306	F1349	L	F	6	0.094383592
151309	F943	L	F	1	0.045938919
151309	F943	L	F	2	0.080279592
151309	F943	L	F	4	0.100306747
151309	F943	L	F	6	0.097540315
151309	F943	L	F	8	0.117427243
151309	F943	L	F	24	0.092194931
151309	F943	L	F	26	0.160256494
151309	F943	L	F	28	0.087788076

151310	F943	L	F	0	0.045764434
151310	F943	L	F	1	0.100192616
151310	F943	L	F	2	0.101698855
151310	F943	L	F	4	0.094206394
151310	F943	L	F	6	0.097623982
151310	F943	L	F	8	0.074184365
151310	F943	L	F	24	0.110954217
151310	F943	L	F	26	0.128050755
151310	F943	L	F	28	0.156732334
151310	F943	L	F	96	0.150544603
152686	F1158	L	F	1	0.029094344
152686	F1158	L	F	2	0.094489477
152686	F1158	L	F	4	0.15778092
152686	F1158	L	F	6	0.191984049
152686	F1158	L	F	8	0.224429612
152686	F1158	L	F	24	0.062199454
152686	F1158	L	F	26	0.182057895
152686	F1158	L	F	28	0.141359834
154983	F1571	L	F	4	0.174459347
154983	F1571	L	F	6	0.124273235
154983	F1571	L	F	8	0.147645323
159811	F1541	L	M	1	0.085206104
159811	F1541	L	M	2	0.06922846
159811	F1541	L	M	4	0.15773506
159811	F1541	L	M	6	0.142958916
159811	F1541	L	M	8	0.114010749
159811	F1541	L	M	24	0.155866781
159811	F1541	L	M	26	0.136371738
159811	F1541	L	M	28	0.252733035
159811	F1541	L	M	96	0.216929791
159828	F1546	L	F	0	0.11962916
159828	F1546	L	F	1	0.195407188
159828	F1546	L	F	2	0.09655706

159828	F1546	L	F	4	0.184073129
159828	F1546	L	F	6	0.118591406
159828	F1546	L	F	8	0.243554009
159828	F1546	L	F	24	0.194433524
159828	F1546	L	F	26	0.209248451
159828	F1546	L	F	28	0.293876516
159828	F1546	L	F	96	0.455573421
160446	F1546	L	M	0	0.071499177
160446	F1546	L	M	1	0.097210483
160446	F1546	L	M	2	0.180477742
160446	F1546	L	M	4	0.125656728
160446	F1546	L	M	6	0.131950156
160446	F1546	L	M	8	0.177918645
160446	F1546	L	M	24	0.131692476
160446	F1546	L	M	26	0.155305906
160446	F1546	L	M	28	0.117407236
160446	F1546	L	M	96	0.111584865
160639	F1546	L	F	0	0.0673119
160639	F1546	L	F	1	0.065428332
160639	F1546	L	F	2	0.126078704
160639	F1546	L	F	4	0.129910098
160639	F1546	L	F	6	0.255280387
160639	F1546	L	F	8	0.282325078
160639	F1546	L	F	24	0.159157356
160639	F1546	L	F	26	0.197939299
160639	F1546	L	F	28	0.154670784
160639	F1546	L	F	96	0.193569302
151303	F943	N	F	0	0.054467551
151303	F943	N	F	1	0.087960105
151303	F943	N	F	2	0.111505949
151303	F943	N	F	4	0.105374914
151303	F943	N	F	6	0.112467744
151303	F943	N	F	8	0.12046887

151303	F943	N	F	24	0.0813318
151303	F943	N	F	26	0.10866714
151303	F943	N	F	28	0.146334323
151303	F943	N	F	96	0.063173199
151307	F943	N	F	0	0.04003208
151307	F943	N	F	1	0.186513981
151307	F943	N	F	2	0.147731153
151307	F943	N	F	4	0.222442855
151307	F943	N	F	6	0.166809452
151307	F943	N	F	8	0.195319012
151307	F943	N	F	24	0.227763422
151307	F943	N	F	26	0.226316332
151307	F943	N	F	28	0.203058291
151307	F943	N	F	96	0.129595876
152750	F1571	N	F	0	0.054685439
152750	F1571	N	F	1	0.131175384
152750	F1571	N	F	2	0.229684087
152750	F1571	N	F	4	0.144924376
152750	F1571	N	F	6	0.185284439
152750	F1571	N	F	8	0.133855097
152750	F1571	N	F	24	0.154268595
152750	F1571	N	F	26	0.281980591
152750	F1571	N	F	28	0.364572033
152750	F1571	N	F	96	0.295633365
152776	F1571	N	M	0	0.154949233
152776	F1571	N	M	1	0.123068834
152776	F1571	N	M	2	0.211670442
152776	F1571	N	M	4	0.107966006
152776	F1571	N	M	6	0.218482594
152776	F1571	N	M	8	0.151601252
152776	F1571	N	M	24	0.214115142
152776	F1571	N	M	26	0.284483403
152776	F1571	N	M	28	0.342270205

152776	F1571	N	M	96	0.165810932
154850	F998	N	F	0	0.05969584
154850	F998	N	F	1	0.080737982
154850	F998	N	F	2	0.052370503
154850	F998	N	F	4	0.186685447
154850	F998	N	F	6	0.236107324
154850	F998	N	F	8	0.149882879
154850	F998	N	F	24	0.197547585
154850	F998	N	F	26	0.267601412
154850	F998	N	F	28	0.336900841
154850	F998	N	F	96	0.343803522
155005	F1158	N	M	0	0.038249551
155005	F1158	N	M	1	0.058811777
155005	F1158	N	M	2	0.113293248
155005	F1158	N	M	4	0.114820488
155005	F1158	N	M	6	0.153151645
155005	F1158	N	M	8	0.162288044
155005	F1158	N	M	24	0.253925862
155005	F1158	N	M	26	0.351139
155005	F1158	N	M	28	0.441656401
155005	F1158	N	M	96	0.310893308
155029	F1571	N	F	0	0.06678178
155029	F1571	N	F	1	0.089887092
155029	F1571	N	F	2	0.125813511
155029	F1571	N	F	4	0.127535085
155029	F1571	N	F	8	0.072281033
155029	F1571	N	F	24	0.215822126
155029	F1571	N	F	26	0.108595539
155029	F1571	N	F	28	0.228491793
155029	F1571	N	F	96	0.117817616
155362	F1158	N	M	0	0.252329664
155362	F1158	N	M	1	0.129737513
155362	F1158	N	M	2	0.168368061

155362	F1158	N	M	4	0.196351392
155362	F1158	N	M	6	0.117469964
155362	F1158	N	M	8	0.116915152
155362	F1158	N	M	24	0.15771284
155362	F1158	N	M	26	0.324716453
155362	F1158	N	M	28	0.288359066
160013	F1546	N	M	0	0.040187276
160013	F1546	N	M	1	0.160114223
160013	F1546	N	M	2	0.185635882
160013	F1546	N	M	4	0.136826706
160013	F1546	N	M	6	0.122432683
160013	F1546	N	M	8	0.225190304
160013	F1546	N	M	24	0.196681844
160013	F1546	N	M	26	0.149462668
160013	F1546	N	M	28	0.162689951
160013	F1546	N	M	96	0.164301058
160021	F1768	N	M	0	0.096897229
160021	F1768	N	M	1	0.133903347
160021	F1768	N	M	2	0.15146267
160021	F1768	N	M	4	0.134043365
160021	F1768	N	M	6	0.136762945
160021	F1768	N	M	8	0.228246297
160021	F1768	N	M	24	0.280595121
160021	F1768	N	M	26	0.140312934
160021	F1768	N	M	28	0.287427237
160021	F1768	N	M	96	0.286904146
160096	F1034	N	F	0	0.115664773
160096	F1034	N	F	1	0.128591157
160096	F1034	N	F	2	0.247921909
160096	F1034	N	F	4	0.209188993
160096	F1034	N	F	6	0.222368064
160096	F1034	N	F	8	0.117032633
160096	F1034	N	F	24	0.173526152

160096	F1034	N	F	26	0.238794606
160096	F1034	N	F	28	0.209741268
160096	F1034	N	F	96	0.394919379
160121	F1546	N	F	0	0.104560506
160121	F1546	N	F	1	0.19888822
160121	F1546	N	F	2	0.251986719
160121	F1546	N	F	4	0.199509758
160121	F1546	N	F	6	0.170461489
160121	F1546	N	F	8	0.148456893
160121	F1546	N	F	24	0.305791613
160121	F1546	N	F	26	0.280795687
160121	F1546	N	F	28	0.220157868
160121	F1546	N	F	96	0.379094492
160153	F1768	N	M	0	0.104473017
160153	F1768	N	M	1	0.125650648
160153	F1768	N	M	2	0.117117212
160153	F1768	N	M	4	0.135390197
160153	F1768	N	M	6	0.100680428
160153	F1768	N	M	8	0.304548472
160153	F1768	N	M	24	0.345860726
160153	F1768	N	M	26	0.250584899
160153	F1768	N	M	28	0.256310602
160153	F1768	N	M	96	0.165964567
160777	F1745	N	M	1	0.092499238
160777	F1745	N	M	2	0.121106508
160777	F1745	N	M	4	0.098033495
160777	F1745	N	M	6	0.131496718
160777	F1745	N	M	8	0.130295716
160777	F1745	N	M	24	0.151928753
160777	F1745	N	M	26	0.194011258
160777	F1745	N	M	28	0.233139249
160777	F1745	N	M	96	0.202443587
