# Supplementary material for: Does intrauterine crowding affect locomotor development? A comparative study of motor performance, neuromotor maturation and gait variability among piglets that differ in birth weight and vitality
Source: PLoS One. 2018 Apr 24;13(4):e0195961. doi: 10.1371/journal.pone.0195961 (PMC5915318; doi:10.1371/journal.pone.0195961)
Supplement: S3 Table — (PDF) [file pone.0195961.s003.pdf]

S3. ABSOLUTE SPATIO-TEMPORAL GAIT VARIABLES

PIGLET	SOW	CATEGORY	GENDER	AGE	LEG	STRIDE FREQ (s^{-1})	STRIDE LENGTH (m)	STEP LENGTH (m)
151301	F1816	L	F	1	LF	1.270913771	0.103662214	0.082860927
151301	F1816	L	F	1	RF	1.362007168	0.070196014	0.072240021
151301	F1816	L	F	1	LH	1.12487361	0.070215841	0.046517822
151301	F1816	L	F	1	RH	1.352813853	0.088543926	0.058324345
151301	F1816	L	F	2	LF	1.668520578	0.096515041	0.07052522
151301	F1816	L	F	2	RF	1.503094607	0.065172748	0.063497594
151301	F1816	L	F	2	LH	0.685820204	0.116663504	0.088272242
151301	F1816	L	F	2	RH	1.471861472	0.091998701	0.071806878
151301	F1816	L	F	4	LF	2.003205128	0.105542428	0.046999046
151301	F1816	L	F	4	RF	1.892857143	0.141822978	0.102244376
151301	F1816	L	F	4	LH	1.38996139	0.136462913	0.101831841
151301	F1816	L	F	4	RH	1.967592593	0.110901966	0.080393742
151301	F1816	L	F	6	LF	1.675824176	0.104356421	0.073160969
151301	F1816	L	F	6	RF	1.535682023	0.096654259	0.074242384
151301	F1816	L	F	6	LH	1.517857143	0.08315607	0.060913434
151301	F1816	L	F	6	RH	1.683501684	0.103919125	0.063092876
151301	F1816	L	F	8	LF	1.403985507	0.095453012	0.069950269
151301	F1816	L	F	8	RF	1.373134328	0.088923641	0.072872585
151301	F1816	L	F	8	LH	1.283068783	0.091445148	0.077532883
151301	F1816	L	F	8	RH	1.547619048	0.062830291	0.036877206
151301	F1816	L	F	24	LF	1.568627451	0.108981558	0.076944875
151301	F1816	L	F	24	RF	1.449579832	0.11140438	0.085354816
151301	F1816	L	F	24	LH	1.422275641	0.103364274	0.078426007
151301	F1816	L	F	24	RH	1.509009009	0.112730373	0.070097462
151301	F1816	L	F	26	LF	1.96969697	0.123620984	0.093160459
151301	F1816	L	F	26	RF	1.509009009	0.121012062	0.0934975
151301	F1816	L	F	26	LH	1.41620771	0.117951404	0.091431875
151301	F1816	L	F	26	RH	1.699308756	0.12421103	0.080965275
151301	F1816	L	F	28	LF	2.086956522	0.158966192	0.101544531

151301	F1816	L	F	28	RF	2	0.155490544	0.111765045
151301	F1816	L	F	28	LH	1.854395604	0.160373444	0.10475921
151301	F1816	L	F	28	RH	2	0.1633685	0.106857768
151301	F1816	L	F	96	LF	1.640625	0.157069054	0.092280221
151301	F1816	L	F	96	RF	1.93236715	0.180014464	0.080855431
151301	F1816	L	F	96	LH	2.220394737	0.179251959	0.081617935
151301	F1816	L	F	96	RH	1.696428571	0.177662344	0.079266153
151302	F1816	L	F	0	LF	1.212121212	0.092605758	0.08136251
151302	F1816	L	F	0	RF	1.06029106	0.093048567	0.071900702
151302	F1816	L	F	0	LH	1.191066998	0.072180863	0.044895577
151302	F1816	L	F	0	RH	1.582792208	0.082945371	0.060299365
151302	F1816	L	F	1	LF	0.928689884	0.072815192	0.056498581
151302	F1816	L	F	1	RF	0.714431517	0.059617818	0.053073978
151302	F1816	L	F	1	LH	0.608016304	0.058880926	0.047861634
151302	F1816	L	F	1	RH	0.86852351	0.066364861	0.041882494
151302	F1816	L	F	2	LF	2	0.090983642	0.074177529
151302	F1816	L	F	2	RF	1.674107143	0.075498343	0.054829152
151302	F1816	L	F	2	LH	1.674107143	0.056131626	0.048203291
151302	F1816	L	F	2	RH	1.892857143	0.076530988	0.048276865
151302	F1816	L	F	4	LF	1.759259259	0.089271713	0.06542175
151302	F1816	L	F	4	RF	1.675675676	0.098959277	0.072882367
151302	F1816	L	F	4	LH	1.503094607	0.093884487	0.077193769
151302	F1816	L	F	4	RH	1.823607427	0.08822926	0.06627953
151302	F1816	L	F	6	LF	1.55651341	0.087904227	0.061966515
151302	F1816	L	F	6	RF	1.535682023	0.079522471	0.063467203
151302	F1816	L	F	6	LH	1.625	0.073146865	0.05575215
151302	F1816	L	F	6	RH	1.5625	0.082489335	0.051094484
151302	F1816	L	F	8	LF	1.482127289	0.0878651	0.06832467
151302	F1816	L	F	8	RF	1.619644723	0.073892412	0.055526048
151302	F1816	L	F	8	LH	1.628151261	0.075399348	0.044407966
151302	F1816	L	F	8	RH	1.822916667	0.069452118	0.053678846
151302	F1816	L	F	24	LF	1.509009009	0.109975352	0.083509395
151302	F1816	L	F	24	RF	1.533882784	0.107802917	0.087421454

151302	F1816	L	F	24	LH	1.509009009	0.105069532	0.067096833
151302	F1816	L	F	24	RH	1.431451613	0.111971206	0.067854299
151302	F1816	L	F	26	LF	1.25	0.084301038	0.066463567
151302	F1816	L	F	26	RF	1.46434635	0.062351158	0.051682036
151302	F1816	L	F	26	LH	1.324041812	0.042507542	0.04786386
151302	F1816	L	F	26	RH	1.41620771	0.07500193	0.050041275
151302	F1816	L	F	28	LF	1.550925926	0.089396346	0.067457715
151302	F1816	L	F	28	RF	1.494107744	0.101413263	0.082148217
151302	F1816	L	F	28	LH	1.488095238	0.109505071	0.071126137
151302	F1816	L	F	28	RH	1.474358974	0.099915884	0.061134692
151302	F1816	L	F	96	LF	0.961538462	0.128912216	0.086165089
151302	F1816	L	F	96	RF	0.897435897	0.122291431	0.104282298
151302	F1816	L	F	96	LH	0.883995037	0.132518144	0.096444716
151302	F1816	L	F	96	RH	0.916666667	0.13050977	0.086086815
151306	F1349	L	F	4	LF	1.823607427	0.074720954	0.053022822
151306	F1349	L	F	4	RF	1.587701613	0.079576704	0.054699109
151306	F1349	L	F	4	LH	1.754926108	0.074422835	0.059636212
151306	F1349	L	F	4	RH	1.903735632	0.063690705	0.049190924
151306	F1349	L	F	6	LF	1.241666667	0.089369641	0.073093523
151306	F1349	L	F	6	RF	1.125925926	0.108777995	0.087215722
151306	F1349	L	F	6	LH	1.300631735	0.09677675	0.038290819
151306	F1349	L	F	6	RH	1.353874883	0.088391689	0.045665367
151309	F943	L	F	1	LF	0.694444444	0.064713265	0.030989985
151309	F943	L	F	1	RF	0.735294118	0.079185033	0.051116555
151309	F943	L	F	1	LH	1.041666667	0.037499927	0.043179943
151309	F943	L	F	1	RH	0.568181818	0.060219147	0.039459559
151309	F943	L	F	2	LF	0.681818182	0.133639675	0.090289269
151309	F943	L	F	2	RF	0.748938073	0.140506726	0.078465486
151309	F943	L	F	2	LH	0.645833333	0.162708806	0.108784115
151309	F943	L	F	2	RH	0.828033916	0.104904511	0.074639223
151309	F943	L	F	4	LF	1.5625	0.102073245	0.083289102
151309	F943	L	F	4	RF	1.231231231	0.110265906	0.073435382
151309	F943	L	F	4	LH	1.338971106	0.102776043	0.061841341

151309	F943	L	F	4	RH	1.285431773	0.115773921	0.070310145
151309	F943	L	F	6	LF	1.586538462	0.114342103	0.085318233
151309	F943	L	F	6	RF	1.213450292	0.12236183	0.081172238
151309	F943	L	F	6	LH	1.401515152	0.107566054	0.063541613
151309	F943	L	F	6	RH	1.543478261	0.112665866	0.073104194
151309	F943	L	F	8	LF	1.50261324	0.093156145	0.070985398
151309	F943	L	F	8	RF	1.148989899	0.117122475	0.086750004
151309	F943	L	F	8	LH	1.147373789	0.113567557	0.077399878
151309	F943	L	F	8	RH	1.239290086	0.108080657	0.082391333
151309	F943	L	F	24	LF	1.09159943	0.072730331	0.057008974
151309	F943	L	F	24	RF	0.934103261	0.096006668	0.073211585
151309	F943	L	F	24	LH	1.475694444	0.061018617	0.055345848
151309	F943	L	F	24	RH	0.881316099	0.097123173	0.060035893
151309	F943	L	F	26	LF	1.278409091	0.137301843	0.099404307
151309	F943	L	F	26	RF	1.487068966	0.125491227	0.093237794
151309	F943	L	F	26	LH	1.533882784	0.124691677	0.077399546
151309	F943	L	F	26	RH	1.338971106	0.137389969	0.089326079
151309	F943	L	F	28	LF	0.965391621	0.097689095	0.080071963
151309	F943	L	F	28	RF	0.80624355	0.106837566	0.083765074
151309	F943	L	F	28	LH	0.822192513	0.098933112	0.075660105
151309	F943	L	F	28	RH	0.960183228	0.099643292	0.072969008
151310	F943	L	F	0	LF	0.78125	0.075326895	0.052401318
151310	F943	L	F	0	RF	1.351351351	0.052428779	0.030321973
151310	F943	L	F	0	LH	2.173913043	0.016375412	0.011259982
151310	F943	L	F	0	RH	0.694444444	0.106440178	0.051786551
151310	F943	L	F	1	LF	1.431451613	0.070322982	0.055371475
151310	F943	L	F	1	RF	1.160714286	0.063705912	0.042748616
151310	F943	L	F	1	LH	2.401960784	0.042039242	0.027518954
151310	F943	L	F	1	RH	3.541666667	0.022876657	0.015663446
151310	F943	L	F	2	LF	0.906070674	0.070607251	0.058795736
151310	F943	L	F	2	RF	0.764167782	0.079992822	0.055706438
151310	F943	L	F	2	LH	0.826032541	0.082219465	0.054452454
151310	F943	L	F	2	RH	1.445005612	0.055599412	0.034643468

151310	F943	L	F	4	LF	1.794871795	0.112881761	0.085485305
151310	F943	L	F	4	RF	2.315789474	0.085349128	0.066570635
151310	F943	L	F	4	LH	1.967592593	0.093098167	0.067702249
151310	F943	L	F	4	RH	1.695402299	0.117639285	0.069919934
151310	F943	L	F	6	LF	1.278409091	0.106760742	0.079645877
151310	F943	L	F	6	RF	1.316689466	0.106255041	0.083776739
151310	F943	L	F	6	LH	1.349431818	0.103779593	0.068636985
151310	F943	L	F	6	RH	1.303475936	0.108374716	0.063160079
151310	F943	L	F	8	LF	0.994371482	0.086315286	0.064887264
151310	F943	L	F	8	RF	1.226076555	0.029787562	0.022573646
151310	F943	L	F	8	LH	1.387846962	0.036595991	0.019463415
151310	F943	L	F	8	RH	0.933508025	0.098819283	0.060589373
151310	F943	L	F	24	LF	1.213450292	0.112134935	0.095020553
151310	F943	L	F	24	RF	1.18980963	0.109137996	0.086328909
151310	F943	L	F	24	LH	1.157894737	0.118144207	0.089385168
151310	F943	L	F	24	RH	1.156914894	0.108007114	0.065875091
151310	F943	L	F	26	LF	1.153234358	0.120699009	0.094832861
151310	F943	L	F	26	RF	1.058201058	0.118246966	0.093437841
151310	F943	L	F	26	LH	1.035940803	0.120156781	0.087897244
151310	F943	L	F	26	RH	1.089015152	0.120274267	0.092293764
151310	F943	L	F	28	LF	1.163419913	0.148801754	0.111312054
151310	F943	L	F	28	RF	1.362645349	0.144166463	0.107252872
151310	F943	L	F	28	LH	1.38996139	0.147486196	0.113868594
151310	F943	L	F	28	RH	1.209207459	0.150240116	0.10077859
151310	F943	L	F	96	LF	2.170138889	0.099556619	0.058097429
151310	F943	L	F	96	RF	1.940789474	0.120688051	0.078581473
151310	F943	L	F	96	LH	1.81547619	0.126287277	0.083520447
151310	F943	L	F	96	RH	2.34375	0.096306359	0.057172931
152686	F1158	L	F	1	LF	0.746268657	0.039097412	0.027023162
152686	F1158	L	F	1	RF	0.847457627	0.035467855	0.033369742
152686	F1158	L	F	1	LH	4.166666667	-0.000928505	-0.000400129
152686	F1158	L	F	1	RH	0.833333333	0.029646689	0.026934865
152686	F1158	L	F	2	LF	1.338366507	0.085228988	0.071504717

152686	F1158	L	F	2	RF	1.471825063	0.082996922	0.05176921
152686	F1158	L	F	2	LH	1.401515152	0.083655648	0.069756451
152686	F1158	L	F	2	RH	1.345050215	0.080497703	0.060842045
152686	F1158	L	F	4	LF	1.266025641	0.146801002	0.115280265
152686	F1158	L	F	4	RF	1.422275641	0.140857799	0.093260358
152686	F1158	L	F	4	LH	1.239290086	0.147455809	0.102031327
152686	F1158	L	F	4	RH	1.339285714	0.136248613	0.097766507
152686	F1158	L	F	6	LF	1.360294118	0.131753303	0.084958623
152686	F1158	L	F	6	RF	1.439144737	0.145003388	0.0869871
152686	F1158	L	F	6	LH	1.495535714	0.153618514	0.091388231
152686	F1158	L	F	6	RH	1.726190476	0.138554813	0.09966572
152686	F1158	L	F	8	LF	1.431451613	0.152114966	0.108034616
152686	F1158	L	F	8	RF	1.49122807	0.163947425	0.112893211
152686	F1158	L	F	8	LH	1.456925676	0.167914922	0.105819422
152686	F1158	L	F	8	RH	1.410969793	0.159730097	0.103258101
152686	F1158	L	F	24	LF	0.848432557	0.148481041	0.116782317
152686	F1158	L	F	24	RF	0.890605296	0.171423419	0.128919832
152686	F1158	L	F	24	LH	1.001602564	0.158682811	0.117873078
152686	F1158	L	F	24	RH	0.952467344	0.142917765	0.099727111
152686	F1158	L	F	26	LF	1.609756098	0.126282818	0.08913473
152686	F1158	L	F	26	RF	1.487068966	0.150713355	0.101018656
152686	F1158	L	F	26	LH	1.568532819	0.147822828	0.096298692
152686	F1158	L	F	26	RH	1.682692308	0.130911067	0.08059778
152686	F1158	L	F	28	LF	0.956632653	0.150094046	0.123618399
152686	F1158	L	F	28	RF	0.962949376	0.148802584	0.126758464
152686	F1158	L	F	28	LH	0.881534179	0.160561089	0.125861674
152686	F1158	L	F	28	RH	1	0.142370834	0.101088983
154983	F1571	L	F	4	LF	1.942815249	0.128773761	0.089297645
154983	F1571	L	F	4	RF	1.424772036	0.173940308	0.110449014
154983	F1571	L	F	4	LH	1.707175926	0.131083524	0.111001455
154983	F1571	L	F	4	RH	2.661064426	0.09158952	0.049152424
154983	F1571	L	F	6	LF	1.568532819	0.141333908	0.10223123
154983	F1571	L	F	6	RF	1.393188854	0.092894392	0.074171561

154983	F1571	L	F	6	LH	1.859756098	0.082396355	0.045748387
154983	F1571	L	F	6	RH	1.382575758	0.155598822	0.085737726
154983	F1571	L	F	8	LF	1.156914894	0.136741697	0.10146361
154983	F1571	L	F	8	RF	2.073365231	0.093566984	0.060261511
154983	F1571	L	F	8	LH	1.717342342	0.114998371	0.067037766
154983	F1571	L	F	8	RH	1.131221719	0.146220144	0.108244928
159811	F1541	L	M	1	LF	1.058201058	0.074393116	0.060864402
159811	F1541	L	M	1	RF	1.06029106	0.062802701	0.043091959
159811	F1541	L	M	1	LH	0.968372424	0.08409716	0.052731044
159811	F1541	L	M	1	RH	1.196509009	0.066488862	0.051345189
159811	F1541	L	M	2	LF	1.303475936	0.109499727	0.072818852
159811	F1541	L	M	2	RF	1.257071025	0.119294482	0.092865746
159811	F1541	L	M	2	LH	1.313131313	0.108936203	0.075562909
159811	F1541	L	M	2	RH	1.761363636	0.094906197	0.073974053
159811	F1541	L	M	4	LF	1.521164021	0.08399683	0.070822954
159811	F1541	L	M	4	RF	1.550925926	0.091088196	0.069613068
159811	F1541	L	M	4	LH	1.533882784	0.093018944	0.068018056
159811	F1541	L	M	4	RH	1.597222222	0.08881344	0.064052891
159811	F1541	L	M	6	LF	1.138716356	0.108050125	0.091884852
159811	F1541	L	M	6	RF	0.885025063	0.094206949	0.090050894
159811	F1541	L	M	6	LH	1.136950904	0.077568132	0.065564388
159811	F1541	L	M	6	RH	1.257763975	0.09417228	0.078007006
159811	F1541	L	M	8	LF	1.207590569	0.148607278	0.107859245
159811	F1541	L	M	8	RF	1.289490651	0.122589971	0.098684226
159811	F1541	L	M	8	LH	1.316701317	0.116594268	0.092712866
159811	F1541	L	M	8	RH	1.17872807	0.152379822	0.091795198
159811	F1541	L	M	24	LF	1.470588235	0.14982069	0.121901154
159811	F1541	L	M	24	RF	1.352339181	0.126930781	0.09577627
159811	F1541	L	M	24	LH	1.313164894	0.118106414	0.091311011
159811	F1541	L	M	24	RH	1.428571429	0.140516274	0.093084042
159811	F1541	L	M	26	LF	1.063596491	0.128549816	0.102195211
159811	F1541	L	M	26	RF	1.148090815	0.126460672	0.097212141
159811	F1541	L	M	26	LH	1.172940535	0.131105266	0.091363934

159811	F1541	L	M	26	RH	1.026422764	0.136850618	0.097433704
159811	F1541	L	M	28	LF	1.537744641	0.171207014	0.127944448
159811	F1541	L	M	28	RF	1.482127289	0.152767625	0.119144282
159811	F1541	L	M	28	LH	1.289490651	0.156253126	0.1212573
159811	F1541	L	M	28	RH	1.456925676	0.174360246	0.10016077
159811	F1541	L	M	96	LF	1.30952381	0.192818684	0.14462633
159811	F1541	L	M	96	RF	1.408730159	0.185897956	0.13256045
159811	F1541	L	M	96	LH	1.495535714	0.183617307	0.107561887
159811	F1541	L	M	96	RH	1.429738562	0.184053036	0.12282581
159828	F1546	L	F	0	LF	1.707175926	0.100938712	0.077075877
159828	F1546	L	F	0	RF	1.075393154	0.07181313	0.046042667
159828	F1546	L	F	0	LH	1.46969697	0.107469201	0.098615727
159828	F1546	L	F	0	RH	1.349431818	0.073456505	0.065430927
159828	F1546	L	F	1	LF	1.787994891	0.1459536	0.085354851
159828	F1546	L	F	1	RF	1.823607427	0.148501317	0.103460015
159828	F1546	L	F	1	LH	2.277039848	0.123183348	0.082342209
159828	F1546	L	F	1	RH	1.823607427	0.141030086	0.081514812
159828	F1546	L	F	2	LF	1.498682477	0.101629922	0.07279596
159828	F1546	L	F	2	RF	1.552795031	0.141324046	0.077554313
159828	F1546	L	F	2	LH	1.831395349	0.075328471	0.047743257
159828	F1546	L	F	2	RH	1.523809524	0.128835898	0.082756635
159828	F1546	L	F	4	LF	1.331453634	0.13576955	0.089597674
159828	F1546	L	F	4	RF	1.949025487	0.124085003	0.064461399
159828	F1546	L	F	4	LH	3.362068966	0.084500486	0.055197697
159828	F1546	L	F	4	RH	1.252948113	0.142649717	0.088945358
159828	F1546	L	F	6	LF	1.590909091	0.093488781	0.079985809
159828	F1546	L	F	6	RF	1.372180451	0.112556533	0.076807863
159828	F1546	L	F	6	LH	1.767990074	0.085630626	0.05724066
159828	F1546	L	F	6	RH	1.996927803	0.078398042	0.05677433
159828	F1546	L	F	8	LF	1.668520578	0.157617625	0.111858059
159828	F1546	L	F	8	RF	1.726190476	0.168374512	0.123052606
159828	F1546	L	F	8	LH	1.590909091	0.176700089	0.113336107
159828	F1546	L	F	8	RH	1.726190476	0.168452851	0.102766753

159828	F1546	L	F	24	LF	1.668520578	0.142106102	0.102514241
159828	F1546	L	F	24	RF	1.668520578	0.164342567	0.116803719
159828	F1546	L	F	24	LH	1.5625	0.177913836	0.114196844
159828	F1546	L	F	24	RH	1.699308756	0.150387862	0.089474861
159828	F1546	L	F	26	LF	1.428571429	0.166539774	0.111166172
159828	F1546	L	F	26	RF	1.49122807	0.183441131	0.137456754
159828	F1546	L	F	26	LH	1.547619048	0.17794889	0.122939267
159828	F1546	L	F	26	RH	1.482127289	0.174856841	0.115573942
159828	F1546	L	F	28	LF	2.097902098	0.187052733	0.129625959
159828	F1546	L	F	28	RF	2.041666667	0.201430844	0.134573012
159828	F1546	L	F	28	LH	2.232142857	0.184688214	0.104006796
159828	F1546	L	F	28	RH	2.097902098	0.193294161	0.114523178
159828	F1546	L	F	96	LF	2.142857143	0.220313727	0.136913126
159828	F1546	L	F	96	RF	2.012882448	0.225320199	0.155282089
159828	F1546	L	F	96	LH	2.012882448	0.220558113	0.124870494
159828	F1546	L	F	96	RH	2.211538462	0.218408378	0.128556726
160446	F1546	L	M	0	LF	1.044358312	0.0829112	0.048505462
160446	F1546	L	M	0	RF	1.257071025	0.07569219	0.074263777
160446	F1546	L	M	0	LH	1.382575758	0.077420624	0.079912767
160446	F1546	L	M	0	RH	0.984621155	0.092143619	0.06741496
160446	F1546	L	M	1	LF	1.06216458	0.094813944	0.074592238
160446	F1546	L	M	1	RF	1.147373789	0.088532404	0.072665071
160446	F1546	L	M	1	LH	1.048728814	0.099157886	0.085768532
160446	F1546	L	M	1	RH	1.053093462	0.097326811	0.073457714
160446	F1546	L	M	2	LF	1.527777778	0.125022583	0.102840335
160446	F1546	L	M	2	RF	1.415470494	0.123301792	0.094189962
160446	F1546	L	M	2	LH	1.393188854	0.126222071	0.080817834
160446	F1546	L	M	2	RH	1.538825758	0.116932759	0.088132056
160446	F1546	L	M	4	LF	1.376811594	0.094346303	0.081721578
160446	F1546	L	M	4	RF	1.257763975	0.096252914	0.08298245
160446	F1546	L	M	4	LH	1.168478261	0.102356992	0.082921905
160446	F1546	L	M	4	RH	1.302083333	0.097952308	0.071605771
160446	F1546	L	M	6	LF	1.253132832	0.131269171	0.102478151

160446	F1546	L	M	6	RF	1.351351351	0.133617206	0.109763344
160446	F1546	L	M	6	LH	1.335470085	0.135158455	0.101704774
160446	F1546	L	M	6	RH	1.204994193	0.144918415	0.098022372
160446	F1546	L	M	8	LF	1.339285714	0.119157425	0.106285923
160446	F1546	L	M	8	RF	1.250781739	0.120621072	0.102031117
160446	F1546	L	M	8	LH	1.219512195	0.116847291	0.084676652
160446	F1546	L	M	8	RH	1.165311653	0.133465403	0.086099467
160446	F1546	L	M	24	LF	1.243857494	0.113284099	0.093173744
160446	F1546	L	M	24	RF	1.185879757	0.113881011	0.097897474
160446	F1546	L	M	24	LH	1.18980963	0.116593142	0.088323885
160446	F1546	L	M	24	RH	1.145833333	0.118784862	0.092433515
160446	F1546	L	M	26	LF	1.401689708	0.119416458	0.093098312
160446	F1546	L	M	26	RF	1.324728261	0.138661246	0.102070281
160446	F1546	L	M	26	LH	1.387846962	0.128713996	0.098626125
160446	F1546	L	M	26	RH	1.352813853	0.129896654	0.083622818
160446	F1546	L	M	28	LF	1.285431773	0.121792462	0.100139001
160446	F1546	L	M	28	RF	1.298920378	0.118923161	0.091529296
160446	F1546	L	M	28	LH	1.25	0.124007244	0.097068791
160446	F1546	L	M	28	RH	1.266025641	0.125059493	0.097400806
160446	F1546	L	M	96	LF	1.111660079	0.134451357	0.117420676
160446	F1546	L	M	96	RF	1	0.122056085	0.104547492
160446	F1546	L	M	96	LH	1.031037415	0.126109845	0.097218763
160446	F1546	L	M	96	RH	1.087470449	0.121969439	0.093280198
160639	F1546	L	F	0	LF	0.694252556	0.065103164	0.047848802
160639	F1546	L	F	0	RF	0.568058274	0.100506467	0.079600325
160639	F1546	L	F	0	LH	1.175213675	0.057956024	0.048993533
160639	F1546	L	F	0	RH	0.59247106	0.086326342	0.078671632
160639	F1546	L	F	1	LF	1.533385093	0.093748807	0.063507978
160639	F1546	L	F	1	RF	2.103174603	0.081627483	0.049378532
160639	F1546	L	F	1	LH	2.02991453	0.086806314	0.060077468
160639	F1546	L	F	1	RH	1.5771526	0.092439683	0.067660941
160639	F1546	L	F	2	LF	1.537744641	0.101700665	0.081839229
160639	F1546	L	F	2	RF	1.229273871	0.092616629	0.072631946

160639	F1546	L	F	2	LH	1.00308642	0.146735333	0.093147413
160639	F1546	L	F	2	RH	1.008403361	0.118775447	0.088016418
160639	F1546	L	F	4	LF	1.391006098	0.121525067	0.092240091
160639	F1546	L	F	4	RF	2.056451613	0.094865546	0.062712038
160639	F1546	L	F	4	LH	1.500896057	0.160655459	0.106321528
160639	F1546	L	F	4	RH	1.345050215	0.138276249	0.087233227
160639	F1546	L	F	6	LF	1.887464387	0.137621859	0.097635285
160639	F1546	L	F	6	RF	1.823607427	0.154918454	0.100964486
160639	F1546	L	F	6	LH	1.757575758	0.154961834	0.113716675
160639	F1546	L	F	6	RH	1.822250639	0.149798886	0.086985861
160639	F1546	L	F	8	LF	2.152014652	0.160733129	0.113442482
160639	F1546	L	F	8	RF	1.818783069	0.16648417	0.11345768
160639	F1546	L	F	8	LH	1.862068966	0.16666904	0.098411473
160639	F1546	L	F	8	RH	2.012882448	0.163462693	0.10491757
160639	F1546	L	F	24	LF	1.119960179	0.149203812	0.103360906
160639	F1546	L	F	24	RF	1.234756098	0.147343694	0.114954916
160639	F1546	L	F	24	LH	1.316701317	0.130685154	0.095291802
160639	F1546	L	F	24	RH	1.219153937	0.140634917	0.096906709
160639	F1546	L	F	26	LF	1.439144737	0.151429264	0.107165104
160639	F1546	L	F	26	RF	1.304200542	0.159004298	0.121120393
160639	F1546	L	F	26	LH	1.352813853	0.138591934	0.111621862
160639	F1546	L	F	26	RH	1.487068966	0.149994581	0.095095158
160639	F1546	L	F	28	LF	1.372180451	0.136594787	0.10125299
160639	F1546	L	F	28	RF	1.568627451	0.146875	0.112451172
160639	F1546	L	F	28	LH	1.492869875	0.155944901	0.089501953
160639	F1546	L	F	28	RH	1.355311355	0.146416015	0.104187535
160639	F1546	L	F	96	LF	1.657894737	0.168382736	0.12602911
160639	F1546	L	F	96	RF	0.974350433	0.170695581	0.128599817
160639	F1546	L	F	96	LH	0.970394737	0.156613423	0.12006871
160639	F1546	L	F	96	RH	1.55075188	0.173867441	0.112989934
151303	F943	N	F	0	LF	0.965700966	0.04534333	0.03738045
151303	F943	N	F	0	RF	0.813061872	0.056976035	0.051846403
151303	F943	N	F	0	LH	1.940789474	0.023274961	0.018811279

151303	F943	N	F	0	RH	0.757062147	0.055323552	0.049307478
151303	F943	N	F	1	LF	1.163419913	0.097976936	0.073068823
151303	F943	N	F	1	RF	1.41620771	0.074622706	0.062042995
151303	F943	N	F	1	LH	1.168478261	0.092943284	0.068560402
151303	F943	N	F	1	RH	2.306547619	0.083289705	0.052020125
151303	F943	N	F	2	LF	1.637214137	0.133288183	0.102009201
151303	F943	N	F	2	RF	1.833333333	0.110136615	0.074493535
151303	F943	N	F	2	LH	1.961538462	0.09931019	0.069417278
151303	F943	N	F	2	RH	1.996927803	0.104588072	0.076582437
151303	F943	N	F	4	LF	1.382575758	0.110788451	0.087135166
151303	F943	N	F	4	RF	1.719114219	0.104583579	0.078877733
151303	F943	N	F	4	LH	1.655982906	0.108567881	0.077019909
151303	F943	N	F	4	RH	1.614583333	0.105952899	0.066150725
151303	F943	N	F	6	LF	1.316701317	0.100222186	0.080725965
151303	F943	N	F	6	RF	1.136950904	0.097530992	0.068162205
151303	F943	N	F	6	LH	1.243857494	0.085182709	0.072245712
151303	F943	N	F	6	RH	1.298920378	0.092379188	0.076718503
151303	F943	N	F	8	LF	1.587701613	0.120287107	0.085278638
151303	F943	N	F	8	RF	1.614583333	0.115303081	0.083302531
151303	F943	N	F	8	LH	1.538825758	0.121488079	0.089307348
151303	F943	N	F	8	RH	1.452020202	0.133889884	0.087518182
151303	F943	N	F	24	LF	1.474252492	0.101677881	0.084581257
151303	F943	N	F	24	RF	1.319444444	0.101542115	0.081853906
151303	F943	N	F	24	LH	1.282051282	0.109053362	0.070864119
151303	F943	N	F	24	RH	1.196581197	0.112574578	0.088312569
151303	F943	N	F	26	LF	0.974025974	0.114718469	0.086316469
151303	F943	N	F	26	RF	1.13022113	0.102240955	0.085284339
151303	F943	N	F	26	LH	1.012429832	0.081564375	0.081714131
151303	F943	N	F	26	RH	1.050861707	0.109165893	0.090907259
151303	F943	N	F	28	LF	1.214285714	0.12860409	0.103186079
151303	F943	N	F	28	RF	1.330532213	0.113373158	0.102898351
151303	F943	N	F	28	LH	1.355311355	0.115168941	0.084290043
151303	F943	N	F	28	RH	1.239290086	0.121416052	0.100235827

151303	F943	N	F	96	LF	0.907738095	0.140187091	0.10574993
151303	F943	N	F	96	RF	0.924185464	0.132714858	0.099044931
151303	F943	N	F	96	LH	0.820512821	0.163334302	0.089883173
151303	F943	N	F	96	RH	0.880681818	0.137518498	0.101752597
151307	F943	N	F	0	LF	1.188811189	0.074430723	0.066575662
151307	F943	N	F	0	RF	0.840870548	0.090101648	0.084022688
151307	F943	N	F	0	LH	2.455357143	0.025416636	0.024646464
151307	F943	N	F	0	RH	0.495169082	0.11938791	0.092176776
151307	F943	N	F	1	LF	1.235294118	0.145681243	0.103541255
151307	F943	N	F	1	RF	1.416122004	0.13199919	0.110448705
151307	F943	N	F	1	LH	2.566964286	0.075111941	0.055267157
151307	F943	N	F	1	RH	1.153234358	0.14913031	0.105317936
151307	F943	N	F	2	LF	1.619433198	0.10576181	0.080376024
151307	F943	N	F	2	RF	1.683501684	0.118157617	0.088901556
151307	F943	N	F	2	LH	1.503094607	0.13563996	0.099111858
151307	F943	N	F	2	RH	1.474358974	0.117499614	0.071838847
151307	F943	N	F	4	LF	2.062289562	0.161575451	0.112536416
151307	F943	N	F	4	RF	2.056451613	0.179778497	0.125461995
151307	F943	N	F	4	LH	2.178030303	0.164884624	0.099359706
151307	F943	N	F	4	RH	1.903735632	0.186366344	0.115315609
151307	F943	N	F	6	LF	1.742788462	0.140598286	0.105462961
151307	F943	N	F	6	RF	1.887464387	0.112828527	0.088855288
151307	F943	N	F	6	LH	1.851851852	0.112176295	0.07888879
151307	F943	N	F	6	RH	1.726190476	0.138452797	0.108050699
151307	F943	N	F	8	LF	1.612903226	0.14364299	0.119171449
151307	F943	N	F	8	RF	1.851851852	0.106925757	0.089449568
151307	F943	N	F	8	LH	1.785714286	0.092793561	0.071300968
151307	F943	N	F	8	RH	1.428571429	0.155072056	0.119730452
151307	F943	N	F	24	LF	1.227130745	0.17249913	0.138297768
151307	F943	N	F	24	RF	1.166947014	0.151727725	0.111448651
151307	F943	N	F	24	LH	1.697530864	0.119197502	0.070794522
151307	F943	N	F	24	RH	1.697530864	0.136960025	0.106547405
151307	F943	N	F	26	LF	1.541745731	0.150663451	0.115840557

151307	F943	N	F	26	RF	1.449579832	0.148095861	0.114289981
151307	F943	N	F	26	LH	1.355311355	0.137837267	0.103518547
151307	F943	N	F	26	RH	1.452020202	0.161169251	0.108528774
151307	F943	N	F	28	LF	1.566951567	0.133001085	0.102487874
151307	F943	N	F	28	RF	1.519963702	0.131162097	0.113213588
151307	F943	N	F	28	LH	1.433251433	0.132761857	0.105361744
151307	F943	N	F	28	RH	1.555555556	0.142478939	0.106522887
151307	F943	N	F	96	LF	1.056034483	0.154003339	0.122187429
151307	F943	N	F	96	RF	0.967261905	0.155390341	0.125757498
151307	F943	N	F	96	LH	0.999216301	0.145939215	0.095571384
151307	F943	N	F	96	RH	1.041666667	0.146094385	0.091552989
152750	F1571	N	F	0	LF	1.229273871	0.037717264	0.014741865
152750	F1571	N	F	0	RF	2.592165899	0.028552461	0.021807068
152750	F1571	N	F	0	LH	0.57294395	0.092545326	0.07869848
152750	F1571	N	F	0	RH	0.67299396	0.103365083	0.066277542
152750	F1571	N	F	1	LF	0.983102919	0.130247097	0.110602042
152750	F1571	N	F	1	RF	3.720238095	0.075244424	0.049298093
152750	F1571	N	F	1	LH	0.938482704	0.102968806	0.079246438
152750	F1571	N	F	1	RH	0.542174956	0.180141375	0.135006246
152750	F1571	N	F	2	LF	1.471861472	0.161707563	0.120669175
152750	F1571	N	F	2	RF	1.387846962	0.136690568	0.115915186
152750	F1571	N	F	2	LH	1.66827853	0.110242297	0.086572581
152750	F1571	N	F	2	RH	1.607142857	0.140044898	0.107630958
152750	F1571	N	F	4	LF	1.324728261	0.148506553	0.111740783
152750	F1571	N	F	4	RF	0.978017884	0.134348802	0.119201347
152750	F1571	N	F	4	LH	1.396825397	0.109489937	0.094941234
152750	F1571	N	F	4	RH	1.247771836	0.167966917	0.129857327
152750	F1571	N	F	6	LF	1.515151515	0.135136269	0.111298988
152750	F1571	N	F	6	RF	0.641025641	0.144254681	0.11893539
152750	F1571	N	F	6	LH	4.166666667	0.062309254	0.022694396
152750	F1571	N	F	6	RH	1.5625	0.13319158	0.100088154
152750	F1571	N	F	8	LF	1.731601732	0.156884485	0.12426929
152750	F1571	N	F	8	RF	1.393983859	0.193546044	0.133401224

152750	F1571	N	F	8	LH	1.531914894	0.188932266	0.123646904
152750	F1571	N	F	8	RH	1.471825063	0.186207863	0.123364411
152750	F1571	N	F	24	LF	0.934343434	0.165316695	0.133018437
152750	F1571	N	F	24	RF	0.874380647	0.187431447	0.134913061
152750	F1571	N	F	24	LH	0.962837838	0.188932266	0.126175866
152750	F1571	N	F	24	RH	0.92405914	0.163936788	0.130736621
152750	F1571	N	F	26	LF	1.313131313	0.229632115	0.182098151
152750	F1571	N	F	26	RF	1.216063348	0.243750576	0.176790631
152750	F1571	N	F	26	LH	1.373626374	0.198950745	0.13251333
152750	F1571	N	F	26	RH	1.296647691	0.236680787	0.170196623
152750	F1571	N	F	28	LF	1.892857143	0.224480389	0.163748075
152750	F1571	N	F	28	RF	1.674107143	0.186077913	0.148396919
152750	F1571	N	F	28	LH	1.431451613	0.178801993	0.135087442
152750	F1571	N	F	28	RH	1.93452381	0.193987612	0.128611014
152750	F1571	N	F	96	LF	1.367331855	0.22598663	0.171018657
152750	F1571	N	F	96	RF	1.387846962	0.192825228	0.163348771
152750	F1571	N	F	96	LH	1.367331855	0.159434652	0.120389321
152750	F1571	N	F	96	RH	1.398601399	0.218854323	0.16048351
152776	F1571	N	M	0	LF	1.438596491	0.095254457	0.074119543
152776	F1571	N	M	0	RF	1.12412178	0.122495108	0.080700172
152776	F1571	N	M	0	LH	1.831501832	0.073512838	0.06416401
152776	F1571	N	M	0	RH	2.083333333	0.062940833	0.028410311
152776	F1571	N	M	1	LF	1.799242424	0.170575924	0.106152374
152776	F1571	N	M	1	RF	1.868206522	0.151541763	0.117865798
152776	F1571	N	M	1	LH	1.850649351	0.15739842	0.111277038
152776	F1571	N	M	1	RH	1.64021164	0.191806432	0.124572845
152776	F1571	N	M	2	LF	1.699308756	0.145796141	0.107849247
152776	F1571	N	M	2	RF	1.213369963	0.171776001	0.144775198
152776	F1571	N	M	2	LH	1.286213786	0.173051945	0.128693126
152776	F1571	N	M	2	RH	1.078088578	0.207684718	0.141996094
152776	F1571	N	M	4	LF	0.785818713	0.126914452	0.116185549
152776	F1571	N	M	4	RF	0.923728814	0.090656452	0.073009066
152776	F1571	N	M	4	LH	1.601601602	0.093372282	0.037431826

152776	F1571	N	M	4	RH	1.102228682	0.111508232	0.094149962
152776	F1571	N	M	6	LF	1.235294118	0.181489636	0.146559543
152776	F1571	N	M	6	RF	1.168831169	0.155200886	0.121859302
152776	F1571	N	M	6	LH	1.130952381	0.165919681	0.124639293
152776	F1571	N	M	6	RH	1.495535714	0.130892875	0.096757002
152776	F1571	N	M	8	LF	0.892857143	0.154150893	0.130051136
152776	F1571	N	M	8	RF	0.853903559	0.155728739	0.118814284
152776	F1571	N	M	8	LH	0.906862745	0.160905598	0.130238074
152776	F1571	N	M	8	RH	0.856264637	0.160507988	0.115103762
152776	F1571	N	M	24	LF	1.430250784	0.178768477	0.144340633
152776	F1571	N	M	24	RF	0.901056015	0.19328016	0.160502222
152776	F1571	N	M	24	LH	0.878927708	0.188791044	0.134772073
152776	F1571	N	M	24	RH	1.302083333	0.188524473	0.145127584
152776	F1571	N	M	26	LF	1.967592593	0.209627164	0.152458891
152776	F1571	N	M	26	RF	1.707175926	0.219221523	0.17116822
152776	F1571	N	M	26	LH	1.614583333	0.222929949	0.151268125
152776	F1571	N	M	26	RH	1.875	0.214012561	0.155178572
152776	F1571	N	M	28	LF	1.735294118	0.235507284	0.169218025
152776	F1571	N	M	28	RF	1.757575758	0.246115442	0.185886942
152776	F1571	N	M	28	LH	1.719114219	0.251104438	0.158452642
152776	F1571	N	M	28	RH	1.607142857	0.265416273	0.16628852
152776	F1571	N	M	96	LF	0.88899196	0.204237817	0.158533309
152776	F1571	N	M	96	RF	0.978220746	0.217400909	0.182813574
152776	F1571	N	M	96	LH	0.959429825	0.208757823	0.151210713
152776	F1571	N	M	96	RH	1.006581494	0.200574925	0.142826436
154850	F998	N	F	0	LF	1.10479798	0.055631529	0.042996421
154850	F998	N	F	0	RF	1.517857143	0.071982907	0.035287833
154850	F998	N	F	0	LH	1.363636364	0.08611184	0.061336025
154850	F998	N	F	0	RH	2.619047619	0.068397377	0.041696327
154850	F998	N	F	1	LF	1.923076923	0.112341935	0.092382821
154850	F998	N	F	1	RF	1.402321083	0.070867845	0.049488147
154850	F998	N	F	1	LH	2.136363636	0.087314986	0.070860197
154850	F998	N	F	1	RH	1.818783069	0.114121769	0.069028446

154850	F998	N	F	2	LF	1.550925926	0.1277035	0.100978832
154850	F998	N	F	2	RF	1.758658009	0.12901564	0.104588658
154850	F998	N	F	2	LH	1.925675676	0.119800237	0.09348087
154850	F998	N	F	2	RH	1.443464314	0.157927987	0.115643346
154850	F998	N	F	4	LF	1.443464314	0.168747816	0.117392109
154850	F998	N	F	4	RF	1.373626374	0.140874293	0.117550433
154850	F998	N	F	4	LH	1.147373789	0.160655608	0.129474754
154850	F998	N	F	4	RH	1.352813853	0.132248988	0.109575164
154850	F998	N	F	6	LF	1.154401154	0.232703896	0.119619917
154850	F998	N	F	6	RF	1.516544118	0.167687498	0.118024689
154850	F998	N	F	6	LH	1.500896057	0.156891356	0.110969278
154850	F998	N	F	6	RH	1.456925676	0.166866658	0.122651577
154850	F998	N	F	8	LF	1.920803783	0.098675552	0.080704127
154850	F998	N	F	8	RF	1.031037415	0.158380639	0.120066849
154850	F998	N	F	8	LH	1.076388889	0.146113411	0.106106115
154850	F998	N	F	8	RH	1.063829787	0.147218453	0.117321645
154850	F998	N	F	24	LF	1.732377539	0.163110252	0.119429119
154850	F998	N	F	24	RF	1.547619048	0.163505379	0.118272721
154850	F998	N	F	24	LH	1.547619048	0.166209078	0.100315569
154850	F998	N	F	24	RH	1.568627451	0.174476544	0.124192805
154850	F998	N	F	26	LF	1.64021164	0.158540749	0.123434687
154850	F998	N	F	26	RF	1.471861472	0.16309409	0.12444007
154850	F998	N	F	26	LH	1.410969793	0.161189892	0.106931469
154850	F998	N	F	26	RH	1.449579832	0.16922848	0.123195907
154850	F998	N	F	28	LF	1.755952381	0.236287014	0.159448594
154850	F998	N	F	28	RF	1.755952381	0.209781085	0.144732687
154850	F998	N	F	28	LH	1.776960784	0.202056588	0.124405062
154850	F998	N	F	28	RH	1.757575758	0.2284825	0.167093073
154850	F998	N	F	96	LF	1.541745731	0.203600652	0.158315262
154850	F998	N	F	96	RF	1.495535714	0.203600652	0.158708921
154850	F998	N	F	96	LH	1.482127289	0.179899731	0.135348491
154850	F998	N	F	96	RH	1.56402737	0.214113178	0.135085894
155005	F1158	N	M	0	LF	0.865800866	0.06190753	0.057536764

155005	F1158	N	M	0	RF	1.956815115	0.048115515	0.024555294
155005	F1158	N	M	0	LH	0.621584699	0.079781354	0.069496808
155005	F1158	N	M	0	RH	1.409836066	0.054724203	0.044870541
155005	F1158	N	M	1	LF	0.973294723	0.095021926	0.074838691
155005	F1158	N	M	1	RF	1.408730159	0.112149216	0.08116893
155005	F1158	N	M	1	LH	1.55075188	0.098796335	0.079168098
155005	F1158	N	M	1	RH	1.023391813	0.093580464	0.072133204
155005	F1158	N	M	2	LF	1.892857143	0.089250943	0.07594621
155005	F1158	N	M	2	RF	1.892857143	0.092251804	0.07574334
155005	F1158	N	M	2	LH	1.37012012	0.091459356	0.077834859
155005	F1158	N	M	2	RH	1.695402299	0.101198211	0.0689841
155005	F1158	N	M	4	LF	1.887464387	0.115750129	0.090297163
155005	F1158	N	M	4	RF	1.862068966	0.138715644	0.097061673
155005	F1158	N	M	4	LH	1.799242424	0.144610293	0.080950465
155005	F1158	N	M	4	RH	1.887464387	0.110557487	0.08182343
155005	F1158	N	M	6	LF	1.628151261	0.127803683	0.098195359
155005	F1158	N	M	6	RF	1.668520578	0.129286433	0.106302887
155005	F1158	N	M	6	LH	1.854395604	0.114707791	0.078599961
155005	F1158	N	M	6	RH	1.619644723	0.13058486	0.096537054
155005	F1158	N	M	8	LF	1.675824176	0.109508137	0.0868828
155005	F1158	N	M	8	RF	2.112068966	0.073382277	0.063026659
155005	F1158	N	M	8	LH	1.509009009	0.122654155	0.095387047
155005	F1158	N	M	8	RH	1.848370927	0.086509588	0.067427055
155005	F1158	N	M	24	LF	1.443464314	0.173417633	0.141217188
155005	F1158	N	M	24	RF	1.503094607	0.196363287	0.141906644
155005	F1158	N	M	24	LH	1.46434635	0.212501626	0.129389745
155005	F1158	N	M	24	RH	1.430250784	0.187086113	0.130616974
155005	F1158	N	M	26	LF	1.699308756	0.214299352	0.163517505
155005	F1158	N	M	26	RF	1.57635468	0.203884423	0.153361031
155005	F1158	N	M	26	LH	1.475694444	0.211661625	0.141812391
155005	F1158	N	M	26	RH	1.587701613	0.213210937	0.168457733
155005	F1158	N	M	28	LF	1.967592593	0.220398709	0.154663255
155005	F1158	N	M	28	RF	1.949025487	0.23621677	0.161190482

155005	F1158	N	M	28	LH	2.211538462	0.217402841	0.149366965
155005	F1158	N	M	28	RH	2.052545156	0.221265277	0.134387147
155005	F1158	N	M	96	LF	1.335470085	0.237037779	0.162937847
155005	F1158	N	M	96	RF	1.538825758	0.194461654	0.15906606
155005	F1158	N	M	96	LH	1.547619048	0.187469302	0.149423822
155005	F1158	N	M	96	RH	1.408730159	0.222299709	0.153112488
155029	F1571	N	F	0	LF	0.328947368	0.261408687	0.154209927
155029	F1571	N	F	0	RF	0.980392157	0.124293533	0.096656196
155029	F1571	N	F	0	LH	0.909090909	0.143693432	0.09418947
155029	F1571	N	F	0	RH	4.545454545	0.005450359	0.0337079
155029	F1571	N	F	1	LF	1.980792317	0.068048566	0.059553591
155029	F1571	N	F	1	RF	2.16503268	0.062886315	0.048013048
155029	F1571	N	F	1	LH	1.090909091	0.100719567	0.084215465
155029	F1571	N	F	1	RH	0.88402757	0.101881052	0.055811234
155029	F1571	N	F	2	LF	1.235119048	0.123517923	0.10697131
155029	F1571	N	F	2	RF	1.333333333	0.103747763	0.095881005
155029	F1571	N	F	2	LH	0.96249379	0.129895432	0.112382955
155029	F1571	N	F	2	RH	1.184640523	0.125889211	0.096357787
155029	F1571	N	F	4	LF	1.119724376	0.156574533	0.130524638
155029	F1571	N	F	4	RF	1.007575758	0.09444419	0.057168514
155029	F1571	N	F	4	LH	1.064241486	0.1514323	0.109374793
155029	F1571	N	F	4	RH	1.05290866	0.140619248	0.096393372
155029	F1571	N	F	8	LF	0.656259708	0.153869475	0.119426478
155029	F1571	N	F	8	RF	0.408399471	0.174498343	0.147334943
155029	F1571	N	F	8	LH	0.713410364	0.163239456	0.136679143
155029	F1571	N	F	8	RH	0.435969368	0.188286489	0.15377796
155029	F1571	N	F	24	LF	1.041666667	0.179539766	0.158866049
155029	F1571	N	F	24	RF	0.970965309	0.15070715	0.141598604
155029	F1571	N	F	24	LH	0.971698113	0.145092481	0.119068957
155029	F1571	N	F	24	RH	1.099952176	0.166169516	0.132108485
155029	F1571	N	F	26	LF	0.661824052	0.20828568	0.155643705
155029	F1571	N	F	26	RF	1.185983827	0.213869124	0.160575622
155029	F1571	N	F	26	LH	1.355820106	0.188064254	0.109372708

155029	F1571	N	F	26	RH	0.68414548	0.199403549	0.155267177
155029	F1571	N	F	28	LF	1.316689466	0.18216827	0.145734529
155029	F1571	N	F	28	RF	1.111111111	0.172753442	0.145325342
155029	F1571	N	F	28	LH	1.138638639	0.167023518	0.126494692
155029	F1571	N	F	28	RH	1.257071025	0.17439138	0.124448163
155029	F1571	N	F	96	LF	0.796296296	0.203376616	0.157155094
155029	F1571	N	F	96	RF	1.045751634	0.20695494	0.166015168
155029	F1571	N	F	96	LH	1.086956522	0.198029928	0.144808223
155029	F1571	N	F	96	RH	0.893141946	0.184891922	0.139177748
155362	F1158	N	M	0	LF	1.666666667	0.19144645	0.146987322
155362	F1158	N	M	0	RF	0.769230769	0.136099371	0.117952788
155362	F1158	N	M	0	LH	1.282051282	0.080260431	0.094136823
155362	F1158	N	M	0	RH	2.272727273	0.085596396	0.05262509
155362	F1158	N	M	1	LF	1.429738562	0.100605456	0.058664772
155362	F1158	N	M	1	RF	2.543290043	0.079425041	0.060120337
155362	F1158	N	M	1	LH	1.694444444	0.108945596	0.089090462
155362	F1158	N	M	1	RH	1.315789474	0.125847734	0.093178235
155362	F1158	N	M	2	LF	1.925925926	0.089184696	0.066605585
155362	F1158	N	M	2	RF	1.637214137	0.089711158	0.067887187
155362	F1158	N	M	2	LH	1.609756098	0.069593263	0.060026819
155362	F1158	N	M	2	RH	1.78125	0.090056475	0.071047805
155362	F1158	N	M	4	LF	2.041666667	0.206395261	0.1407364
155362	F1158	N	M	4	RF	1.93452381	0.193497427	0.140326556
155362	F1158	N	M	4	LH	2.116402116	0.172388333	0.109066254
155362	F1158	N	M	4	RH	2.579365079	0.150883704	0.098122677
155362	F1158	N	M	6	LF	4.435483871	0.077071352	0.065554048
155362	F1158	N	M	6	RF	1.547619048	0.097571041	0.093921774
155362	F1158	N	M	6	LH	1.742788462	0.082073943	0.079372956
155362	F1158	N	M	6	RH	1.527777778	0.099789248	0.073947535
155362	F1158	N	M	8	LF	1.424772036	0.186321979	0.157379723
155362	F1158	N	M	8	RF	1.433236575	0.172455988	0.122670919
155362	F1158	N	M	8	LH	2.175925926	0.116759663	0.077329409
155362	F1158	N	M	8	RH	2.223320158	0.105935902	0.060603722

155362	F1158	N	M	24	LF	1.26984127	0.16808573	0.14324617
155362	F1158	N	M	24	RF	1.172940535	0.173231804	0.124088492
155362	F1158	N	M	24	LH	1.330532213	0.181903	0.117004083
155362	F1158	N	M	24	RH	1.226359338	0.167815918	0.129180101
155362	F1158	N	M	26	LF	1.787994891	0.193633183	0.152531252
155362	F1158	N	M	26	RF	1.794871795	0.200121341	0.138251949
155362	F1158	N	M	26	LH	1.961538462	0.181903	0.141622501
155362	F1158	N	M	26	RH	1.893408135	0.185166275	0.115832715
155362	F1158	N	M	28	LF	1.619433198	0.186610071	0.143198707
155362	F1158	N	M	28	RF	1.587301587	0.198342892	0.161731136
155362	F1158	N	M	28	LH	1.515151515	0.204360709	0.1380667
155362	F1158	N	M	28	RH	1.492869875	0.173127096	0.110555478
160013	F1546	N	M	0	LF	0.892857143	0.027379034	0.019948204
160013	F1546	N	M	0	RF	1.162790698	-0.019712411	-0.027818624
160013	F1546	N	M	0	LH	1.19047619	0.0247251	0.007025838
160013	F1546	N	M	0	RH	0.819672131	0.050104304	0.036321965
160013	F1546	N	M	1	LF	1.97172619	0.106419271	0.084613759
160013	F1546	N	M	1	RF	1.844532279	0.124032599	0.088029265
160013	F1546	N	M	1	LH	2.386363636	0.089706501	0.055418207
160013	F1546	N	M	1	RH	1.920289855	0.107703222	0.064242751
160013	F1546	N	M	2	LF	1.503094607	0.13875633	0.103780321
160013	F1546	N	M	2	RF	1.482127289	0.120885812	0.084413008
160013	F1546	N	M	2	LH	1.537744641	0.100309484	0.081232018
160013	F1546	N	M	2	RH	1.823607427	0.108821476	0.079506176
160013	F1546	N	M	4	LF	1.253132832	0.124325546	0.08726237
160013	F1546	N	M	4	RF	1.414728682	0.137577757	0.1133613
160013	F1546	N	M	4	LH	1.253132832	0.162395453	0.097279296
160013	F1546	N	M	4	RH	1.335470085	0.141813744	0.098255094
160013	F1546	N	M	6	LF	1.136950904	0.131203846	0.096911456
160013	F1546	N	M	6	RF	1.302083333	0.131093949	0.104825135
160013	F1546	N	M	6	LH	1.257763975	0.124050671	0.099667025
160013	F1546	N	M	6	RH	1.231231231	0.134306447	0.09611099
160013	F1546	N	M	8	LF	1.707175926	0.138863143	0.098458142

160013	F1546	N	M	8	RF	1.668520578	0.148643379	0.111784318
160013	F1546	N	M	8	LH	1.583820663	0.15757028	0.113784088
160013	F1546	N	M	8	RH	1.602564103	0.151402538	0.10124466
160013	F1546	N	M	24	LF	1.527777778	0.126526214	0.095319577
160013	F1546	N	M	24	RF	1.533882784	0.138106236	0.092795047
160013	F1546	N	M	24	LH	1.55075188	0.136011063	0.094943033
160013	F1546	N	M	24	RH	1.619644723	0.125856342	0.081066476
160013	F1546	N	M	26	LF	1.56402737	0.133118612	0.091206946
160013	F1546	N	M	26	RF	1.56402737	0.143898122	0.110862157
160013	F1546	N	M	26	LH	1.495535714	0.155035111	0.099747726
160013	F1546	N	M	26	RH	1.541745731	0.137694806	0.087621677
160013	F1546	N	M	28	LF	1.40625	0.118385585	0.095793508
160013	F1546	N	M	28	RF	1.415470494	0.114333987	0.08821117
160013	F1546	N	M	28	LH	1.431451613	0.110580852	0.078158626
160013	F1546	N	M	28	RH	1.351351351	0.124857466	0.093134278
160013	F1546	N	M	96	LF	1.130589431	0.164643866	0.128315984
160013	F1546	N	M	96	RF	1.086956522	0.169988114	0.126084869
160013	F1546	N	M	96	LH	1.191151446	0.163692615	0.123347157
160013	F1546	N	M	96	RH	1.138716356	0.157602703	0.113920528
160021	F1768	N	M	0	LF	2.065826331	0.065721223	0.030531224
160021	F1768	N	M	0	RF	1.099033816	0.106407098	0.083302421
160021	F1768	N	M	0	LH	1.352813853	0.066197747	0.057471486
160021	F1768	N	M	0	RH	0.986460348	0.141357529	0.129306666
160021	F1768	N	M	1	LF	1.818783069	0.171367487	0.120838503
160021	F1768	N	M	1	RF	2.03125	0.139599294	0.108398982
160021	F1768	N	M	1	LH	1.844532279	0.132809682	0.087894621
160021	F1768	N	M	1	RH	1.776960784	0.173420969	0.097555018
160021	F1768	N	M	2	LF	1.393188854	0.105318461	0.08145256
160021	F1768	N	M	2	RF	0.867346939	0.127200419	0.098039212
160021	F1768	N	M	2	LH	0.836097291	0.140750333	0.102394314
160021	F1768	N	M	2	RH	1.302083333	0.099279911	0.079833479
160021	F1768	N	M	4	LF	1.726190476	0.166190574	0.107990118
160021	F1768	N	M	4	RF	1.56402737	0.154648044	0.12135241

160021	F1768	N	M	4	LH	1.515151515	0.142397553	0.119810637
160021	F1768	N	M	4	RH	1.742788462	0.163087309	0.117605831
160021	F1768	N	M	6	LF	1.695402299	0.163613032	0.116568788
160021	F1768	N	M	6	RF	1.643318966	0.133215782	0.101170905
160021	F1768	N	M	6	LH	1.527777778	0.1429785	0.102580094
160021	F1768	N	M	6	RH	1.590909091	0.144434009	0.095091958
160021	F1768	N	M	8	LF	1.52972028	0.160178024	0.12179789
160021	F1768	N	M	8	RF	1.442307692	0.151922216	0.109243345
160021	F1768	N	M	8	LH	1.31661442	0.153152162	0.118909366
160021	F1768	N	M	8	RH	1.494107744	0.150066596	0.111332271
160021	F1768	N	M	24	LF	1.38996139	0.208343535	0.144867806
160021	F1768	N	M	24	RF	1.520737327	0.186154927	0.139577648
160021	F1768	N	M	24	LH	1.597363083	0.175996594	0.113836668
160021	F1768	N	M	24	RH	1.56402737	0.190267879	0.129092678
160021	F1768	N	M	26	LF	1.076388889	0.161817219	0.105411991
160021	F1768	N	M	26	RF	1.089015152	0.146194963	0.121651408
160021	F1768	N	M	26	LH	1.006441224	0.147047505	0.114855249
160021	F1768	N	M	26	RH	1.068181818	0.156308037	0.107269366
160021	F1768	N	M	28	LF	1.695402299	0.167973137	0.124357492
160021	F1768	N	M	28	RF	1.482127289	0.149521648	0.120972428
160021	F1768	N	M	28	LH	1.471861472	0.129503392	0.109402924
160021	F1768	N	M	28	RH	1.587301587	0.173491129	0.119999098
160021	F1768	N	M	96	LF	1.541745731	0.207876546	0.144126855
160021	F1768	N	M	96	RF	1.55075188	0.195826696	0.15145291
160021	F1768	N	M	96	LH	1.387846962	0.199910621	0.132520843
160021	F1768	N	M	96	RH	1.482127289	0.214282846	0.131143932
160096	F1034	N	F	0	LF	1.234756098	0.098043247	0.086021679
160096	F1034	N	F	0	RF	1.111660079	0.067695445	0.055868181
160096	F1034	N	F	0	LH	3.125	0.048791628	0.038330115
160096	F1034	N	F	0	RH	1.37012012	0.059821834	0.05747893
160096	F1034	N	F	1	LF	1.944444444	0.075164055	0.044120905
160096	F1034	N	F	1	RF	2.083333333	0.097381401	0.078274041
160096	F1034	N	F	1	LH	1.64021164	0.105008535	0.084806019

160096	F1034	N	F	1	RH	1.527777778	0.123479421	0.07725662
160096	F1034	N	F	2	LF	2.056451613	0.150858219	0.098203959
160096	F1034	N	F	2	RF	1.84811828	0.166382167	0.105980237
160096	F1034	N	F	2	LH	2.083333333	0.144970482	0.105566698
160096	F1034	N	F	2	RH	2.326839827	0.134206562	0.074859692
160096	F1034	N	F	4	LF	2.029220779	0.152039089	0.11243059
160096	F1034	N	F	4	RF	1.822250639	0.147923299	0.097912741
160096	F1034	N	F	4	LH	1.583820663	0.168174432	0.105377305
160096	F1034	N	F	4	RH	1.822250639	0.161115746	0.102965099
160096	F1034	N	F	6	LF	1.78125	0.158883209	0.11363292
160096	F1034	N	F	6	RF	1.887464387	0.156351759	0.102974137
160096	F1034	N	F	6	LH	1.675675676	0.166113422	0.11413054
160096	F1034	N	F	6	RH	1.781400966	0.173095608	0.105700839
160096	F1034	N	F	8	LF	1.298920378	0.127201953	0.098091493
160096	F1034	N	F	8	RF	1.471861472	0.129943967	0.099272901
160096	F1034	N	F	8	LH	1.471861472	0.135684937	0.087828761
160096	F1034	N	F	8	RH	1.236263736	0.137052152	0.088695806
160096	F1034	N	F	24	LF	1.250781739	0.159568534	0.111771511
160096	F1034	N	F	24	RF	1.204994193	0.177214106	0.124930565
160096	F1034	N	F	24	LH	1.19047619	0.178275735	0.123000372
160096	F1034	N	F	24	RH	1.234756098	0.167110971	0.10437087
160096	F1034	N	F	26	LF	1.408730159	0.175978785	0.127748077
160096	F1034	N	F	26	RF	1.538825758	0.188275805	0.126314947
160096	F1034	N	F	26	LH	1.37012012	0.206424211	0.127686531
160096	F1034	N	F	26	RH	1.282051282	0.229414454	0.13848943
160096	F1034	N	F	28	LF	1.313164894	0.182836176	0.123637757
160096	F1034	N	F	28	RF	1.26984127	0.219432367	0.147653541
160096	F1034	N	F	28	LH	1.431451613	0.18929759	0.137625636
160096	F1034	N	F	28	RH	1.443089431	0.193506209	0.112307225
160096	F1034	N	F	96	LF	1.707175926	0.230708735	0.162101832
160096	F1034	N	F	96	RF	1.732377539	0.222720252	0.1492357
160096	F1034	N	F	96	LH	1.628151261	0.233602162	0.137206507
160096	F1034	N	F	96	RH	1.696832579	0.229934548	0.141492117

160121	F1546	N	F	0	LF	1.219512195	0.04222624	0.038554394
160121	F1546	N	F	0	RF	1.162790698	0.082311478	0.03425489
160121	F1546	N	F	0	LH	1.388888889	0.090105152	0.059144815
160121	F1546	N	F	0	RH	1.219512195	0.044980125	0.031255084
160121	F1546	N	F	1	LF	1.349431818	0.13928507	0.098626631
160121	F1546	N	F	1	RF	1.257071025	0.153694474	0.106938882
160121	F1546	N	F	1	LH	1.220238095	0.16849711	0.128696725
160121	F1546	N	F	1	RH	1.313131313	0.144197264	0.104348905
160121	F1546	N	F	2	LF	1.956815115	0.135713194	0.110817562
160121	F1546	N	F	2	RF	1.339285714	0.133579746	0.105839149
160121	F1546	N	F	2	LH	1.568181818	0.113587774	0.090281311
160121	F1546	N	F	2	RH	0.135713194	0.132814665	0.073881398
160121	F1546	N	F	4	LF	1.785714286	0.126166613	0.106815011
160121	F1546	N	F	4	RF	1.724137931	0.112423593	0.090754835
160121	F1546	N	F	4	LH	1.351351351	0.140302401	0.105973059
160121	F1546	N	F	4	RH	1.612903226	0.125358036	0.089385971
160121	F1546	N	F	6	LF	1.185879757	0.175040209	0.13717657
160121	F1546	N	F	6	RF	1.135204082	0.183736405	0.136464103
160121	F1546	N	F	6	LH	1.172940535	0.184578194	0.137131825
160121	F1546	N	F	6	RH	1.135204082	0.186286589	0.133867936
160121	F1546	N	F	8	LF	1.333767079	0.103270646	0.093031351
160121	F1546	N	F	8	RF	0.981902195	0.092643782	0.086247443
160121	F1546	N	F	8	LH	1.157407407	0.08493649	0.069578202
160121	F1546	N	F	8	RH	1.067730802	0.116278012	0.092076709
160121	F1546	N	F	24	LF	1.430250784	0.197655359	0.149870642
160121	F1546	N	F	24	RF	1.387846962	0.205196968	0.153604887
160121	F1546	N	F	24	LH	1.267209011	0.204441109	0.158003012
160121	F1546	N	F	24	RH	1.313164894	0.219486394	0.134233188
160121	F1546	N	F	26	LF	1.590909091	0.176954848	0.126668269
160121	F1546	N	F	26	RF	1.607142857	0.167108585	0.118476154
160121	F1546	N	F	26	LH	1.799242424	0.159497373	0.107523075
160121	F1546	N	F	26	RH	1.719114219	0.166863564	0.100889619
160121	F1546	N	F	28	LF	1.12487361	0.179524058	0.130939803

160121	F1546	N	F	28	RF	1.141670991	0.200781726	0.139150894
160121	F1546	N	F	28	LH	1.204994193	0.19589417	0.13805967
160121	F1546	N	F	28	RH	1.141670991	0.182406886	0.132119707
160121	F1546	N	F	96	LF	1.56402737	0.200140001	0.148637047
160121	F1546	N	F	96	RF	1.735294118	0.218449897	0.155663819
160121	F1546	N	F	96	LH	1.447477254	0.250810177	0.157559327
160121	F1546	N	F	96	RH	1.643318966	0.241463354	0.140476079
160153	F1768	N	M	0	LF	1.258894362	0.099691764	0.107938323
160153	F1768	N	M	0	RF	2.827380952	0.057031913	0.018412633
160153	F1768	N	M	0	LH	2.649456522	0.059800168	0.019789492
160153	F1768	N	M	0	RH	2.45215311	0.043128333	0.033486688
160153	F1768	N	M	1	LF	1.903735632	0.083709528	0.067230159
160153	F1768	N	M	1	RF	1.920289855	0.080751943	0.058083491
160153	F1768	N	M	1	LH	1.794871795	0.076933235	0.057816576
160153	F1768	N	M	1	RH	1.674107143	0.077757984	0.053153826
160153	F1768	N	M	2	LF	1.887464387	0.101103195	0.082443574
160153	F1768	N	M	2	RF	0.717871486	0.097498893	0.084247278
160153	F1768	N	M	2	LH	0.590277778	0.11003728	0.101095251
160153	F1768	N	M	2	RH	0.711040885	0.114158003	0.096572598
160153	F1768	N	M	4	LF	1.607142857	0.095619875	0.081279435
160153	F1768	N	M	4	RF	1.492869875	0.091131184	0.07262739
160153	F1768	N	M	4	LH	1.449579832	0.098532701	0.067420566
160153	F1768	N	M	4	RH	1.639784946	0.086365688	0.069712274
160153	F1768	N	M	6	LF	1.785714286	0.052736221	0.045334646
160153	F1768	N	M	6	RF	2.083333333	0.039783465	0.037449189
160153	F1768	N	M	6	LH	0.892857143	0.159133858	0.120275591
160153	F1768	N	M	6	RH	1.851851852	0.048110237	0.037007874
160153	F1768	N	M	8	LF	2.178030303	0.179390928	0.116155154
160153	F1768	N	M	8	RF	2.083333333	0.175759529	0.128869244
160153	F1768	N	M	8	LH	2.291666667	0.147999208	0.110702149
160153	F1768	N	M	8	RH	2.19047619	0.182822009	0.103740432
160153	F1768	N	M	24	LF	1.682692308	0.16644726	0.138247455
160153	F1768	N	M	24	RF	1.471825063	0.168504353	0.126619431

160153	F1768	N	M	24	LH	1.535682023	0.162735834	0.119317835
160153	F1768	N	M	24	RH	1.875	0.139752662	0.11101001
160153	F1768	N	M	26	LF	2.136363636	0.17681252	0.124067278
160153	F1768	N	M	26	RF	1.967592593	0.169452969	0.130699801
160153	F1768	N	M	26	LH	1.695402299	0.172736894	0.13718486
160153	F1768	N	M	26	RH	1.854395604	0.197335316	0.123502532
160153	F1768	N	M	28	LF	1.799242424	0.18853031	0.134015258
160153	F1768	N	M	28	RF	1.996927803	0.177876075	0.134634815
160153	F1768	N	M	28	LH	1.806451613	0.17218015	0.119231054
160153	F1768	N	M	28	RH	1.887464387	0.190238087	0.123789513
160153	F1768	N	M	96	LF	1.675824176	0.197975443	0.141701002
160153	F1768	N	M	96	RF	1.313164894	0.232485863	0.165039843
160153	F1768	N	M	96	LH	1.475694444	0.194793956	0.139049119
160153	F1768	N	M	96	RH	1.408730159	0.22861073	0.145783837
160777	F1745	N	M	1	LF	0.823211876	0.121598683	0.105850035
160777	F1745	N	M	1	RF	2.136363636	0.050441956	0.040890773
160777	F1745	N	M	1	LH	1.52972028	0.069281245	0.063431197
160777	F1745	N	M	1	RH	0.80624355	0.118556121	0.095232101
160777	F1745	N	M	2	LF	1.298920378	0.140339357	0.096920448
160777	F1745	N	M	2	RF	1.628151261	0.127348698	0.108607878
160777	F1745	N	M	2	LH	1.354166667	0.102635099	0.099704821
160777	F1745	N	M	2	RH	1.372180451	0.136022348	0.101138797
160777	F1745	N	M	4	LF	1.54293381	0.093836454	0.082230913
160777	F1745	N	M	4	RF	1.365248227	0.08835469	0.068925869
160777	F1745	N	M	4	LH	1.245498199	0.123982116	0.072315437
160777	F1745	N	M	4	RH	1.376811594	0.092646489	0.073129784
160777	F1745	N	M	6	LF	1.515151515	0.139429262	0.048492063
160777	F1745	N	M	6	RF	1.282051282	0.111295204	0.089846792
160777	F1745	N	M	6	LH	1.063829787	0.142500878	0.11936508
160777	F1745	N	M	6	RH	1.315789474	0.097916667	0.051289682
160777	F1745	N	M	8	LF	1.196509009	0.134258658	0.113088527
160777	F1745	N	M	8	RF	1.282894737	0.092446948	0.080355442
160777	F1745	N	M	8	LH	1.015176374	0.098456826	0.084078179

160777	F1745	N	M	8	RH	1.116071429	0.134133886	0.116346803
160777	F1745	N	M	24	LF	1.296647691	0.134258658	0.102644663
160777	F1745	N	M	24	RF	1.298920378	0.157278088	0.108181024
160777	F1745	N	M	24	LH	1.447477254	0.150344188	0.108655964
160777	F1745	N	M	24	RH	1.388888889	0.131943994	0.087501886
160777	F1745	N	M	26	LF	1.136950904	0.168721246	0.127988284
160777	F1745	N	M	26	RF	1.168478261	0.160795477	0.13103789
160777	F1745	N	M	26	LH	1.081454211	0.156117084	0.123499993
160777	F1745	N	M	26	RH	1.162790698	0.168828569	0.116473604
160777	F1745	N	M	28	LF	1.696712619	0.143324574	0.111690313
160777	F1745	N	M	28	RF	1.430250784	0.155900121	0.112363198
160777	F1745	N	M	28	LH	1.387846962	0.151433207	0.117272659
160777	F1745	N	M	28	RH	1.469404187	0.159232754	0.112440582
160777	F1745	N	M	96	LF	1.345050215	0.189129847	0.146212109
160777	F1745	N	M	96	RF	1.428571429	0.173479219	0.134110165
160777	F1745	N	M	96	LH	1.495535714	0.164740977	0.125810769
160777	F1745	N	M	96	RH	1.316689466	0.192879523	0.139336436
