# Supplementary material for: Does intrauterine crowding affect locomotor development? A comparative study of motor performance, neuromotor maturation and gait variability among piglets that differ in birth weight and vitality
Source: PLoS One. 2018 Apr 24;13(4):e0195961. doi: 10.1371/journal.pone.0195961 (PMC5915318; doi:10.1371/journal.pone.0195961)
Supplement: S4 Table — (PDF) [file pone.0195961.s004.pdf]

S4. NORMALIZED SELF-SELECTED SPEED

PIGLET	SOW	CATEGORY	GENDER	AGE (h)	SELF-SELECTED SPEED
151301	F1816	L	F	1	0.134482969
151301	F1816	L	F	2	0.117969541
151301	F1816	L	F	4	0.204767129
151301	F1816	L	F	6	0.153537825
151301	F1816	L	F	8	0.090678127
151301	F1816	L	F	24	0.097736225
151301	F1816	L	F	26	0.240648146
151301	F1816	L	F	28	0.276925437
151301	F1816	L	F	96	0.072191438
151302	F1816	L	F	0	0.111467506
151302	F1816	L	F	1	0.058897654
151302	F1816	L	F	2	0.132131306
151302	F1816	L	F	4	0.135915735
151302	F1816	L	F	6	0.117554109
151302	F1816	L	F	8	0.100256618
151302	F1816	L	F	24	0.120152714
151302	F1816	L	F	26	0.088855805
151302	F1816	L	F	28	0.055027906
151302	F1816	L	F	96	0.048727857
151306	F1349	L	F	4	0.116771044
151306	F1349	L	F	6	0.091385312
151309	F943	L	F	1	0.050053235
151309	F943	L	F	2	0.086998914
151309	F943	L	F	4	0.1040703
151309	F943	L	F	6	0.096637192
151309	F943	L	F	8	0.124946882
151309	F943	L	F	24	0.089968051
151309	F943	L	F	26	0.151222844
151309	F943	L	F	28	0.087645802

151310	F943	L	F	0	0.049487742
151310	F943	L	F	1	0.11328648
151310	F943	L	F	2	0.107148909
151310	F943	L	F	4	0.093348106
151310	F943	L	F	6	0.090911215
151310	F943	L	F	8	0.068308796
151310	F943	L	F	24	0.100641189
151310	F943	L	F	26	0.111842354
151310	F943	L	F	28	0.137882707
151310	F943	L	F	96	0.125722376
152686	F1158	L	F	1	0.029223536
152686	F1158	L	F	2	0.087022161
152686	F1158	L	F	4	0.129047769
152686	F1158	L	F	6	0.16122678
152686	F1158	L	F	8	0.176351097
152686	F1158	L	F	24	0.047762411
152686	F1158	L	F	26	0.137899336
152686	F1158	L	F	28	0.108304587
154983	F1571	L	F	4	0.147454468
154983	F1571	L	F	6	0.10966157
154983	F1571	L	F	8	0.11951019
159811	F1541	L	M	1	0.075905216
159811	F1541	L	M	2	0.056910457
159811	F1541	L	M	4	0.129499645
159811	F1541	L	M	6	0.130453389
159811	F1541	L	M	8	0.093870002
159811	F1541	L	M	24	0.1246021
159811	F1541	L	M	26	0.108989215
159811	F1541	L	M	28	0.204339403
159811	F1541	L	M	96	0.170867013
159828	F1546	L	F	0	0.104040421
159828	F1546	L	F	1	0.161494395
159828	F1546	L	F	2	0.078402531

159828	F1546	L	F	4	0.142411536
159828	F1546	L	F	6	0.095462048
159828	F1546	L	F	8	0.191570098
159828	F1546	L	F	24	0.151455946
159828	F1546	L	F	26	0.163743604
159828	F1546	L	F	28	0.228552812
159828	F1546	L	F	96	0.356146512
160446	F1546	L	M	0	0.061373176
160446	F1546	L	M	1	0.084347679
160446	F1546	L	M	2	0.147457058
160446	F1546	L	M	4	0.104251008
160446	F1546	L	M	6	0.110675528
160446	F1546	L	M	8	0.142123638
160446	F1546	L	M	24	0.103233713
160446	F1546	L	M	26	0.121854358
160446	F1546	L	M	28	0.0936044
160446	F1546	L	M	96	0.087077755
160639	F1546	L	F	0	0.063273226
160639	F1546	L	F	1	0.059919207
160639	F1546	L	F	2	0.109229805
160639	F1546	L	F	4	0.117897725
160639	F1546	L	F	6	0.219890706
160639	F1546	L	F	8	0.24141855
160639	F1546	L	F	24	0.12864369
160639	F1546	L	F	26	0.158199757
160639	F1546	L	F	28	0.124145942
160639	F1546	L	F	96	0.147882801
151303	F943	N	F	0	0.059518829
151303	F943	N	F	1	0.084929549
151303	F943	N	F	2	0.098785981
151303	F943	N	F	4	0.091612516
151303	F943	N	F	6	0.099773915
151303	F943	N	F	8	0.104853227

151303	F943	N	F	24	0.068877297
151303	F943	N	F	26	0.091815625
151303	F943	N	F	28	0.122598637
151303	F943	N	F	96	0.041277275
151307	F943	N	F	0	0.046794856
151307	F943	N	F	1	0.150857829
151307	F943	N	F	2	0.115554699
151307	F943	N	F	4	0.175361929
151307	F943	N	F	6	0.13435172
151307	F943	N	F	8	0.15528399
151307	F943	N	F	24	0.179574681
151307	F943	N	F	26	0.173846833
151307	F943	N	F	28	0.148738017
151307	F943	N	F	96	0.077949707
152750	F1571	N	F	0	0.066819409
152750	F1571	N	F	1	0.11699505
152750	F1571	N	F	2	0.169587552
152750	F1571	N	F	4	0.110154771
152750	F1571	N	F	6	0.154065573
152750	F1571	N	F	8	0.097010416
152750	F1571	N	F	24	0.109015625
152750	F1571	N	F	26	0.201839025
152750	F1571	N	F	28	0.261333488
152750	F1571	N	F	96	0.205299548
152776	F1571	N	M	0	0.144779551
152776	F1571	N	M	1	0.111343934
152776	F1571	N	M	2	0.153829873
152776	F1571	N	M	4	0.080601451
152776	F1571	N	M	6	0.159121926
152776	F1571	N	M	8	0.109637378
152776	F1571	N	M	24	0.148626533
152776	F1571	N	M	26	0.200560919
152776	F1571	N	M	28	0.239347096

152776	F1571	N	M	96	0.112486289
154850	F998	N	F	0	0.059715505
154850	F998	N	F	1	0.065780859
154850	F998	N	F	2	0.039436453
154850	F998	N	F	4	0.141466424
154850	F998	N	F	6	0.171620619
154850	F998	N	F	8	0.111816033
154850	F998	N	F	24	0.142321284
154850	F998	N	F	26	0.196516656
154850	F998	N	F	28	0.236346281
154850	F998	N	F	96	0.235446224
155005	F1158	N	M	0	0.044418433
155005	F1158	N	M	1	0.052313432
155005	F1158	N	M	2	0.091501129
155005	F1158	N	M	4	0.091952408
155005	F1158	N	M	6	0.116951288
155005	F1158	N	M	8	0.13220441
155005	F1158	N	M	24	0.188786663
155005	F1158	N	M	26	0.262802326
155005	F1158	N	M	28	0.329618307
155005	F1158	N	M	96	0.225068432
155029	F1571	N	F	0	0.062186852
155029	F1571	N	F	1	0.075992108
155029	F1571	N	F	2	0.093259153
155029	F1571	N	F	4	0.09056824
155029	F1571	N	F	8	0.050997199
155029	F1571	N	F	24	0.14961847
155029	F1571	N	F	26	0.076072809
155029	F1571	N	F	28	0.160610595
155029	F1571	N	F	96	0.080849154
155362	F1158	N	M	0	0.207379003
155362	F1158	N	M	1	0.118384707
155362	F1158	N	M	2	0.154386709

155362	F1158	N	M	4	0.157138355
155362	F1158	N	M	6	0.09570037
155362	F1158	N	M	8	0.09136587
155362	F1158	N	M	24	0.122528673
155362	F1158	N	M	26	0.238071501
155362	F1158	N	M	28	0.218109899
160013	F1546	N	M	0	0.037238653
160013	F1546	N	M	1	0.12849856
160013	F1546	N	M	2	0.141920683
160013	F1546	N	M	4	0.104937003
160013	F1546	N	M	6	0.091672379
160013	F1546	N	M	8	0.172149372
160013	F1546	N	M	24	0.145363079
160013	F1546	N	M	26	0.111841304
160013	F1546	N	M	28	0.121750166
160013	F1546	N	M	96	0.118347325
160021	F1768	N	M	0	0.087901206
160021	F1768	N	M	1	0.108130414
160021	F1768	N	M	2	0.124168268
160021	F1768	N	M	4	0.107282724
160021	F1768	N	M	6	0.109329329
160021	F1768	N	M	8	0.180729747
160021	F1768	N	M	24	0.211873017
160021	F1768	N	M	26	0.112203447
160021	F1768	N	M	28	0.223958082
160021	F1768	N	M	96	0.21035295
160096	F1034	N	F	0	0.078911053
160096	F1034	N	F	1	0.092842622
160096	F1034	N	F	2	0.179448315
160096	F1034	N	F	4	0.154052609
160096	F1034	N	F	6	0.161921444
160096	F1034	N	F	8	0.087196688
160096	F1034	N	F	24	0.123988562

160096	F1034	N	F	26	0.174530864
160096	F1034	N	F	28	0.150641454
160096	F1034	N	F	96	0.274615842
160121	F1546	N	F	0	0.077861228
160121	F1546	N	F	1	0.145864953
160121	F1546	N	F	2	0.188225885
160121	F1546	N	F	4	0.149823701
160121	F1546	N	F	6	0.12405291
160121	F1546	N	F	8	0.110495126
160121	F1546	N	F	24	0.21557799
160121	F1546	N	F	26	0.199888999
160121	F1546	N	F	28	0.154403732
160121	F1546	N	F	96	0.238268493
160153	F1768	N	M	0	0.098223123
160153	F1768	N	M	1	0.097706595
160153	F1768	N	M	2	0.091687635
160153	F1768	N	M	4	0.106186726
160153	F1768	N	M	6	0.078781835
160153	F1768	N	M	8	0.230592102
160153	F1768	N	M	24	0.261518279
160153	F1768	N	M	26	0.181762556
160153	F1768	N	M	28	0.190843685
160153	F1768	N	M	96	0.122260546
160777	F1745	N	M	1	0.077015045
160777	F1745	N	M	2	0.095785313
160777	F1745	N	M	4	0.075464121
160777	F1745	N	M	6	0.111327808
160777	F1745	N	M	8	0.097867383
160777	F1745	N	M	24	0.115876443
160777	F1745	N	M	26	0.142468681
160777	F1745	N	M	28	0.174766044
160777	F1745	N	M	96	0.145428038
