# Supplementary material for: Does intrauterine crowding affect locomotor development? A comparative study of motor performance, neuromotor maturation and gait variability among piglets that differ in birth weight and vitality
Source: PLoS One. 2018 Apr 24;13(4):e0195961. doi: 10.1371/journal.pone.0195961 (PMC5915318; doi:10.1371/journal.pone.0195961)
Supplement: S5 Table — (PDF) [file pone.0195961.s005.pdf]

S5. NORMALIZED SPATIO-TEMPORAL GAIT VARIABLES

PIGLET	SOW	CATEGORY	GENDER	AGE (h)	LEG	DUTY FACTOR	STRIDE FREQ	STRIDE LENGTH	STEP LENGTH	STANCE DURATION	SWING DURATION	MAX HEIGHT
151301	F1816	L	F	1	LF	0.777670528	0.131197151	0.990970974	0.78980467	5.918121322	1.774534856	0.125278979
151301	F1816	L	F	1	RF	0.814336918	0.141154985	0.671127714	0.690049768	6.062909536	1.366791397	0.115961292
151301	F1816	L	F	1	LH	0.694388271	0.11600936	0.667727143	0.446093832	6.050212544	2.607840068	0.165552064
151301	F1816	L	F	1	RH	0.699134199	0.139909903	0.849684715	0.564197421	5.185164852	2.131490347	0.116932887
151301	F1816	L	F	2	LF	0.649054505	0.180666682	0.84651168	0.617769032	3.59278123	1.942284692	0.208626515
151301	F1816	L	F	2	RF	0.742263484	0.162162939	0.572323195	0.553623038	4.677389527	1.572887229	0.035404019
151301	F1816	L	F	2	LH	0.872196478	0.074628035	1.013424959	0.775220815	11.95728835	1.763481983	0.105729234
151301	F1816	L	F	2	RH	0.677922078	0.159392492	0.810174511	0.636018861	4.253966847	2.019894001	0.103797936
151301	F1816	L	F	4	LF	0.554487179	0.221748069	0.877521295	0.389945617	2.527861216	1.98830998	0.088814308
151301	F1816	L	F	4	RF	0.715	0.20952623	1.180054549	0.850484788	3.431859478	1.355129185	0.126865722
151301	F1816	L	F	4	LH	0.764092664	0.153884098	1.134219	0.846381106	4.968343968	1.535789924	0.205829004
151301	F1816	L	F	4	RH	0.668981481	0.217797999	0.921981353	0.668673133	3.071232566	1.535095358	0.130678433
151301	F1816	L	F	6	LF	0.722527473	0.185519236	0.86772609	0.608716165	4.028515853	1.55904906	0.1915168
151301	F1816	L	F	6	RF	0.66802168	0.170414296	0.807437537	0.622634552	4.343217186	1.900538328	0.143265521
151301	F1816	L	F	6	LH	0.660714286	0.168232348	0.70032366	0.51764421	4.2408365	1.995359739	0.122026917
151301	F1816	L	F	6	RH	0.622895623	0.186003566	0.855131829	0.518729369	3.497383763	1.987800464	0.126872147
151301	F1816	L	F	8	LF	0.809782609	0.15366534	0.817145367	0.599012543	7.171798054	1.37632637	0.16424115
151301	F1816	L	F	8	RF	0.807761194	0.150263457	0.761148459	0.623909256	7.171798054	1.282990877	0.137198155
151301	F1816	L	F	8	LH	0.81031746	0.140396092	0.782685606	0.663730382	7.352824748	1.560739639	0.090689315
151301	F1816	L	F	8	RH	0.728571429	0.169442724	0.541506184	0.31667936	6.160911647	2.206186082	0.084344346
151301	F1816	L	F	24	LF	0.732352941	0.187929402	0.77413195	0.546752997	3.924972436	1.41899912	0.174576624
151301	F1816	L	F	24	RF	0.709663866	0.173643021	0.790841618	0.606169578	4.091463273	1.669213751	0.165763996
151301	F1816	L	F	24	LH	0.699919872	0.170413341	0.733727832	0.556895603	4.176143819	1.752937545	0.134237412
151301	F1816	L	F	24	RH	0.640990991	0.180808554	0.800800217	0.498128381	3.591034011	2.004108927	0.119654551
151301	F1816	L	F	26	LF	0.690909091	0.236459061	0.874104966	0.658732609	2.997463601	1.332282967	0.105697116
151301	F1816	L	F	26	RF	0.714864865	0.18116658	0.855679301	0.661128815	3.996848902	1.582244676	0.123401172
151301	F1816	L	F	26	LH	0.745082612	0.170017059	0.834042982	0.64639356	4.496080018	1.499077951	0.1662747
151301	F1816	L	F	26	RH	0.577764977	0.204028493	0.878603618	0.572722989	2.831591685	2.081706559	0.088947775
151301	F1816	L	F	28	LF	0.604347826	0.244375614	1.182271118	0.755034189	2.477004232	1.622928251	0.157408973
151301	F1816	L	F	28	RF	0.7	0.234172289	1.156430817	0.831423112	2.98944982	1.280930084	0.10999143

151301	F1816	L	F	28	LH	0.611263736	0.217141345	1.192905232	0.779218673	2.818634624	1.793743447	0.125147774
151301	F1816	L	F	28	RH	0.66	0.234172289	1.214951047	0.794559809	2.81826685	1.452113055	0.148988586
151301	F1816	L	F	96	LF	0.6875	0.201174726	1.063979639	0.624443001	5.371544741	1.466937046	0.149827318
151301	F1816	L	F	96	RF	0.570048309	0.236982405	1.219234616	0.547181685	3.256007074	1.955013008	0.154905806
151301	F1816	L	F	96	LH	0.565789474	0.272319279	1.213988287	0.55242801	2.605239125	1.791779189	0.121903804
151301	F1816	L	F	96	RH	0.625	0.208034753	1.203506089	0.536598781	4.23270083	1.955013008	0.056903973
151302	F1816	L	F	0	LF	0.754545455	0.121382503	0.919900634	0.807962377	6.691634367	1.902816882	0.220062303
151302	F1816	L	F	0	RF	0.836174636	0.106049159	0.916188804	0.71177715	8.377339707	1.572308879	0.172260434
151302	F1816	L	F	0	LH	0.66426799	0.118658312	0.699175244	0.414470102	6.811932112	2.515425988	0.101787079
151302	F1816	L	F	0	RH	0.512987013	0.157041282	0.804959994	0.583852754	4.046229254	3.501389805	0.229500861
151302	F1816	L	F	1	LF	0.821890547	0.103425614	0.599645475	0.464953231	8.46801616	1.614901155	0.097738584
151302	F1816	L	F	1	RF	0.886303327	0.07945552	0.490918102	0.437202116	11.15134379	1.435078441	0.077433104
151302	F1816	L	F	1	LH	0.727921196	0.067501698	0.484522827	0.39419394	10.96451453	5.099840574	0.083733157
151302	F1816	L	F	1	RH	0.66591794	0.096655907	0.545835366	0.343968708	7.118761926	3.316794275	0.111424622
151302	F1816	L	F	2	LF	0.74	0.227012922	0.718445708	0.585476283	3.262478222	1.144069752	0.170613601
151302	F1816	L	F	2	RF	0.669642857	0.189787287	0.596447803	0.433682503	3.525238379	1.756088305	0.122686322
151302	F1816	L	F	2	LH	0.667410714	0.189787287	0.44363484	0.382868936	3.521972937	1.759353747	0.174519205
151302	F1816	L	F	2	RH	0.587857143	0.215076819	0.605855107	0.37985933	2.730427022	1.945411993	0.201958047
151302	F1816	L	F	4	LF	0.751851852	0.206352034	0.65792614	0.481591193	3.674723793	1.192365493	0.13833262
151302	F1816	L	F	4	RF	0.751891892	0.196910772	0.735702437	0.5413437	4.02274688	1.28104835	0.117527992
151302	F1816	L	F	4	LH	0.703801945	0.17652323	0.699955258	0.574754086	4.104721395	1.708809839	0.142042872
151302	F1816	L	F	4	RH	0.706233422	0.213905103	0.650342799	0.490092259	3.333409048	1.363022866	0.095187185
151302	F1816	L	F	6	LF	0.709291188	0.175521141	0.70309428	0.496445366	4.063535931	1.675745776	0.137058158
151302	F1816	L	F	6	RF	0.79765131	0.172790414	0.634650555	0.506156055	4.759725213	1.23121351	0.111931916
151302	F1816	L	F	6	LH	0.695	0.182737848	0.582623094	0.442534842	4.043717059	1.67970955	0.164225335
151302	F1816	L	F	6	RH	0.671875	0.17662213	0.664987262	0.411726266	3.796023817	1.868621024	0.176939749
151302	F1816	L	F	8	LF	0.719703575	0.174678517	0.643910648	0.502305286	4.146706922	1.609095757	0.115448684
151302	F1816	L	F	8	RF	0.760188088	0.190953604	0.537927381	0.404573777	3.982261237	1.267539049	0.107979424
151302	F1816	L	F	8	LH	0.692226891	0.191863892	0.549979587	0.322684481	3.638171461	1.609095757	0.125311202
151302	F1816	L	F	8	RH	0.661458333	0.214667209	0.506664391	0.39279935	3.129636001	1.606562689	0.093735713
151302	F1816	L	F	24	LF	0.779279279	0.178898597	0.801383573	0.602078657	4.432129273	1.191900422	0.133590274
151302	F1816	L	F	24	RF	0.79029304	0.181557526	0.787047382	0.639287577	4.441096573	1.173965821	0.123885314
151302	F1816	L	F	24	LH	0.651351351	0.178898597	0.765263435	0.490152776	3.685763738	1.938265957	0.179378003

151302	F1816	L	F	24	RH	0.643951613	0.169602887	0.817477532	0.494057475	3.854113662	2.102132231	0.181111829
151302	F1816	L	F	26	LF	0.791666667	0.146489892	0.62698066	0.494070942	5.463663626	1.451554741	0.125178924
151302	F1816	L	F	26	RF	0.784804754	0.171605834	0.46411479	0.384663502	4.609762872	1.280851131	0.107178342
151302	F1816	L	F	26	LH	0.688501742	0.155155807	0.157124136	0.35625929	4.43829385	2.049591434	0.116125397
151302	F1816	L	F	26	RH	0.639653816	0.165978425	0.558130182	0.372213689	3.92733114	2.219912337	0.193524581
151302	F1816	L	F	28	LF	0.82037037	0.185090802	0.639122115	0.48282054	4.701835595	0.921433309	0.097856154
151302	F1816	L	F	28	RF	0.79503367	0.17834363	0.726167048	0.588449727	4.870953574	1.089156795	0.086374033
151302	F1816	L	F	28	LH	0.672619048	0.177596858	0.785046871	0.509868029	4.030244402	1.844958354	0.14402432
151302	F1816	L	F	28	RH	0.607692308	0.175906659	0.714379651	0.436916679	3.524284959	2.26531294	0.192933961
151302	F1816	L	F	96	LF	0.634615385	0.120230067	0.840131801	0.56145573	5.279939553	3.037533496	0.061538964
151302	F1816	L	F	96	RF	0.817948718	0.112240506	0.79780719	0.680298454	7.360851011	1.598485251	0.047695481
151302	F1816	L	F	96	LH	0.774813896	0.110502912	0.863499014	0.628864188	7.036833014	2.077825067	0.06125427
151302	F1816	L	F	96	RH	0.596666667	0.114652841	0.850891028	0.561218174	5.28096835	3.518930905	0.091615171
151306	F1349	L	F	4	LF	0.656498674	0.18569059	0.736862135	0.52294484	3.536874535	1.868633679	0.129909565
151306	F1349	L	F	4	RF	0.715725806	0.161593963	0.782042742	0.539142008	4.424342996	1.764537475	0.116361217
151306	F1349	L	F	4	LH	0.80726601	0.178653321	0.731864534	0.586681319	4.519339684	1.081361629	0.080179162
151306	F1349	L	F	4	RH	0.74066092	0.193658854	0.622935403	0.481892064	3.830964114	1.372851347	0.088129457
151306	F1349	L	F	6	LF	0.847	0.131133703	0.929772677	0.764189433	14.32485296	1.306507901	0.115081197
151306	F1349	L	F	6	RF	0.801333333	0.11863488	1.134516578	0.942561437	14.21758152	1.691583262	0.107948888
151306	F1349	L	F	6	LH	0.625232256	0.137299371	1.012032895	0.380864993	8.898020121	5.782567942	0.051179164
151306	F1349	L	F	6	RH	0.43697479	0.143791592	0.940503105	0.490537647	10.21003706	8.147120072	0.123466507
151309	F943	L	F	1	LF	0.833333333	0.064970637	0.75364197	0.360905197	12.82630702	2.565261403	0.299786567
151309	F943	L	F	1	RF	0.838235294	0.068792439	0.922178234	0.595296511	12.18499167	2.35148962	0.280445498
151309	F943	L	F	1	LH	0.645833333	0.097455955	0.436719099	0.502867798	6.626925292	3.634120321	0.232092828
151309	F943	L	F	1	RH	0.75	0.053157794	0.701304072	0.45954071	14.10893772	4.702979239	0.058023204
151309	F943	L	F	2	LF	0.762337662	0.064144921	1.539982221	1.040137677	11.91155664	3.72352919	0.160721145
151309	F943	L	F	2	RF	0.64576347	0.070412068	1.61908161	0.904362586	9.241979055	5.000815984	0.231783887
151309	F943	L	F	2	LH	0.755	0.060753639	1.877818658	1.256009422	12.44253612	4.041849968	0.212845258
151309	F943	L	F	2	RH	0.787095919	0.077787584	1.203433426	0.856636827	10.4096001	2.866221566	0.037620033
151309	F943	L	F	4	LF	0.8125	0.15831226	1.031634782	0.854473233	5.190611627	1.161377902	0.1448813
151309	F943	L	F	4	RF	0.642642643	0.123840114	1.084084475	0.73494653	5.17580156	2.903444754	0.155824096
151309	F943	L	F	4	LH	0.647286822	0.136996179	1.053019484	0.609555597	4.900267152	2.716770751	0.134774964
151309	F943	L	F	4	RH	0.539881345	0.130738067	1.163295288	0.704339387	4.168493129	3.602614245	0.145090414

151309	F943	L	F	6	LF	0.758653846	0.16323275	1.10088165	0.821371226	4.955565841	1.457807277	0.18071608
151309	F943	L	F	6	RF	0.566081871	0.124855899	1.178161553	0.781423222	4.663961824	3.401904986	0.153311267
151309	F943	L	F	6	LH	0.646212121	0.144198234	1.035477821	0.611745833	4.664281031	2.526560927	0.149257464
151309	F943	L	F	6	RH	0.627826087	0.158795181	1.084636794	0.703713743	4.469488212	2.429430523	0.165256721
151309	F943	L	F	8	LF	0.716463415	0.144132782	1.037325907	0.789338265	5.117034506	2.090242946	0.15379993
151309	F943	L	F	8	RF	0.747222222	0.109991715	1.299935069	0.96235857	7.190401764	2.29578663	0.185556845
151309	F943	L	F	8	LH	0.746302907	0.109854239	1.260812747	0.860031221	6.985912811	2.397503742	0.176382499
151309	F943	L	F	8	RH	0.714198286	0.118798425	1.202090278	0.915585522	6.055692581	2.399613203	0.058926653
151309	F943	L	F	24	LF	0.799003322	0.114591818	0.682597814	0.526514546	6.967306903	1.844611206	0.149755798
151309	F943	L	F	24	RF	0.757133152	0.09731105	0.891620916	0.68180915	7.970764536	2.484915251	0.234648564
151309	F943	L	F	24	LH	0.855902778	0.154879612	0.578720554	0.522552573	5.543272483	0.968497346	0.066809943
151309	F943	L	F	24	RH	0.745299647	0.091597133	0.897135563	0.550120077	8.445442675	2.934071969	0.206251027
151309	F943	L	F	26	LF	0.738636364	0.138062596	1.21309532	0.8777579	5.608941707	1.960521385	0.139836561
151309	F943	L	F	26	RF	0.694827586	0.160500713	1.10435147	0.822285933	4.483675748	1.960521385	0.176797015
151309	F943	L	F	26	LH	0.693223443	0.165571706	1.095939508	0.680414984	4.397148621	1.860953193	0.206464483
151309	F943	L	F	26	RH	0.682170543	0.144422738	1.21049827	0.790735338	4.855866387	2.237490853	0.146045686
151309	F943	L	F	28	LF	0.812750455	0.098645174	0.953530225	0.781500859	8.399167964	1.952440102	0.203648172
151309	F943	L	F	28	RF	0.784700722	0.082420716	1.041736475	0.8166418	9.66514397	2.547502311	0.164256282
151309	F943	L	F	28	LH	0.810026738	0.08404039	0.964245967	0.736945325	9.766441501	2.249264375	0.215199965
151309	F943	L	F	28	RH	0.742248062	0.098070318	0.971550074	0.709473696	8.095275374	2.544674984	0.16425628
151310	F943	L	F	0	LF	0.875	0.073646393	0.86408837	0.601104953	11.88109787	1.697299695	0.413259653
151310	F943	L	F	0	RF	0.621621622	0.127388355	0.601419965	0.347828813	4.879736624	2.970274467	0.450828716
151310	F943	L	F	0	LH	0.782608696	0.204929093	0.187845295	0.129165275	3.818924314	1.06081231	0.009392264
151310	F943	L	F	0	RH	0.652777778	0.06546346	1.220994441	0.594052843	9.971635709	5.304061548	0.112707179
151310	F943	L	F	1	LF	0.690322581	0.127847395	0.893940279	0.707949712	5.727955492	2.343860149	0.181300281
151310	F943	L	F	1	RF	0.691071429	0.104206422	0.820730083	0.553968385	7.202208639	3.19718988	0.150670144
151310	F943	L	F	1	LH	0.619607843	0.209004587	0.514887732	0.339131859	3.523928858	1.903369041	0.101602211
151310	F943	L	F	1	RH	0.616666667	0.32082741	0.29452071	0.205214649	2.802199624	1.118290587	0.077934835
151310	F943	L	F	2	LF	0.909392933	0.087984175	0.739163256	0.613856469	10.97309523	1.016115838	0.174581247
151310	F943	L	F	2	RF	0.803659081	0.074524259	0.842179703	0.573997429	11.25991419	2.486720124	0.168967734
151310	F943	L	F	2	LH	0.816020025	0.080190141	0.847587873	0.563682335	10.7818826	2.402646426	0.189182941
151310	F943	L	F	2	RH	0.737934905	0.137876061	0.554879591	0.341609507	8.007865607	2.103816939	0.132161495
151310	F943	L	F	4	LF	0.820512821	0.182329174	1.12729177	0.853773725	4.550841654	0.987010348	0.141445576

151310	F943	L	F	4	RF	0.722105263	0.23393658	0.851487129	0.662179861	3.142542037	1.184412418	0.110118442
151310	F943	L	F	4	LH	0.664351852	0.199177485	0.925738956	0.674889347	3.345241132	1.680566105	0.177519022
151310	F943	L	F	4	RH	0.644252874	0.171973434	1.176245523	0.697679633	3.750639323	2.075370244	0.150938201
151310	F943	L	F	6	LF	0.723484848	0.139664748	0.91029198	0.679649619	5.383530063	2.009478107	0.107849035
151310	F943	L	F	6	RF	0.821819425	0.143924717	0.904019491	0.713937077	5.754940755	1.276785631	0.153652574
151310	F943	L	F	6	LH	0.696022727	0.147455792	0.881946911	0.583827224	4.83798992	2.100160299	0.149008681
151310	F943	L	F	6	RH	0.628342246	0.142467592	0.923706337	0.538097988	4.560155395	2.562253184	0.070353983
151310	F943	L	F	8	LF	0.849906191	0.110082573	0.707010629	0.534543478	8.047295833	1.429675163	0.125226612
151310	F943	L	F	8	RF	0.867822967	0.13671116	0.255945302	0.196228457	6.388779758	0.995259664	0.102284448
151310	F943	L	F	8	LH	0.671417854	0.155077721	0.310124798	0.168623913	4.516092233	2.161706469	0.126861139
151310	F943	L	F	8	RH	0.699148379	0.103296947	0.805912872	0.496638381	7.390112022	2.797398126	0.139415149
151310	F943	L	F	24	LF	0.845321637	0.137008077	0.891399098	0.757531204	6.180707585	1.142499602	0.077038747
151310	F943	L	F	24	RF	0.830907055	0.135024841	0.865969894	0.686771151	6.30466658	1.233299291	0.060473531
151310	F943	L	F	24	LH	0.781578947	0.131509972	0.937034702	0.709947309	6.127804247	1.68256069	0.128741565
151310	F943	L	F	24	RH	0.64787234	0.130638383	0.8523447	0.524764941	4.952145338	2.724786516	0.119283546
151310	F943	L	F	26	LF	0.780222694	0.134557648	0.903217861	0.709484324	5.824812841	1.628399984	0.126847796
151310	F943	L	F	26	RF	0.805555556	0.123437352	0.885739832	0.699999963	6.595676357	1.626430041	0.105352371
151310	F943	L	F	26	LH	0.769556025	0.120841913	0.900759886	0.658408915	6.507690108	1.885792254	0.120742258
151310	F943	L	F	26	RH	0.760416667	0.127071462	0.900847818	0.69053653	5.996845453	1.885135607	0.102893811
151310	F943	L	F	28	LF	0.755952381	0.134699724	1.132342551	0.846643485	5.61552877	1.814575016	0.140524192
151310	F943	L	F	28	RF	0.770348837	0.157615858	1.096643399	0.815843544	5.005513616	1.468066863	0.119473805
151310	F943	L	F	28	LH	0.794208494	0.160932567	1.124142507	0.866336307	4.921545561	1.299163849	0.156408178
151310	F943	L	F	28	RH	0.746794872	0.139935553	1.142776234	0.766600681	5.353955575	1.813608113	0.122773417
151310	F943	L	F	96	LF	0.649305556	0.264771879	0.682618036	0.398236229	2.708579772	1.394828152	0.111356984
151310	F943	L	F	96	RF	0.669736842	0.236830818	0.828103466	0.53884723	3.035890828	1.806971902	0.112056668
151310	F943	L	F	96	LH	0.682142857	0.221516543	0.866304859	0.572886541	3.364704349	1.641813909	0.127242576
151310	F943	L	F	96	RH	0.59375	0.28598853	0.660522567	0.39196577	2.297938486	1.641813909	0.122822985
152686	F1158	L	F	1	LF	0.910447761	0.075735936	0.386959415	0.267456757	12.02134431	1.182427309	0.234193577
152686	F1158	L	F	1	RF	0.86440678	0.086005215	0.351036542	0.330270857	10.05063213	1.576569746	0.064347588
152686	F1158	L	F	1	LH	0.416666667	0.422858975	-0.0091897	-0.0039602	0.985356091	1.379498528	0.016043903
152686	F1158	L	F	1	RH	0.866666667	0.084571795	0.293422632	0.266582852	10.24770335	1.576569746	0
152686	F1158	L	F	2	LF	0.834248456	0.147449676	0.710156666	0.595412301	5.83784933	1.166560236	0.190527707
152686	F1158	L	F	2	RF	0.708578638	0.162306583	0.688272002	0.433776357	4.412955828	1.879006987	0.212136776

152686	F1158	L	F	2	LH	0.809090909	0.154476025	0.691220964	0.578555654	5.388783997	1.259402193	0.081283128
152686	F1158	L	F	2	RH	0.733500717	0.148651674	0.676636601	0.511584706	4.954863118	1.801309484	0.084809112
152686	F1158	L	F	4	LF	0.759294872	0.15804754	0.957568219	0.754093232	4.811328	1.521384727	0.147667149
152686	F1158	L	F	4	RF	0.743990385	0.177810071	0.92331909	0.608314094	4.257063304	1.442589328	0.108133712
152686	F1158	L	F	4	LH	0.704100367	0.154837644	0.967351593	0.665283156	4.572244899	1.926149344	0.139422093
152686	F1158	L	F	4	RH	0.705357143	0.167345047	0.888195397	0.6393542	4.254366398	1.763164735	0.210508991
152686	F1158	L	F	6	LF	0.725735294	0.165045283	0.911819931	0.589052462	4.44230793	1.648771907	0.277043724
152686	F1158	L	F	6	RF	0.685855263	0.174605397	1.002538036	0.60069119	3.95089506	1.810710283	0.219986045
152686	F1158	L	F	6	LH	0.716517857	0.181510226	1.061205801	0.630240034	3.952294496	1.56430413	0.155285035
152686	F1158	L	F	6	RH	0.673809524	0.209645453	0.957501714	0.688484262	3.211676602	1.567103002	0.163436131
152686	F1158	L	F	8	LF	0.733467742	0.18582431	0.924592081	0.656966392	4.015664789	1.467877238	0.191565249
152686	F1158	L	F	8	RF	0.678947368	0.193566812	0.995336267	0.685255014	3.55132297	1.700048148	0.20230431
152686	F1158	L	F	8	LH	0.663851351	0.189029973	1.017837899	0.642390015	3.552846084	1.773376815	0.174513137
152686	F1158	L	F	8	RH	0.66136725	0.182860199	0.967291082	0.625280649	3.623128525	1.851274823	0.183532951
152686	F1158	L	F	24	LF	0.831463906	0.112426961	0.86170965	0.678132569	7.393860725	1.506037926	0.112815091
152686	F1158	L	F	24	RF	0.795870113	0.118144723	0.996043172	0.748348705	6.796106743	1.736022154	0.118454596
152686	F1158	L	F	24	LH	0.75	0.132818438	0.922555707	0.684586774	5.660751813	1.886917271	0.187920505
152686	F1158	L	F	24	RH	0.742924528	0.126242459	0.828451109	0.577808738	5.884909557	2.036355767	0.156995107
152686	F1158	L	F	26	LF	0.730243902	0.216421398	0.708999694	0.500337792	3.637653072	1.263144122	0.14848866
152686	F1158	L	F	26	RF	0.672844828	0.199985439	0.846900333	0.567242331	3.490064827	1.634996081	0.179963794
152686	F1158	L	F	26	LH	0.641409266	0.21095653	0.830888038	0.540936866	3.11926063	1.709052144	0.216648414
152686	F1158	L	F	26	RH	0.650641026	0.22623066	0.735258847	0.452402701	3.117688987	1.560416136	0.214756709
152686	F1158	L	F	28	LF	0.831632653	0.127286415	0.864357404	0.711824658	6.539169219	1.353439353	0.080974683
152686	F1158	L	F	28	RF	0.826669112	0.128132589	0.856951219	0.729954167	6.540045762	1.352913427	0.131041714
152686	F1158	L	F	28	LH	0.751933189	0.117294537	0.924638141	0.724837718	6.46479625	2.104356689	0.185839542
152686	F1158	L	F	28	RH	0.74	0.133046388	0.819652065	0.581860889	5.561276189	1.954909518	0.202464826
154983	F1571	L	F	4	LF	0.786656891	0.235514608	0.909437876	0.624603294	3.400132206	1.003702159	0.208158072
154983	F1571	L	F	4	RF	0.68275076	0.171248885	1.210437874	0.768711823	4.431610645	1.74730682	0.263165739
154983	F1571	L	F	4	LH	0.72974537	0.2065963	0.924801073	0.782699199	3.56574827	1.328263773	0.131650409
154983	F1571	L	F	4	RH	0.710084034	0.322165294	0.645237464	0.346269251	2.240819755	0.912555983	0.072946841
154983	F1571	L	F	6	LF	0.699324324	0.181664135	1.0860502	0.782985133	3.896201525	1.736787805	0.182486864
154983	F1571	L	F	6	RF	0.649380805	0.160958078	0.715420301	0.57092911	4.058199069	2.166025741	0.147400167
154983	F1571	L	F	6	LH	0.639634146	0.214070824	0.630499117	0.348400749	3.69232785	1.56156622	0.044735705

154983	F1571	L	F	6	RH	0.733333333	0.159649794	1.189525284	0.657723161	4.660454583	1.646973005	0.167907017
154983	F1571	L	F	8	LF	0.713031915	0.145810016	0.877481424	0.651730727	5.006850463	1.901344193	0.233789146
154983	F1571	L	F	8	RF	0.716905901	0.260892454	0.599148299	0.38594865	2.933586345	1.188797403	0.154685273
154983	F1571	L	F	8	LH	0.670045045	0.216149895	0.73632277	0.428366918	3.328632728	1.508492764	0.122310289
154983	F1571	L	F	8	RH	0.805429864	0.142606545	0.939024062	0.694710302	5.7172023	1.43094679	0.119861188
159811	F1541	L	M	1	LF	0.739417989	0.121199064	0.577908569	0.473554695	6.183732013	2.177521624	0.109183778
159811	F1541	L	M	1	RF	0.824532225	0.121251952	0.489887064	0.336270765	7.473069011	1.397120601	0.171146259
159811	F1541	L	M	1	LH	0.772032694	0.110791828	0.652866717	0.410781985	7.392122871	2.091207992	0.222809787
159811	F1541	L	M	1	RH	0.73170045	0.137033694	0.517881005	0.398790477	5.561644942	1.841212915	0.172796491
159811	F1541	L	M	2	LF	0.698529412	0.162401668	0.718553737	0.478568496	4.432914196	1.84823484	0.204928135
159811	F1541	L	M	2	RF	0.798868636	0.15651785	0.781670074	0.607741537	5.151586031	1.286415451	0.137845805
159811	F1541	L	M	2	LH	0.695959596	0.163656079	0.716422542	0.496507419	4.434888938	1.84823484	0.155374361
159811	F1541	L	M	2	RH	0.761363636	0.218324308	0.615787762	0.480923746	3.766009563	1.20107763	0.057178469
159811	F1541	L	M	4	LF	0.80026455	0.189035917	0.554251214	0.467357879	4.417658625	1.124063547	0.127450863
159811	F1541	L	M	4	RF	0.720833333	0.192754599	0.600437375	0.458555385	3.934581896	1.447073312	0.15840032
159811	F1541	L	M	4	LH	0.728937729	0.190661884	0.61348871	0.448441552	3.936019813	1.446354354	0.173599306
159811	F1541	L	M	4	RH	0.763888889	0.19841046	0.585153616	0.42205881	4.254715825	1.284849471	0.112960938
159811	F1541	L	M	6	LF	0.782091097	0.127174249	0.882986685	0.750925294	6.179415363	1.701016423	0.256592765
159811	F1541	L	M	6	RF	0.814379699	0.098836863	0.769761092	0.735800726	8.237928527	1.880790759	0.15471167
159811	F1541	L	M	6	LH	0.829715762	0.126966059	0.633954048	0.535840115	6.536684112	1.343063697	0.143397288
159811	F1541	L	M	6	RH	0.745031056	0.140485894	0.769842825	0.637781435	5.463737904	1.790675599	0.109402508
159811	F1541	L	M	8	LF	0.708165612	0.149301497	0.990342325	0.71890034	4.769814211	2.019792046	0.110081452
159811	F1541	L	M	8	RF	0.774016763	0.159401939	0.81769516	0.657973606	5.090022938	1.375240741	0.098019731
159811	F1541	L	M	8	LH	0.712404712	0.162858734	0.778174212	0.618567484	4.365304621	1.780209647	0.140981399
159811	F1541	L	M	8	RH	0.591557018	0.145734204	1.015792885	0.611513511	4.122047687	2.829270542	0.091971533
159811	F1541	L	M	24	LF	0.735294118	0.187502696	0.939501797	0.764251028	3.921363915	1.411897729	0.149363658
159811	F1541	L	M	24	RF	0.727339181	0.172421209	0.79626083	0.600664068	4.234629189	1.569062366	0.097660852
159811	F1541	L	M	24	LH	0.736702128	0.167400126	0.7407447	0.572695259	4.548198462	1.646656685	0.137874144
159811	F1541	L	M	24	RH	0.657142857	0.182145477	0.881227455	0.583626373	3.607642642	1.882479639	0.203938834
159811	F1541	L	M	26	LF	0.794736842	0.135754826	0.80692276	0.641378073	6.056895252	1.573771139	0.082932371
159811	F1541	L	M	26	RF	0.829463364	0.146491359	0.793458609	0.609890755	5.820475642	1.17855866	0.099846254
159811	F1541	L	M	26	LH	0.72913257	0.14957275	0.822680076	0.573320838	4.954193638	1.804881665	0.14068733
159811	F1541	L	M	26	RH	0.704268293	0.131029016	0.859217494	0.612673978	5.668761551	2.277720885	0.180239012

159811	F1541	L	M	28	LF	0.692451072	0.19373462	1.10073686	0.822814204	3.658188347	1.588761638	0.102886484
159811	F1541	L	M	28	RF	0.700959024	0.186688518	0.981399085	0.766422771	3.817064511	1.587752494	0.099988892
159811	F1541	L	M	28	LH	0.720825274	0.162513501	1.003670713	0.779150776	4.61346362	1.748646947	0.149738206
159811	F1541	L	M	28	RH	0.545185811	0.183495753	1.119697502	0.644073666	3.022683692	2.461066822	0.150228474
159811	F1541	L	M	96	LF	0.730952381	0.169981165	1.164410217	0.874302872	4.308188762	1.611585	0.128330887
159811	F1541	L	M	96	RF	0.732936508	0.183092727	1.124999131	0.80033851	4.000327017	1.465156783	0.148367207
159811	F1541	L	M	96	LH	0.568303571	0.194235463	1.111434587	0.651676428	2.924686575	2.229184154	0.144832516
159811	F1541	L	M	96	RH	0.715686275	0.185855987	1.114538656	0.741477025	3.846396145	1.543060051	0.107115054
159828	F1546	L	F	0	LF	0.736689815	0.200090786	0.749100782	0.571682067	3.752421956	1.280256768	0.412416449
159828	F1546	L	F	0	RF	0.785615171	0.126059381	0.533000863	0.341575269	6.227017872	1.706928	0.152774214
159828	F1546	L	F	0	LH	0.833030303	0.17214359	0.796091551	0.730656302	7.159958065	1.108664599	0.031043336
159828	F1546	L	F	0	RH	0.765625	0.158144646	0.544281227	0.484690039	4.947004176	1.535092758	0.10736332
159828	F1546	L	F	1	LF	0.586845466	0.220421591	0.979168381	0.57871514	2.693625456	1.862131177	0.242011255
159828	F1546	L	F	1	RF	0.706233422	0.224934185	1.000705681	0.698800085	3.18123851	1.295600013	0.200366272
159828	F1546	L	F	1	LH	0.675521822	0.282786492	0.840023054	0.559412671	2.61940881	1.314405869	0.117119781
159828	F1546	L	F	1	RH	0.614058355	0.224934185	0.947331177	0.552068188	2.777245031	1.699593492	0.173507168
159828	F1546	L	F	2	LF	0.782938076	0.188635801	0.656941065	0.471620732	6.081277638	1.196175682	0.177265359
159828	F1546	L	F	2	RF	0.744306418	0.195467606	0.916389972	0.502603367	5.842647999	1.355598495	0.132713955
159828	F1546	L	F	2	LH	0.755232558	0.230293419	0.483509813	0.306598069	3.997680281	1.035743706	0.158827022
159828	F1546	L	F	2	RH	0.773333333	0.191854173	0.835332979	0.536439759	6.403150751	1.276391669	0.186881667
159828	F1546	L	F	4	LF	0.760025063	0.175565337	0.793937484	0.52357484	5.135914665	1.288201563	0.15097912
159828	F1546	L	F	4	RF	0.531484258	0.257594306	0.726466388	0.379817294	2.201314147	1.73784766	0.084308856
159828	F1546	L	F	4	LH	0.594827586	0.44518741	0.497923948	0.325637828	1.898478885	1.061459017	0.100626701
159828	F1546	L	F	4	RH	0.727889151	0.165292592	0.834931899	0.519350927	4.984497034	1.442690392	0.123846099
159828	F1546	L	F	6	LF	0.742424242	0.200808686	0.593880204	0.50680486	3.714175235	1.271389354	0.13668476
159828	F1546	L	F	6	RF	0.709022556	0.173412796	0.717577285	0.490559765	4.126540468	1.657474918	0.089433517
159828	F1546	L	F	6	LH	0.736352357	0.223578208	0.548325774	0.366395343	3.330279643	1.188916307	0.077356253
159828	F1546	L	F	6	RH	0.728110599	0.251552799	0.495285797	0.359037598	3.000387456	1.10644326	0.140100759
159828	F1546	L	F	8	LF	0.68131257	0.216457495	0.954895142	0.676161624	3.153979246	1.467516729	0.183231249
159828	F1546	L	F	8	RF	0.707142857	0.224158418	1.019849067	0.744978706	3.160655766	1.311167173	0.124207563
159828	F1546	L	F	8	LH	0.636363636	0.206630312	1.070415501	0.686234458	3.082480988	1.775764828	0.179801858
159828	F1546	L	F	8	RH	0.638095238	0.224158418	1.020824242	0.622989127	2.852407667	1.619415272	0.162159535
159828	F1546	L	F	24	LF	0.751390434	0.219452568	0.837901859	0.605909334	3.422092047	1.135410812	0.08708519

159828	F1546	L	F	24	RF	0.733036707	0.219452568	0.964857345	0.687443533	3.341322918	1.216179942	0.130735195
159828	F1546	L	F	24	LH	0.640625	0.205651215	1.044956575	0.670971622	3.11487514	1.749844628	0.210782746
159828	F1546	L	F	24	RH	0.626152074	0.223420259	0.884292512	0.525064399	2.807658232	1.67224742	0.15374354
159828	F1546	L	F	26	LF	0.69047619	0.18635858	0.996931817	0.665333634	3.825581634	1.684221747	0.14518852
159828	F1546	L	F	26	RF	0.751754386	0.194582349	1.097891133	0.822857406	3.904185894	1.300858727	0.095875798
159828	F1546	L	F	26	LH	0.69047619	0.201983741	1.065631868	0.735593674	3.444633184	1.531842367	0.153416901
159828	F1546	L	F	26	RH	0.68744551	0.193424741	1.04699709	0.691833253	3.597012564	1.608836914	0.136984808
159828	F1546	L	F	28	LF	0.706293706	0.274261963	1.116201617	0.773626739	2.604650433	1.071595197	0.114197498
159828	F1546	L	F	28	RF	0.6525	0.266789336	1.201267183	0.802969505	2.450462928	1.300671501	0.147298412
159828	F1546	L	F	28	LH	0.577380952	0.291776459	1.102898571	0.62069256	1.991207843	1.454859006	0.112580184
159828	F1546	L	F	28	RH	0.59965035	0.274261963	1.153622393	0.683934307	2.222489101	1.453756529	0.155819762
159828	F1546	L	F	96	LF	0.621428571	0.279954685	1.307112874	0.813859275	2.276119733	1.368822305	0.165704792
159828	F1546	L	F	96	RF	0.662640902	0.26356505	1.339476895	0.924513808	2.514758333	1.290588798	0.121465808
159828	F1546	L	F	96	LH	0.557165862	0.26356505	1.310612527	0.738160753	2.127528882	1.677818249	0.095106243
159828	F1546	L	F	96	RH	0.557692308	0.289199038	1.295225357	0.760312841	1.97106187	1.525289317	0.126033975
160446	F1546	L	M	0	LF	0.796296296	0.09455397	0.610401386	0.248643445	8.421606125	2.154364358	0.352713996
160446	F1546	L	M	0	RF	0.674418605	0.118742195	0.334626097	0.571157239	5.679687852	2.741918273	0.126615285
160446	F1546	L	M	0	LH	0.939393939	0.154724679	0.497417172	0.551680869	6.071390463	0.39170261	0.018087899
160446	F1546	L	M	0	RH	0.85483871	0.082353458	0.731397149	0.604781864	10.38011918	1.762661747	0.117571332
160446	F1546	L	M	1	LF	0.83765653	0.125313137	0.694970008	0.548978509	6.808350028	1.269632137	0.172577107
160446	F1546	L	M	1	RF	0.784038756	0.135540625	0.644212846	0.530137716	6.048155544	1.614539901	0.183887619
160446	F1546	L	M	1	LH	0.81970339	0.123914752	0.727761186	0.626411691	6.988727902	1.442086019	0.16476215
160446	F1546	L	M	1	RH	0.806055287	0.124261132	0.712623717	0.540063001	6.641178808	1.523030298	0.183887619
160446	F1546	L	M	2	LF	0.808333333	0.190607762	0.818442473	0.673290376	4.247883576	1.041750092	0.141910293
160446	F1546	L	M	2	RF	0.79984051	0.176609871	0.807295029	0.616710805	4.568790418	1.12201542	0.093544221
160446	F1546	L	M	2	LH	0.706656347	0.17382852	0.826497377	0.529185372	4.087816331	1.683100365	0.150953105
160446	F1546	L	M	2	RH	0.723484848	0.191995957	0.765530033	0.576917503	3.766986724	1.442767792	0.148016679
160446	F1546	L	M	4	LF	0.857246377	0.168971346	0.636287438	0.551006856	5.278206976	0.892680767	0.108625124
160446	F1546	L	M	4	RF	0.798757764	0.154459462	0.648901107	0.560172455	5.279592625	1.301467985	0.077262208
160446	F1546	L	M	4	LH	0.825543478	0.143575549	0.689791512	0.559245751	5.771800064	1.219433412	0.130350147
160446	F1546	L	M	4	RH	0.755208333	0.159814557	0.660896734	0.483061642	4.951454332	1.544800408	0.071562165
160446	F1546	L	M	6	LF	0.775689223	0.152133377	0.909823432	0.710057123	5.114842355	1.486792177	0.154167462
160446	F1546	L	M	6	RF	0.783783784	0.163928624	0.925268762	0.758773245	4.78242978	1.319290974	0.135382581

160446	F1546	L	M	6	LH	0.746794872	0.162103878	0.936341409	0.705266963	4.62010824	1.567952947	0.125636646
160446	F1546	L	M	6	RH	0.722996516	0.146202107	1.003285892	0.67792276	4.947341151	1.897775691	0.087359901
160446	F1546	L	M	8	LF	0.825	0.170798824	0.746090492	0.665413063	4.858660228	1.019424062	0.132515329
160446	F1546	L	M	8	RF	0.812695435	0.159567512	0.755390618	0.638836267	5.095928628	1.176212699	0.181487087
160446	F1546	L	M	8	LH	0.719512195	0.155563545	0.731860422	0.530151989	4.624966876	1.803367248	0.138287193
160446	F1546	L	M	8	RH	0.660433604	0.148623334	0.835306907	0.538560683	4.466390715	2.274329001	0.256323345
160446	F1546	L	M	24	LF	0.753378378	0.161347807	0.686047615	0.564642603	4.714044426	1.545961042	0.094989457
160446	F1546	L	M	24	RF	0.844180916	0.153936439	0.689979135	0.593990992	5.648648238	1.002497835	0.080812442
160446	F1546	L	M	24	LH	0.775195969	0.154377118	0.704779318	0.534192641	5.103118211	1.467732921	0.105748734
160446	F1546	L	M	24	RH	0.78125	0.14864146	0.718941598	0.56055266	5.335735754	1.465666101	0.11105905
160446	F1546	L	M	26	LF	0.791858679	0.18142402	0.726039904	0.566086981	4.498420725	1.161968306	0.069609344
160446	F1546	L	M	26	RF	0.718070652	0.171519589	0.843621048	0.620807666	4.343610674	1.706482259	0.078243696
160446	F1546	L	M	26	LH	0.750187547	0.179656063	0.782858392	0.60087095	4.345396524	1.393290458	0.102702077
160446	F1546	L	M	26	RH	0.634199134	0.17503603	0.789303513	0.507309729	3.63892912	2.174484112	0.139315002
160446	F1546	L	M	28	LF	0.809162821	0.163618647	0.763339325	0.627733047	4.946477385	1.175430038	0.066032918
160446	F1546	L	M	28	RF	0.792510121	0.165402827	0.744861699	0.573742324	4.791087341	1.254792512	0.091684222
160446	F1546	L	M	28	LH	0.7875	0.15919532	0.77656504	0.60836959	4.948144837	1.334154986	0.068735501
160446	F1546	L	M	28	RH	0.759935897	0.161214617	0.783891015	0.611681627	4.71339232	1.489545029	0.085947045
160446	F1546	L	M	96	LF	0.788537549	0.14567158	0.799066895	0.698086573	5.428001491	1.449656964	0.082937743
160446	F1546	L	M	96	RF	0.85	0.130978238	0.722246926	0.618071679	6.49090835	1.147351325	0.084869379
160446	F1546	L	M	96	LH	0.803784014	0.135072852	0.746448088	0.577636949	5.964292042	1.446432216	0.065100763
160446	F1546	L	M	96	RH	0.728841608	0.142369601	0.721939218	0.552828524	5.119246356	1.904727797	0.104869376
160639	F1546	L	F	0	LF	0.85555402	0.074997399	0.570980777	0.415971564	16.86873072	2.268528068	0.271073251
160639	F1546	L	F	0	RF	0.841106762	0.061152693	0.892546785	0.709435476	17.60053031	2.229525788	0.29534033
160639	F1546	L	F	0	LH	0.824786325	0.124632487	0.503310128	0.425401683	6.737330215	1.487975621	0.063634503
160639	F1546	L	F	0	RH	0.876902744	0.064100609	0.778965739	0.711496966	23.0156446	1.777026325	0.03529884
160639	F1546	L	F	1	LF	0.753493789	0.170534008	0.771447164	0.522550106	5.38861647	1.706549618	0.213998979
160639	F1546	L	F	1	RF	0.620634921	0.233934238	0.671627575	0.406325108	3.05364114	1.707050381	0.175368474
160639	F1546	L	F	1	LH	0.707264957	0.225770585	0.71400857	0.494337079	3.592828283	1.52682057	0.084051445
160639	F1546	L	F	1	RH	0.803069054	0.17541119	0.76063812	0.556885555	5.388866852	1.257602571	0.106835014
160639	F1546	L	F	2	LF	0.736719478	0.179195444	0.748902999	0.60487986	4.172883357	1.457426807	0.071175857
160639	F1546	L	F	2	RF	0.83733562	0.144645531	0.678968047	0.534413835	6.297637243	1.110354077	0.129888211
160639	F1546	L	F	2	LH	0.777777778	0.118520461	1.101286104	0.701727097	8.329745515	1.790393701	0.110674124

160639	F1546	L	F	2	RH	0.743697479	0.119252485	0.887500344	0.664127036	8.251205736	2.137466431	0.074632218
160639	F1546	L	F	4	LF	0.812118902	0.156491831	0.982032132	0.745131383	5.249838947	1.246960213	0.208700787
160639	F1546	L	F	4	RF	0.663709677	0.230969176	0.762439355	0.50402342	3.02055182	1.510715111	0.17087736
160639	F1546	L	F	4	LH	0.732078853	0.168814191	1.296521572	0.85880953	4.359529538	1.601854215	0.167583796
160639	F1546	L	F	4	RH	0.694404591	0.151299305	1.117001305	0.704056719	4.626798041	2.047008919	0.122788144
160639	F1546	L	F	6	LF	0.735042735	0.223326357	1.001600693	0.710606306	3.296356225	1.183116617	0.121955775
160639	F1546	L	F	6	RF	0.633289125	0.215776395	1.127849312	0.735111181	2.958411467	1.690219732	0.098296497
160639	F1546	L	F	6	LH	0.693939394	0.207975987	1.128393837	0.828128952	3.465886536	1.436606182	0.17945106
160639	F1546	L	F	6	RH	0.606138107	0.215637748	1.090566059	0.63322601	2.958659436	1.859502074	0.19992238
160639	F1546	L	F	8	LF	0.684065934	0.257263652	1.156169224	0.814377584	2.683488865	1.260247445	0.154716947
160639	F1546	L	F	8	RF	0.636904762	0.217165578	1.20022569	0.816977969	2.931415521	1.678039464	0.097646985
160639	F1546	L	F	8	LH	0.647586207	0.222053693	1.19759271	0.708949937	2.929125059	1.59081632	0.130853098
160639	F1546	L	F	8	RH	0.619162641	0.240018898	1.172419131	0.75039424	2.593975258	1.59081632	0.182884552
160639	F1546	L	F	24	LF	0.698606272	0.140841031	0.960073148	0.664856192	5.02387564	2.150981744	0.136638965
160639	F1546	L	F	24	RF	0.752439024	0.155120111	0.947223406	0.740167065	4.86251391	1.588323506	0.195507539
160639	F1546	L	F	24	LH	0.70963271	0.165445317	0.841915109	0.61383442	4.304071304	1.749685236	0.134064767
160639	F1546	L	F	24	RH	0.704759107	0.153354794	0.90534859	0.625073144	4.709583444	1.90893915	0.116734147
160639	F1546	L	F	26	LF	0.720394737	0.182986843	0.95345236	0.677078119	4.025429097	1.491451208	0.172809624
160639	F1546	L	F	26	RF	0.711382114	0.165789233	1.001474547	0.763491577	4.336756404	1.730366618	0.128974124
160639	F1546	L	F	26	LH	0.705627706	0.172077491	0.873652003	0.703410462	4.179286107	1.733979904	0.100631281
160639	F1546	L	F	26	RH	0.672844828	0.189240129	0.946705989	0.601091925	3.710488504	1.732173261	0.136184141
160639	F1546	L	F	28	LF	0.769548872	0.174428476	0.857027506	0.637737857	4.406074088	1.330646735	0.102909034
160639	F1546	L	F	28	RF	0.750980392	0.199333431	0.923857306	0.706908761	3.772056634	1.255708719	0.120453574
160639	F1546	L	F	28	LH	0.627005348	0.189936994	0.985736964	0.563667136	3.302706638	1.967129427	0.071892912
160639	F1546	L	F	28	RH	0.691941392	0.172541239	0.92105415	0.654106228	4.00919687	1.814788158	0.121179175
160639	F1546	L	F	96	LF	0.741578947	0.222100306	0.96026315	0.718232086	3.53138488	1.200292525	0.11364929
160639	F1546	L	F	96	RF	0.792471686	0.129485839	0.974933158	0.736009049	7.071885295	1.645280945	0.107750842
160639	F1546	L	F	96	LH	0.825328947	0.128949875	0.89247478	0.685592438	7.443068366	1.347903222	0.105638844
160639	F1546	L	F	96	RH	0.640977444	0.207583355	0.992545641	0.645829047	3.231850831	1.721242621	0.123467276
151303	F943	N	F	0	LF	0.871128871	0.090475097	0.548336498	0.452371675	11.14582463	1.519309339	0.268149275
151303	F943	N	F	0	RF	0.886303543	0.07537815	0.678434664	0.61784989	11.76969361	1.519309339	0.28988081
151303	F943	N	F	0	LH	0.743421053	0.177762142	0.263373706	0.211477927	4.695338495	1.602307809	0.05161051
151303	F943	N	F	0	RH	0.89039548	0.070444773	0.628381712	0.584066323	13.03286093	1.497869493	0.043036678

151303	F943	N	F	1	LF	0.733225108	0.122839349	0.898124341	0.669757221	5.967907869	2.182134516	0.170196885
151303	F943	N	F	1	RF	0.817859953	0.149273317	0.678214133	0.564583597	5.580907017	1.23067718	0.160687584
151303	F943	N	F	1	LH	0.791304348	0.123434153	0.848406767	0.626058136	6.446644937	1.707408648	0.246966074
151303	F943	N	F	1	RH	0.75297619	0.242063021	0.756858518	0.472029484	4.519663072	1.320407795	0.246966074
151303	F943	N	F	2	LF	0.73024948	0.186864538	1.025355977	0.574171707	3.987661362	1.473573301	0.268149275
151303	F943	N	F	2	RF	0.67	0.209841812	0.853374505	0.574171707	3.211678765	1.569579083	0.28988081
151303	F943	N	F	2	LH	0.706923077	0.225056656	0.77626952	0.545982694	3.145029462	1.299175625	0.05161051
151303	F943	N	F	2	RH	0.67281106	0.227784046	0.802124329	0.58013475	3.103930391	1.401052703	0.043036678
151303	F943	N	F	4	LF	0.768939394	0.16262533	0.81692641	0.642997026	4.767382241	1.448422649	0.094595373
151303	F943	N	F	4	RF	0.759324009	0.202246387	0.771278551	0.581774284	3.833437174	1.191183858	0.11326015
151303	F943	N	F	4	LH	0.721153846	0.194883814	0.800036248	0.568276712	3.834760579	1.447099244	0.09787338
151303	F943	N	F	4	RH	0.678125	0.189821284	0.780469874	0.487037847	3.573551574	1.70301463	0.128207995
151303	F943	N	F	6	LF	0.735966736	0.151039343	0.778875942	0.629569236	4.87361809	1.747274677	0.121857346
151303	F943	N	F	6	RF	0.760981912	0.130563508	0.750244911	0.525496252	5.846143217	1.828583399	0.106388975
151303	F943	N	F	6	LH	0.83507371	0.143044047	0.65126596	0.555246224	5.946984236	1.127329652	0.094392479
151303	F943	N	F	6	RH	0.804993252	0.149044211	0.715038809	0.596670003	5.400535015	1.309479393	0.081449106
151303	F943	N	F	8	LF	0.66733871	0.186680811	0.887400779	0.628505728	3.571304957	1.788868596	0.099606695
151303	F943	N	F	8	RF	0.741666667	0.189755197	0.850063747	0.613974857	3.911582387	1.36110972	0.129704511
151303	F943	N	F	8	LH	0.754261364	0.180933188	0.896286532	0.658564189	4.167594489	1.362717779	0.108238583
151303	F943	N	F	8	RH	0.593434343	0.170631733	0.987291422	0.64506527	3.48543157	2.381942011	0.108352139
151303	F943	N	F	24	LF	0.806063123	0.177504665	0.71123092	0.591601348	4.721455151	1.158931995	0.088363382
151303	F943	N	F	24	RF	0.788888889	0.159034899	0.711562914	0.573886499	4.974177374	1.327041871	0.096600137
151303	F943	N	F	24	LH	0.743589744	0.154582515	0.763727317	0.496598547	4.810526782	1.658802339	0.108071455
151303	F943	N	F	24	RH	0.757264957	0.144207755	0.789190086	0.618534967	5.30259338	1.66103198	0.085819141
151303	F943	N	F	26	LF	0.812770563	0.117402691	0.805859416	0.606965506	7.415887718	1.578703407	0.07359919
151303	F943	N	F	26	RF	0.796560197	0.136198816	0.718174962	0.598795918	6.164056269	1.496281049	0.144796747
151303	F943	N	F	26	LH	0.872493986	0.121955567	0.57332638	0.574404297	7.411661511	0.994703228	0.059475092
151303	F943	N	F	26	RH	0.836485918	0.126661585	0.766674145	0.639046882	6.996732254	1.331436335	0.084936938
151303	F943	N	F	28	LF	0.815714286	0.147650418	0.889012429	0.713353813	5.768388377	1.234300447	0.072126838
151303	F943	N	F	28	RF	0.81022409	0.161562625	0.781790284	0.710694871	5.02072301	1.231885073	0.083390376
151303	F943	N	F	28	LH	0.81022409	0.164612721	0.794698367	0.581760999	4.774184971	1.315406294	0.086014583
151303	F943	N	F	28	RH	0.788555692	0.150606322	0.838363523	0.692904394	5.270481549	1.398927515	0.077566929
151303	F943	N	F	96	LF	0.782142857	0.141843491	0.585824786	0.442575347	6.292521862	1.538199486	0.063187696

151303	F943	N	F	96	RF	0.765037594	0.144397615	0.55495203	0.413915459	5.906149786	1.666990178	0.078802942
151303	F943	N	F	96	LH	0.637435897	0.128157386	0.683934245	0.376694186	5.523422119	2.688812093	0.047925515
151303	F943	N	F	96	RH	0.720454545	0.137601894	0.574466691	0.425327773	6.037370084	1.920927153	0.031472725
151307	F943	N	F	0	LF	0.826573427	0.106754284	0.770316309	0.687412012	11.52190457	2.239818475	0.067687779
151307	F943	N	F	0	RF	0.839598643	0.079311064	0.977308996	0.947166677	10.5984403	2.049303933	0.304616603
151307	F943	N	F	0	LH	0.75	0.22695194	0.328988073	0.302989031	3.437119616	1.145706539	0.041947939
151307	F943	N	F	0	RH	0.879468599	0.04726052	1.391325834	1.123382604	19.69263114	2.354917925	0.027837985
151307	F943	N	F	1	LF	0.766470588	0.15581862	0.93472374	0.663799223	4.997905071	1.667824041	0.200152257
151307	F943	N	F	1	RF	0.763616558	0.178680509	0.847649268	0.709364595	4.842851797	1.348571621	0.190947362
151307	F943	N	F	1	LH	0.691964286	0.323322025	0.480363854	0.353231608	2.693166776	0.951792563	0.154118008
151307	F943	N	F	1	RH	0.731442206	0.145419217	0.956131411	0.675560241	5.078215235	1.823274962	0.068895366
151307	F943	N	F	2	LF	0.798582996	0.209743575	0.645072644	0.488825148	3.976848505	1.015100913	0.234182404
151307	F943	N	F	2	RF	0.712121212	0.217558317	0.725131858	0.545863652	3.351867105	1.317714389	0.124307245
151307	F943	N	F	2	LH	0.738284704	0.194476396	0.836475848	0.609284942	3.893293357	1.405220427	0.123958017
151307	F943	N	F	2	RH	0.696153846	0.190678068	0.719812602	0.438784448	3.738035729	1.636131423	0.027641664
151307	F943	N	F	4	LF	0.734006734	0.265776991	0.986084297	0.686803952	2.786102409	1.006069932	0.123333111
151307	F943	N	F	4	RF	0.663709677	0.265776991	1.097434165	0.76585304	2.631533684	1.315715982	0.075497345
151307	F943	N	F	4	LH	0.560606061	0.281458036	1.006382161	0.606554146	2.012343304	1.54751821	0.130886824
151307	F943	N	F	4	RH	0.674568966	0.24602027	1.137439626	0.703829748	2.786204129	1.315512542	0.196330236
151307	F943	N	F	6	LF	0.728365385	0.221599345	0.883521776	0.664005125	3.299497371	1.255000066	0.149392724
151307	F943	N	F	6	RF	0.736467236	0.240220478	0.709614314	0.55795826	3.064335512	1.101439422	0.211473025
151307	F943	N	F	6	LH	0.722222222	0.235653922	0.706182392	0.495440596	3.063130289	1.18063019	0.140077344
151307	F943	N	F	6	RH	0.775	0.219721202	0.871195217	0.680336294	3.538274901	1.02104343	0.098055808
151307	F943	N	F	8	LF	0.838709677	0.206803147	0.89067193	0.738933827	4.055594372	0.779921995	0.10624913
151307	F943	N	F	8	RF	0.740740741	0.23744065	0.663003255	0.554640494	3.119687979	1.091890792	0.121427576
151307	F943	N	F	8	LH	0.714285714	0.228960627	0.575375241	0.442108387	3.119687979	1.247875191	0.091070684
151307	F943	N	F	8	RH	0.742857143	0.183168501	0.961538934	0.74239998	4.055594372	1.40385959	0.156843951
151307	F943	N	F	24	LF	0.810129407	0.159362235	1.066767904	0.856121747	7.318325994	1.327428048	0.090846403
151307	F943	N	F	24	RF	0.783851976	0.151452281	0.940927744	0.689198407	7.238969978	1.47996173	0.088074705
151307	F943	N	F	24	LH	0.663580247	0.221002471	0.7474194	0.444358478	6.003254646	1.797385795	0.047375489
151307	F943	N	F	24	RH	0.833333333	0.221002471	0.850130954	0.65991911	6.781368931	1.019271509	0.081458928
151307	F943	N	F	26	LF	0.753320683	0.204544232	0.871918566	0.670567003	3.692478468	1.205874844	0.107845199
151307	F943	N	F	26	RF	0.709663866	0.192343011	0.857011587	0.661332349	3.69350495	1.507001394	0.065651793

151307	F943	N	F	26	LH	0.674725275	0.179850789	0.797846532	0.599074185	3.769043208	1.808812266	0.164103077
151307	F943	N	F	26	RH	0.680555556	0.192641468	0.932612623	0.628058259	3.541744112	1.65807791	0.175726644
151307	F943	N	F	28	LF	0.730769231	0.218339067	0.71301005	0.551416705	3.576795631	1.226852558	0.044100178
151307	F943	N	F	28	RF	0.82168784	0.211438747	0.706414546	0.607481413	3.995376265	0.872926102	0.093921336
151307	F943	N	F	28	LH	0.746928747	0.198835351	0.709406601	0.557313747	3.755430376	1.317053045	0.078457433
151307	F943	N	F	28	RH	0.728888889	0.217541573	0.770701994	0.57678895	3.806522996	1.306834521	0.084466816
151307	F943	N	F	96	LF	0.796551724	0.180284211	0.535711436	0.424895969	4.539532753	1.163534526	0.052959006
151307	F943	N	F	96	RF	0.782738095	0.165460071	0.540158754	0.435921862	4.773113709	1.291250632	0.060099972
151307	F943	N	F	96	LH	0.702978056	0.170732979	0.507643055	0.329581664	4.191346445	1.749672041	0.011544064
151307	F943	N	F	96	RH	0.648809524	0.177967637	0.506277568	0.318283711	3.724184534	1.983252996	0.020658209
152750	F1571	N	F	0	LF	0.727558605	0.103289585	0.557647575	0.216428467	7.951873192	2.502859619	0.653702956
152750	F1571	N	F	0	RF	0.67281106	0.219123706	1.380128491	0.351145927	3.933462112	1.570123053	0.263984545
152750	F1571	N	F	0	LH	0.892613934	0.047778388	1.536047054	1.179776481	18.80097347	2.312710096	0.294117967
152750	F1571	N	F	0	RH	0.659275237	0.056513828	0.216428467	1.036830421	13.95332429	4.999536073	0.229015886
152750	F1571	N	F	1	LF	0.801536098	0.112286208	1.031817484	0.87675262	9.415212467	1.94470903	0.303141197
152750	F1571	N	F	1	RF	0.669642857	0.427823841	0.608669928	0.400807444	13.94825111	1.68935341	0.12985982
152750	F1571	N	F	1	LH	0.871560535	0.107523181	0.814545066	0.623784997	15.1381733	1.86567131	0.220305882
152750	F1571	N	F	1	RH	0.884214368	0.061819124	1.414986698	1.068282049	16.76237507	1.66589882	0.149190428
152750	F1571	N	F	2	LF	0.68961039	0.204241811	0.865750681	0.645522911	3.379429217	1.517230596	0.147439923
152750	F1571	N	F	2	RF	0.817329332	0.192090273	0.727244438	0.616374319	4.377020979	0.938046962	0.116499213
152750	F1571	N	F	2	LH	0.726789168	0.230010364	0.582292319	0.456663015	3.509635058	1.427116131	0.027835042
152750	F1571	N	F	2	RH	0.742857143	0.222662453	0.748405066	0.576928072	3.376650157	1.152809147	0.127508042
152750	F1571	N	F	4	LF	0.803668478	0.178996798	0.843954786	0.634733072	4.719975958	1.119192602	0.242414953
152750	F1571	N	F	4	RF	0.818926975	0.132081111	0.764922967	0.679434217	6.434897522	1.421458681	0.166459591
152750	F1571	N	F	4	LH	0.864444444	0.190138623	0.634420818	0.547481527	5.81091921	0.825729719	0.149185677
152750	F1571	N	F	4	RH	0.820855615	0.168770953	0.957496042	0.74249076	5.171174276	1.1235942	0.149185677
152750	F1571	N	F	6	LF	0.727272727	0.185746398	0.916588165	0.754907147	3.915406895	1.468277586	0.246866247
152750	F1571	N	F	6	RF	0.923076923	0.078585015	0.978435576	0.806702535	11.74622068	0.978851724	0.085866524
152750	F1571	N	F	6	LH	0.333333333	0.510802594	0.4226247	0.153929175	0.652567816	1.305135632	0.160999726
152750	F1571	N	F	6	RH	0.65625	0.191550973	0.903397927	0.678867469	3.425981033	1.794561493	0.230766275
152750	F1571	N	F	8	LF	0.792207792	0.243780688	0.807478158	0.639769651	3.697419601	0.85276766	0.229477077
152750	F1571	N	F	8	RF	0.682685253	0.196227858	0.995861671	0.686653675	3.768169612	1.634576002	0.180218072
152750	F1571	N	F	8	LH	0.722978723	0.215665902	0.973279235	0.636538746	3.768378919	1.350529423	0.206858905

152750	F1571	N	F	8	RH	0.688393608	0.207165431	0.957938005	0.634796781	3.48349511	1.492238752	0.247760885
152750	F1571	N	F	24	LF	0.858080808	0.134572308	0.812780559	0.653288394	6.692903237	1.042786448	0.120529501
152750	F1571	N	F	24	RF	0.818274556	0.125969284	0.923012701	0.666170496	6.903609256	1.461512574	0.134854984
152750	F1571	N	F	24	LH	0.722635135	0.138822037	0.930695341	0.64267896	5.863508719	2.089601763	0.468769692
152750	F1571	N	F	24	RH	0.722635135	0.133011676	0.805766689	0.64267896	6.060785181	1.600640297	0.260994945
152750	F1571	N	F	26	LF	0.741414141	0.187045592	1.153106201	0.913546996	4.065954896	1.402508473	0.220042749
152750	F1571	N	F	26	RF	0.731900452	0.173175945	1.225429411	0.887527365	4.552968094	1.474864779	0.182789485
152750	F1571	N	F	26	LH	0.678571429	0.195534491	0.999114823	0.665537689	3.921870702	1.683135852	0.455283049
152750	F1571	N	F	26	RH	0.721062619	0.18462308	1.188713301	0.854181069	4.203754915	1.542822163	0.226325779
152750	F1571	N	F	28	LF	0.677142857	0.268599879	1.1373282	0.830537697	2.542711584	1.198166293	0.194241809
152750	F1571	N	F	28	RF	0.700892857	0.237588852	0.941065927	0.751856908	2.964135043	1.271355792	0.197303275
152750	F1571	N	F	28	LH	0.656451613	0.203304412	0.903855084	0.684823382	3.322997125	1.692779251	0.33667361
152750	F1571	N	F	28	RH	0.577380952	0.274582447	0.983738956	0.652388495	2.117745418	1.553485667	0.358532923
152750	F1571	N	F	96	LF	0.714338507	0.200689675	1.069174916	0.80889313	3.60775344	1.42994349	0.156952148
152750	F1571	N	F	96	RF	0.8012003	0.203659163	0.911811987	0.772442414	4.082686183	0.953981695	0.121816341
152750	F1571	N	F	96	LH	0.732446415	0.200689675	0.753181821	0.569378812	3.676630027	1.361066903	0.110231549
152750	F1571	N	F	96	RH	0.677156177	0.205298471	1.036174935	0.7596408	3.335334249	1.566667611	0.131685501
152776	F1571	N	M	0	LF	0.669824561	0.159713924	0.86703719	0.710322149	6.053807687	1.932278226	0.268214578
152776	F1571	N	M	0	RF	0.777283372	0.123491041	1.132850802	0.751283857	7.490152796	1.761294227	0.381666197
152776	F1571	N	M	0	LH	0.769230769	0.190999969	0.601983686	0.516981983	4.343271885	1.145731953	0.13321396
152776	F1571	N	M	0	RH	0.589285714	0.226264407	0.574782901	0.237218353	2.770278742	1.898141067	0.257089774
152776	F1571	N	M	1	LF	0.621212121	0.201218107	1.401365432	0.876269994	3.150491412	1.989476685	0.273675788
152776	F1571	N	M	1	RF	0.685461957	0.208960344	1.234566847	0.963106255	3.431101966	1.529220042	0.2297844
152776	F1571	N	M	1	LH	0.698051948	0.207326095	1.287245205	0.90739037	3.615204623	1.53367661	0.301533731
152776	F1571	N	M	1	RH	0.682010582	0.183302088	1.571003551	1.030598212	3.886902041	1.699952994	0.099258198
152776	F1571	N	M	2	LF	0.781682028	0.234751912	0.76999564	0.581384352	3.328239014	0.932226241	0.107296136
152776	F1571	N	M	2	RF	0.817307692	0.171003007	0.921070602	0.774431993	6.645580709	1.163223716	0.182518603
152776	F1571	N	M	2	LH	0.723276723	0.181429891	0.933626291	0.691687906	5.922050489	1.672482552	0.210605201
152776	F1571	N	M	2	RH	0.767482517	0.151556343	1.110534555	0.75996467	6.403432507	1.75319862	0.183961533
152776	F1571	N	M	4	LF	0.854897661	0.107018304	0.7014742	0.643413754	8.167780255	1.320774249	0.135213521
152776	F1571	N	M	4	RF	0.887627119	0.125769493	0.499221083	0.402441812	7.134657521	0.880867398	0.1351436
152776	F1571	N	M	4	LH	0.629129129	0.217691565	0.513528631	0.204404828	3.007435075	1.690875615	0.115820326
152776	F1571	N	M	4	RH	0.854893411	0.149925135	0.616665809	0.520332612	5.728272331	0.956995066	0.104805489

152776	F1571	N	M	6	LF	0.773529412	0.172308954	0.950971135	0.768988964	4.835878803	1.215567083	0.12103628
152776	F1571	N	M	6	RF	0.825974026	0.163102229	0.808987278	0.637652935	5.483611008	1.002629004	0.133474859
152776	F1571	N	M	6	LH	0.725	0.157896449	0.869741043	0.656598383	5.201477162	1.648577615	0.11016434
152776	F1571	N	M	6	RH	0.773660714	0.208233334	0.680933913	0.504171739	3.742650914	1.075391957	0.169454969
152776	F1571	N	M	8	LF	0.875	0.125896441	0.791230797	0.66729469	6.949629767	0.995321548	0.107227754
152776	F1571	N	M	8	RF	0.826061998	0.120168203	0.797167007	0.606836284	6.940819231	1.484171787	0.110728658
152776	F1571	N	M	8	LH	0.850490196	0.127710322	0.827470959	0.669171738	6.72250145	1.141600947	0.104534822
152776	F1571	N	M	8	RH	0.793764637	0.120656645	0.822322545	0.589618142	6.589437855	1.704692202	0.169926506
152776	F1571	N	M	24	LF	0.754310345	0.210101616	0.844248075	0.681629472	3.742633285	1.224835738	0.133537652
152776	F1571	N	M	24	RF	0.829545455	0.132321149	0.912819101	0.757932542	9.318936275	1.361087882	0.182635623
152776	F1571	N	M	24	LH	0.793122391	0.129072063	0.891560456	0.636403879	9.318675627	1.565466099	0.182630171
152776	F1571	N	M	24	RH	0.713541667	0.191275021	0.890289104	0.685317917	3.946490207	1.497340026	0.113902688
152776	F1571	N	M	26	LF	0.703703704	0.283913927	1.025474305	0.74576964	2.496391071	1.039520668	0.123452822
152776	F1571	N	M	26	RF	0.739583333	0.246359267	1.07352652	0.838552184	3.051893967	1.039038959	0.101354184
152776	F1571	N	M	26	LH	0.690625	0.232954928	1.091281637	0.740938702	2.982335678	1.315826989	0.135880562
152776	F1571	N	M	26	RH	0.641666667	0.270602416	1.04780742	0.760056309	2.4277962	1.316790407	0.154025736
152776	F1571	N	M	28	LF	0.712941176	0.25150094	1.143891524	0.822230793	2.911127988	1.178894952	0.130141091
152776	F1571	N	M	28	RF	0.733939394	0.254675666	1.196686473	0.905104861	2.983346773	1.036491731	0.147125904
152776	F1571	N	M	28	LH	0.621794872	0.249032014	1.220323433	0.771592378	2.566308861	1.521679729	0.157575199
152776	F1571	N	M	28	RH	0.646428571	0.232786023	1.288298144	0.807403149	2.842977902	1.521679729	0.14243153
152776	F1571	N	M	96	LF	0.776437848	0.133680014	0.920207505	0.713911354	5.967931457	1.661136447	0.071741751
152776	F1571	N	M	96	RF	0.820228867	0.147046582	0.978471602	0.822570039	5.832833771	1.19673882	0.0986334
152776	F1571	N	M	96	LH	0.694627193	0.14434798	0.937992888	0.680991656	4.844933946	2.124408171	0.069282718
152776	F1571	N	M	96	RH	0.713317847	0.151280421	0.902880408	0.641508328	5.098240773	1.797360036	0.057802495
154850	F998	N	F	0	LF	0.846590909	0.119771532	0.616092793	0.480759591	9.420877929	1.528104482	0.339171935
154850	F998	N	F	0	RF	0.701785714	0.160240016	0.726003978	0.371452071	4.895539921	2.009444749	0.196121048
154850	F998	N	F	0	LH	0.781818182	0.153697962	0.92353311	0.633801689	12.24507801	2.065500536	0.421313835
154850	F998	N	F	0	RH	0.497619048	0.255882961	0.542731441	0.333717252	2.124475973	2.010417967	0.232428481
154850	F998	N	F	1	LF	0.788461538	0.240613774	0.731508042	0.601536383	3.276895681	0.879142608	0.100910627
154850	F998	N	F	1	RF	0.77417795	0.17548359	0.461384666	0.322221027	8.233641787	1.038912882	0.093261743
154850	F998	N	F	1	LH	0.769090909	0.267295901	0.568497814	0.461397518	2.877257277	0.879103932	0.129798975
154850	F998	N	F	1	RH	0.598544974	0.227563613	0.743059004	0.449415489	2.637389147	1.758401243	0.156307303
154850	F998	N	F	2	LF	0.801388889	0.209947612	0.710385395	0.561722172	3.98909038	0.960336573	0.08202109

154850	F998	N	F	2	RF	0.812770563	0.238068139	0.71768453	0.581802811	3.98909038	0.812592485	0.094836884
154850	F998	N	F	2	LH	0.791891892	0.260677188	0.666421349	0.520012724	3.324241984	0.886464529	0.092273725
154850	F998	N	F	2	RH	0.745789896	0.195400619	0.878517315	0.643297516	3.98909038	1.329696793	0.117905315
154850	F998	N	F	4	LF	0.716920609	0.193066227	0.944903102	0.653516071	3.824530686	1.479263389	0.137225608
154850	F998	N	F	4	RF	0.770604396	0.18289078	0.791580517	0.661437261	4.606745281	1.259592687	0.123404672
154850	F998	N	F	4	LH	0.763640999	0.153573587	0.906524965	0.726427949	5.194646967	1.439850212	0.125989714
154850	F998	N	F	4	RH	0.804112554	0.18152116	0.733959919	0.600976106	4.489882239	1.052600882	0.157302498
154850	F998	N	F	6	LF	0.608225108	0.160350999	1.189442839	0.620408294	3.786683208	3.02245636	0.126831467
154850	F998	N	F	6	RF	0.681985294	0.212133547	0.867331512	0.610258597	3.214841941	1.499318139	0.172827499
154850	F998	N	F	6	LH	0.64202509	0.209714732	0.811442781	0.571109963	3.068351248	1.711993426	0.109336154
154850	F998	N	F	6	RH	0.726351351	0.203581806	0.860639478	0.634202739	3.570477545	1.352827446	0.119781896
154850	F998	N	F	8	LF	0.793144208	0.259423908	0.518709018	0.42415205	3.754793656	0.940770897	0.107722942
154850	F998	N	F	8	RF	0.793367347	0.141075568	0.864224635	0.657971071	5.643998167	1.454992084	0.118590835
154850	F998	N	F	8	LH	0.763888889	0.147372496	0.801517526	0.581368174	5.196723633	1.613755181	0.141954766
154850	F998	N	F	8	RH	0.70212766	0.145520012	0.801793352	0.634224745	4.820540717	2.05688475	0.088715801
154850	F998	N	F	24	LF	0.743727599	0.24589139	0.824193018	0.603450732	3.027239807	1.054359868	0.093559993
154850	F998	N	F	24	RF	0.692857143	0.219651516	0.825062218	0.59727875	3.166822909	1.407060926	0.098349909
154850	F998	N	F	24	LH	0.673809524	0.219651516	0.838599104	0.505644938	3.094535822	1.479348012	0.149444324
154850	F998	N	F	24	RH	0.669607843	0.22266168	0.880832192	0.626618114	3.024744272	1.479348012	0.12189186
154850	F998	N	F	26	LF	0.774603175	0.227564296	0.836368252	0.651390757	3.451807204	1.006609462	0.142174099
154850	F998	N	F	26	RF	0.734199134	0.204396957	0.860633525	0.655988147	3.597141259	1.297277571	0.133495296
154850	F998	N	F	26	LH	0.706677266	0.195917506	0.85054654	0.56459363	3.601164604	1.508573078	0.160212972
154850	F998	N	F	26	RH	0.680672269	0.201330691	0.893897843	0.650838384	3.381822407	1.58526345	0.161774396
154850	F998	N	F	28	LF	0.676190476	0.254650473	1.146213572	0.773606897	2.764387676	1.312769205	0.153908617
154850	F998	N	F	28	RF	0.69047619	0.254650473	1.018743414	0.703161648	2.83381231	1.243344571	0.126214686
154850	F998	N	F	28	LH	0.636029412	0.257676547	0.981422655	0.604055247	2.55690113	1.450831118	0.146053986
154850	F998	N	F	28	RH	0.653333333	0.254815459	1.108238247	0.810273087	2.625538409	1.381406484	0.118405104
154850	F998	N	F	96	LF	0.721062619	0.229285224	1.015609821	0.730128478	3.16585838	1.20964247	0.105395897
154850	F998	N	F	96	RF	0.728125	0.222409694	0.936759214	0.732875209	3.301860378	1.208204918	0.088250388
154850	F998	N	F	96	LH	0.698343505	0.220517935	0.828341442	0.624138459	3.236734483	1.342769365	0.10895846
154850	F998	N	F	96	RH	0.609481916	0.232561888	0.986418832	0.6220644	2.624725489	1.682774361	0.144140661
155005	F1158	N	M	0	LF	0.890692641	0.074522668	0.764073193	0.704409707	13.05199766	1.387212856	0.249764255
155005	F1158	N	M	0	RF	0.767881242	0.168148757	0.612390355	0.330103911	4.767757508	1.76407382	0.114824144

155005	F1158	N	M	0	LH	0.851092896	0.055308887	1.054343693	0.942188101	18.2087421	3.015348687	0.449971802
155005	F1158	N	M	0	RH	0.779016393	0.125936896	0.712207298	0.589823763	8.486587114	1.581475837	0.070661011
155005	F1158	N	M	1	LF	0.878861004	0.111912264	0.722066391	0.572108347	9.19746438	1.205522985	0.183571811
155005	F1158	N	M	1	RF	0.746031746	0.16296688	0.847754638	0.613334019	4.587378207	1.554048499	0.14375741
155005	F1158	N	M	1	LH	0.782894737	0.179767412	0.748311986	0.599916975	4.510007952	1.210728917	0.129691672
155005	F1158	N	M	1	RH	0.850146199	0.117728305	0.710040147	0.550221419	8.337863942	1.295908071	0.176654499
155005	F1158	N	M	2	LF	0.846428571	0.238950936	0.571502844	0.48626829	3.563835153	0.633900252	0.115144506
155005	F1158	N	M	2	RF	0.806428571	0.238950936	0.590528855	0.484762553	3.405188973	0.792546432	0.123999352
155005	F1158	N	M	2	LH	0.795045045	0.172972228	0.585062556	0.498203104	4.594350854	1.187792947	0.150527139
155005	F1158	N	M	2	RH	0.694827586	0.21403681	0.648132844	0.441868827	3.247455416	1.425762217	0.085609099
155005	F1158	N	M	4	LF	0.773504274	0.240373939	0.726479348	0.565907266	3.220331769	0.94240107	0.103184704
155005	F1158	N	M	4	RF	0.742068966	0.237216676	0.871867345	0.610156001	3.142259793	1.100390809	0.121147596
155005	F1158	N	M	4	LH	0.666666667	0.229325784	0.910060273	0.5101686	2.987038737	1.493519369	0.180616884
155005	F1158	N	M	4	RH	0.716524217	0.240373939	0.694009354	0.513088671	2.985192948	1.17753989	0.098766676
155005	F1158	N	M	6	LF	0.775210084	0.216437537	0.739297182	0.566400849	3.620891096	1.056994858	0.110229941
155005	F1158	N	M	6	RF	0.84705228	0.221390186	0.75218269	0.616196343	3.8350249	0.68351535	0.066892496
155005	F1158	N	M	6	LH	0.68543956	0.246302054	0.670508864	0.459101214	2.7888495	1.281948119	0.133912785
155005	F1158	N	M	6	RH	0.740856844	0.215207128	0.756278857	0.558264023	3.470200958	1.205521104	0.123599891
155005	F1158	N	M	8	LF	0.785164835	0.209603565	0.701626641	0.558662728	3.745638545	1.102248796	0.170933453
155005	F1158	N	M	8	RF	0.788793103	0.266092723	0.475685317	0.408488353	3.125278284	0.797316562	0.200256793
155005	F1158	N	M	8	LH	0.805405405	0.189786146	0.784716711	0.613447927	4.316005231	1.03826083	0.133593959
155005	F1158	N	M	8	RH	0.793859649	0.233401831	0.558788883	0.438447275	3.943586434	0.790319367	0.073614807
155005	F1158	N	M	24	LF	0.797914996	0.197665278	0.943572186	0.768621536	4.248591788	1.023797159	0.099588658
155005	F1158	N	M	24	RF	0.759504863	0.205762948	1.069457744	0.772813122	3.807779744	1.170053896	0.090630399
155005	F1158	N	M	24	LH	0.677843803	0.200394834	1.157270597	0.704447799	3.440606527	1.608824108	0.138378183
155005	F1158	N	M	24	RH	0.731191223	0.195870908	1.019002063	0.712374402	3.956078314	1.38994946	0.086096123
155005	F1158	N	M	26	LF	0.72983871	0.231528531	1.177968632	0.89865813	3.156725045	1.174954107	0.109128216
155005	F1158	N	M	26	RF	0.704926108	0.214682986	1.120326092	0.842888223	3.302657736	1.394073514	0.125682237
155005	F1158	N	M	26	LH	0.644097222	0.200995195	1.163665438	0.779558399	3.228589541	1.762210791	0.122963742
155005	F1158	N	M	26	RH	0.697580645	0.216299499	1.172135385	0.926384718	3.23079324	1.394073514	0.152539931
155005	F1158	N	M	28	LF	0.724537037	0.268462488	1.205010572	0.846491992	2.716572955	1.026920592	0.132062528
155005	F1158	N	M	28	RF	0.670914543	0.266104598	1.29516692	0.882293502	2.57158237	1.247831182	0.146880868
155005	F1158	N	M	28	LH	0.657692308	0.301999025	1.193749366	0.817180947	2.202256481	1.177048246	0.132596098

155005	F1158	N	M	28	RH	0.66091954	0.280384101	1.212672067	0.735575375	2.424879429	1.249543539	0.160751075
155005	F1158	N	M	96	LF	0.694444444	0.188071524	1.220143573	0.838471185	3.694128636	1.635910633	0.122322365
155005	F1158	N	M	96	RF	0.754261364	0.21666042	1.002441321	0.819497718	3.480349456	1.137967712	0.109606544
155005	F1158	N	M	96	LH	0.745238095	0.218022354	0.968035681	0.769447845	3.408652147	1.212290516	0.0514865
155005	F1158	N	M	96	RH	0.690079365	0.198341105	1.144471854	0.788153208	3.481662204	1.562900577	0.116276062
155029	F1571	N	F	0	LF	0.914473684	0.036009478	2.22366927	1.801185742	25.39536088	2.375105694	0.163982451
155029	F1571	N	F	0	RF	0.921568627	0.107322365	1.057301163	0.9667308	8.586920586	0.730801752	0.015617375
155029	F1571	N	F	0	LH	0.854545455	0.099517102	1.222326134	0.9667308	8.586920586	1.461603504	0.148365071
155029	F1571	N	F	0	RH	0.909090909	0.497585512	0.0463634	0.054737424	1.82700438	0.182700438	0
155029	F1571	N	F	1	LF	0.831332533	0.235220772	0.47014809	0.412617986	4.706066387	0.74939261	0.197097095
155029	F1571	N	F	1	RF	0.814542484	0.257738187	0.435533958	0.332182674	3.563818806	0.827963604	0.151832199
155029	F1571	N	F	1	LH	0.862424242	0.131771791	0.716544103	0.60062418	7.994040362	1.086428558	0.01309871
155029	F1571	N	F	1	RH	0.720257717	0.106399538	0.718096952	0.394878537	7.486861299	2.398819254	0.131603547
155029	F1571	N	F	2	LF	0.85952381	0.168993144	0.674016603	0.582431714	5.217146374	0.885018511	0.109680541
155029	F1571	N	F	2	RF	0.846666667	0.18273467	0.564675917	0.52327888	5.008991622	0.882398536	0.125125928
155029	F1571	N	F	2	LH	0.868107303	0.132671458	0.713833678	0.61804278	10.30211728	1.178278032	0.022001274
155029	F1571	N	F	2	RH	0.782679739	0.162132097	0.684751748	0.52507977	5.080996514	1.317047866	0.133908001
155029	F1571	N	F	4	LF	0.787683032	0.160392511	0.759984217	0.631622628	9.476828531	1.17752069	0.262451078
155029	F1571	N	F	4	RF	0.828636364	0.144698096	0.469342265	0.287794648	7.999512906	1.107293874	0.171220097
155029	F1571	N	F	4	LH	0.790247678	0.153153452	0.734474996	0.532063327	6.035276764	1.512319587	0.152290308
155029	F1571	N	F	4	RH	0.799026059	0.150863756	0.682371513	0.466402922	9.483829323	1.446759966	0.168394845
155029	F1571	N	F	8	LF	0.771202237	0.095516854	0.749679221	0.578212574	9.069175179	2.628765684	0.237685915
155029	F1571	N	F	8	RF	0.938359788	0.059289305	0.846844284	0.716033483	16.01953242	1.033695177	0.130789508
155029	F1571	N	F	8	LH	0.87780112	0.103005843	0.789438987	0.663607131	9.886188697	1.306347281	0.164833715
155029	F1571	N	F	8	RH	0.911441353	0.063184732	0.911418795	0.744323879	14.46851825	1.38415298	0.096475541
155029	F1571	N	F	24	LF	0.84375	0.15332386	0.845242574	0.747803282	5.502847761	1.019533433	0.116029146
155029	F1571	N	F	24	RF	0.883484163	0.142908862	0.708307604	0.665871638	6.182262469	0.815297649	0.120022039
155029	F1571	N	F	24	LH	0.76509434	0.143050348	0.682347487	0.561045821	5.371078536	1.628127068	0.143579644
155029	F1571	N	F	24	RH	0.730989957	0.161796404	0.781321241	0.621423094	4.550021683	1.696479911	0.097104027
155029	F1571	N	F	26	LF	0.869249395	0.096630184	1.007676859	0.750069008	9.786593952	1.517857073	0.192146119
155029	F1571	N	F	26	RF	0.767385445	0.172223828	1.033121186	0.774421832	4.725117426	1.307871287	0.099128983
155029	F1571	N	F	26	LH	0.635781671	0.197923943	0.904695455	0.524580226	3.69210191	1.854507663	0.146375273
155029	F1571	N	F	26	RH	0.826006356	0.099842698	0.963808838	0.750622098	9.03458454	1.644521876	0.138560507

155029	F1571	N	F	28	LF	0.693228454	0.190879398	0.883810552	0.707094167	3.657649132	1.657139291	0.174906186
155029	F1571	N	F	28	RF	0.812698413	0.161148628	0.837811367	0.705013777	5.524526145	1.242763405	0.116985829
155029	F1571	N	F	28	LH	0.773023023	0.165097973	0.809371788	0.613535001	4.972146381	1.3101262	0.093205842
155029	F1571	N	F	28	RH	0.661533627	0.182216421	0.845509409	0.603444814	3.658013386	1.863234472	0.107332102
155029	F1571	N	F	96	LF	0.818148148	0.118178441	0.94443469	0.72947658	7.166675616	1.552909894	0.159474227
155029	F1571	N	F	96	RF	0.790849673	0.155053722	0.960812381	0.771422627	5.133068459	1.349803518	0.073890659
155029	F1571	N	F	96	LH	0.695652174	0.161068333	0.919337718	0.671555046	4.316827862	1.892268319	0.108539574
155029	F1571	N	F	96	RH	0.793779904	0.132370599	0.85546742	0.644356913	6.011076594	1.549094802	0.119372854
155362	F1158	N	M	0	LF	0.566666667	0.206720368	1.268554906	0.973961587	2.74122319	2.096229498	0.2104238
155362	F1158	N	M	0	RF	0.907692308	0.095409401	0.901816281	0.781574109	9.513656952	0.967490537	0.204411691
155362	F1158	N	M	0	LH	0.794871795	0.159015668	0.531818499	0.623765698	4.99870111	1.289987383	0.03607265
155362	F1158	N	M	0	RH	0.636363636	0.281891411	0.567175461	0.348702297	2.257477921	1.289987383	0.234472233
155362	F1158	N	M	1	LF	0.799019608	0.159883624	0.820005493	0.477837922	5.00609566	1.252428504	0.172354061
155362	F1158	N	M	1	RF	0.613636364	0.284048593	0.645662146	0.488326926	2.766773194	1.430054592	0.166490498
155362	F1158	N	M	1	LH	0.707222222	0.189387956	0.887642982	0.72638164	3.842781729	1.608283739	0.18448807
155362	F1158	N	M	1	RH	0.828947368	0.147155198	1.025814145	0.759554544	5.63351603	1.162107812	0.14165911
155362	F1158	N	M	2	LF	0.81037037	0.211724186	0.72766155	0.540765899	3.823662355	0.900368367	0.113985104
155362	F1158	N	M	2	RF	0.78014553	0.180930654	0.750578197	0.558750202	4.369259786	1.384155115	0.172972368
155362	F1158	N	M	2	LH	0.75902439	0.178192187	0.588339844	0.506427827	4.773314484	1.264099987	0.055412527
155362	F1158	N	M	2	RH	0.74625	0.196598137	0.747534298	0.58846934	3.930276457	1.268580329	0.131858774
155362	F1158	N	M	4	LF	0.674166667	0.257938959	1.323237528	0.901154649	2.612114868	1.268824955	0.116188277
155362	F1158	N	M	4	RF	0.693452381	0.244119362	1.240375883	0.900262283	2.848721922	1.265050046	0.154618301
155362	F1158	N	M	4	LH	0.661375661	0.266917585	1.105086329	0.698774399	2.528212638	1.266937501	0.105631537
155362	F1158	N	M	4	RH	0.591269841	0.326089844	0.967685425	0.62846419	1.822166385	1.268824955	0.117407258
155362	F1158	N	M	6	LF	0.801075269	0.557307152	0.513390416	0.437499228	7.3763175	0.645763723	0.111810796
155362	F1158	N	M	6	RF	0.826190476	0.192876188	0.638766951	0.614724366	4.343322517	0.88095395	0.130830992
155362	F1158	N	M	6	LH	0.790865385	0.21679994	0.534672079	0.51700033	3.68935742	0.963724759	0.049201612
155362	F1158	N	M	6	RH	0.786111111	0.191490348	0.656971348	0.488912777	7.767207697	1.124345553	0.082438934
155362	F1158	N	M	8	LF	0.821808511	0.184909306	1.125669366	0.949248259	4.838439877	0.924206666	0.16831975
155362	F1158	N	M	8	RF	0.713352685	0.185915105	1.040434822	0.740368004	4.528060704	1.538900515	0.211968072
155362	F1158	N	M	8	LH	0.69537037	0.282561526	0.706671128	0.468071311	2.536153601	1.07723036	0.157033767
155362	F1158	N	M	8	RH	0.600790514	0.288994482	0.643183724	0.36745002	2.077082517	1.38587682	0.101447924
155362	F1158	N	M	24	LF	0.83968254	0.16758115	0.99111246	0.844367218	5.092043209	0.989543815	0.131578402

155362	F1158	N	M	24	RF	0.791052919	0.154732251	1.02207286	0.729514275	5.167010927	1.368123276	0.124200263
155362	F1158	N	M	24	LH	0.783613445	0.175547908	0.915024085	0.689835146	4.479208421	1.296896426	0.375531408
155362	F1158	N	M	24	RH	0.754728132	0.161857825	0.989155827	0.762468371	4.792172334	1.518058713	0.248476533
155362	F1158	N	M	26	LF	0.750957854	0.248355219	1.021593166	0.805019413	3.023325821	1.007172638	0.142672963
155362	F1158	N	M	26	RF	0.682051282	0.249422261	1.057215466	0.729880646	2.735390028	1.296916338	0.153105511
155362	F1158	N	M	26	LH	0.764615385	0.272523789	0.960467406	0.747792352	2.807675295	0.863807377	0.107585099
155362	F1158	N	M	26	RH	0.622019635	0.26319972	0.978845308	0.61153254	2.375771606	1.513772136	0.138250262
155362	F1158	N	M	28	LF	0.760121457	0.218354131	1.050262728	0.80577307	3.647733914	1.11508781	0.13016375
155362	F1158	N	M	28	RF	0.763888889	0.21390285	1.116810539	0.910127866	3.645088879	1.11508781	0.13941227
155362	F1158	N	M	28	LH	0.606060606	0.203953085	1.149166081	0.775325581	2.971804137	1.931672689	0.112249748
155362	F1158	N	M	28	RH	0.657308378	0.200927079	0.973853628	0.622225037	3.270307068	1.706803602	0.118287289
160013	F1546	N	M	0	LF	0.875	0.098221729	0.230620506	0.168028752	8.908415792	1.272630827	0.107701702
160013	F1546	N	M	0	RF	0.418604651	0.12791667	-0.16604261	-0.23432328	3.272479271	4.545110098	0.086063661
160013	F1546	N	M	0	LH	0.714285714	0.130962305	0.208265754	0.059180403	5.454132118	2.181652847	0.101711608
160013	F1546	N	M	0	RH	0.852459016	0.090170767	0.422041184	0.305949069	9.453829004	1.636239635	0
160013	F1546	N	M	1	LF	0.755952381	0.250732861	0.671593873	0.534032456	3.150410949	1.024002009	0.181078117
160013	F1546	N	M	1	RF	0.704874835	0.234532804	0.783288613	0.55596079	3.1510032	1.259216778	0.081861517
160013	F1546	N	M	1	LH	0.643181818	0.303277408	0.565905695	0.349325569	2.125224438	1.180811855	0.076041365
160013	F1546	N	M	1	RH	0.600724638	0.244122714	0.679464467	0.40629213	2.600984237	1.572244219	0.108073284
160013	F1546	N	M	2	LF	0.690981432	0.201902006	0.791785508	0.592402801	3.529601268	1.576797981	0.151011364
160013	F1546	N	M	2	RF	0.663034002	0.197825355	0.685159878	0.476840848	3.359804728	1.714270492	0.128995679
160013	F1546	N	M	2	LH	0.740447344	0.205065571	0.57007716	0.459666562	3.650911763	1.269528931	0.106402789
160013	F1546	N	M	2	RH	0.637267905	0.243626068	0.614038561	0.449493605	2.615875124	1.491899711	0.121383031
160013	F1546	N	M	4	LF	0.714912281	0.167109055	0.713954038	0.501880772	4.273261827	1.71684149	0.10110563
160013	F1546	N	M	4	RF	0.806976744	0.188212985	0.787647243	0.650151574	4.40659175	1.046474008	0.107911411
160013	F1546	N	M	4	LH	0.633458647	0.167109055	0.932692474	0.557666879	3.809789507	2.18031381	0.08929748
160013	F1546	N	M	4	RH	0.69017094	0.178384391	0.81296659	0.563499323	3.908195743	1.71684149	0.090117613
160013	F1546	N	M	6	LF	0.737209302	0.154451607	0.723879949	0.53446824	4.789287619	1.690899702	0.089347843
160013	F1546	N	M	6	RF	0.791666667	0.176614534	0.724670816	0.57892195	4.704059571	1.178032461	0.091800087
160013	F1546	N	M	6	LH	0.80621118	0.170678191	0.687254481	0.551433217	4.852322413	1.105918609	0.102162665
160013	F1546	N	M	6	RH	0.747447447	0.16734506	0.742666922	0.530035826	4.492761935	1.548689566	0.107185778
160013	F1546	N	M	8	LF	0.708333333	0.227635682	0.796143934	0.564499214	3.150227191	1.274950137	0.127798331
160013	F1546	N	M	8	RF	0.715795328	0.222475051	0.852248802	0.64092536	3.225102598	1.274950137	0.079871028

160013	F1546	N	M	8	LH	0.720272904	0.211197271	0.903612355	0.65250838	3.525348785	1.350073731	0.130470067
160013	F1546	N	M	8	RH	0.673076923	0.213700273	0.868175382	0.58052962	3.300391649	1.575113595	0.125126596
160013	F1546	N	M	24	LF	0.730555556	0.210704008	0.679012494	0.511665462	3.483863565	1.308130277	0.108596508
160013	F1546	N	M	24	RF	0.657509158	0.21165643	0.741183256	0.498783845	3.27187779	1.595261816	0.088468491
160013	F1546	N	M	24	LH	0.69924812	0.213964852	0.729696153	0.509441197	3.340815161	1.453248145	0.07425064
160013	F1546	N	M	24	RH	0.617554859	0.223126482	0.67364195	0.435065031	2.758274225	1.73831022	0.127600478
160013	F1546	N	M	26	LF	0.655913978	0.211658357	0.740749638	0.506698153	3.101379608	1.625318666	0.104408285
160013	F1546	N	M	26	RF	0.76686217	0.211792827	0.801785387	0.617667886	3.619277197	1.110424042	0.056097529
160013	F1546	N	M	26	LH	0.670982143	0.202361618	0.861978226	0.55490981	3.32226323	1.625318666	0.117553817
160013	F1546	N	M	26	RH	0.644686907	0.208611394	0.766108894	0.487154182	3.099878125	1.699947529	0.11939531
160013	F1546	N	M	28	LF	0.740625	0.191534643	0.650925326	0.526792072	3.892850556	1.396239496	0.070293662
160013	F1546	N	M	28	RF	0.756379585	0.192771741	0.627897235	0.484712277	3.967168816	1.247954253	0.072872822
160013	F1546	N	M	28	LH	0.710080645	0.194974458	0.607448188	0.429586859	3.747375524	1.468450098	0.052851346
160013	F1546	N	M	28	RH	0.702702703	0.184008417	0.686168135	0.512228246	3.819586126	1.614978959	0.093243894
160013	F1546	N	M	96	LF	0.768800813	0.160006982	0.837959896	0.653050568	4.804942282	1.484070519	0.11935137
160013	F1546	N	M	96	RF	0.77173913	0.153827143	0.865145116	0.641700657	5.017007009	1.483797255	0.093608699
160013	F1546	N	M	96	LH	0.737946682	0.168571358	0.833116669	0.627747921	4.380922134	1.55454015	0.060841778
160013	F1546	N	M	96	RH	0.728778468	0.161155078	0.802094982	0.57976569	4.522298619	1.695971288	0.074879205
160021	F1768	N	M	0	LF	0.450980392	0.235776308	0.551438856	0.258472363	2.89872392	2.398058238	0.331759543
160021	F1768	N	M	0	RF	0.746859903	0.123928298	0.868109508	0.672709052	6.023388131	2.048117766	0.307684789
160021	F1768	N	M	0	LH	0.747835498	0.152079872	0.537988643	0.461911039	4.953726687	1.678337265	0.015689292
160021	F1768	N	M	0	RH	0.798259188	0.111024632	1.136970198	1.04020195	7.184254699	1.84586749	0.144237056
160021	F1768	N	M	1	LF	0.726851852	0.230378032	1.088353153	0.768087237	3.158780512	1.184014139	0.140967346
160021	F1768	N	M	1	RF	0.66875	0.257499595	0.886521947	0.689007773	2.766122667	1.343131083	0.111840794
160021	F1768	N	M	1	LH	0.654808959	0.233782922	0.842451449	0.55826768	3.004496052	1.419971282	0.146711734
160021	F1768	N	M	1	RH	0.523284314	0.225213356	1.101735947	0.620940469	2.450305022	2.132071135	0.214750305
160021	F1768	N	M	2	LF	0.737616099	0.173089906	0.69457635	0.537520405	4.264392681	1.527634815	0.128952899
160021	F1768	N	M	2	RF	0.846938776	0.107691214	0.839809421	0.647123031	8.119302618	1.447586761	0.157194562
160021	F1768	N	M	2	LH	0.847705915	0.103848656	0.930692298	0.676957796	8.2819194	1.449267212	0.141654911
160021	F1768	N	M	2	RH	0.729166667	0.161648917	0.653689671	0.525069144	4.7413201	1.689411372	0.073301234
160021	F1768	N	M	4	LF	0.671428571	0.219745206	1.043619268	0.677251833	3.061737279	1.492736609	0.154929316
160021	F1768	N	M	4	RF	0.71945259	0.199177635	0.972430897	0.763170751	3.612891321	1.414650904	0.131655224
160021	F1768	N	M	4	LH	0.727272727	0.192918377	0.894996272	0.753247891	3.769062731	1.414650904	0.110710983

160021	F1768	N	M	4	RH	0.700721154	0.221769404	1.02381854	0.738817635	3.142555449	1.410096796	0.131564336
160021	F1768	N	M	6	LF	0.713218391	0.216386387	1.023186241	0.729664589	3.291538328	1.330720781	0.127687746
160021	F1768	N	M	6	RF	0.686961207	0.209780878	0.832777112	0.632422279	3.291538328	1.48836346	0.174148364
160021	F1768	N	M	6	LH	0.727777778	0.195055869	0.894193854	0.64134918	3.761622676	1.41072699	0.157063374
160021	F1768	N	M	6	RH	0.622727273	0.203089441	0.903532235	0.594860942	3.054837337	1.881759234	0.145369266
160021	F1768	N	M	8	LF	0.713286713	0.196856719	0.98918259	0.752779931	3.979433844	1.479667681	0.13069042
160021	F1768	N	M	8	RF	0.740384615	0.185705224	0.938034242	0.675271817	4.525988958	1.56020398	0.119528248
160021	F1768	N	M	8	LH	0.752978056	0.169493706	0.944715999	0.734404753	4.994157646	1.559129044	0.130623019
160021	F1768	N	M	8	RH	0.701178451	0.192250349	0.926440998	0.688007575	3.979433844	1.556979171	0.113643842
160021	F1768	N	M	24	LF	0.708494208	0.187657213	1.165755288	0.8101373	3.778778487	1.556236975	0.120700086
160021	F1768	N	M	24	RF	0.724423963	0.20520099	1.042305372	0.780668164	3.553318921	1.33444074	0.187271388
160021	F1768	N	M	24	LH	0.631338742	0.215515501	0.985373101	0.636841353	2.960641184	1.704406409	0.186892285
160021	F1768	N	M	24	RH	0.672043011	0.211080649	1.064989638	0.722250415	3.185184918	1.555321142	0.163270003
160021	F1768	N	M	26	LF	0.677083333	0.136806275	1.019433725	0.663788734	4.953162652	2.359849539	0.126237333
160021	F1768	N	M	26	RF	0.762310606	0.138523082	0.922138022	0.767209039	5.50631559	1.733918167	0.158031106
160021	F1768	N	M	26	LH	0.736714976	0.128069584	0.928342925	0.725984332	5.829366791	2.043522014	0.163541831
160021	F1768	N	M	26	RH	0.722272727	0.135717365	0.983717659	0.675265825	5.34394953	2.045202933	0.163698123
160021	F1768	N	M	28	LF	0.694252874	0.221939394	1.000120122	0.739934522	3.130406117	1.375862104	0.125553218
160021	F1768	N	M	28	RF	0.706190061	0.193936348	0.888966632	0.7196487	3.6646396	1.526778666	0.136232783
160021	F1768	N	M	28	LH	0.680519481	0.192730627	0.770504893	0.650349088	3.510015028	1.685111246	0.093184618
160021	F1768	N	M	28	RH	0.658730159	0.207652045	1.031566691	0.713696472	3.206327899	1.678622231	0.103270351
160021	F1768	N	M	96	LF	0.706356736	0.214255897	1.095860571	0.76187517	3.318439275	1.367442488	0.059129584
160021	F1768	N	M	96	RF	0.707706767	0.215889549	1.029958534	0.799634724	3.396539014	1.369868583	0.09091958
160021	F1768	N	M	96	LH	0.646286572	0.193245123	1.049656854	0.699719108	3.547886301	1.797223404	0.124032147
160021	F1768	N	M	96	RH	0.633391456	0.206116588	1.128425056	0.691694159	3.101122722	1.804501689	0.139927149
160096	F1034	N	F	0	LF	0.825	0.158488788	0.557311777	0.483416016	5.205415548	1.104179056	0.164260314
160096	F1034	N	F	0	RF	0.891304348	0.137816337	0.351986389	0.310249274	6.467334469	0.788699325	0.076263717
160096	F1034	N	F	0	LH	0.6	0.633955151	0.222924707	0.181859632	0.946439191	0.63095946	0
160096	F1034	N	F	0	RH	0.810810811	0.17133923	0.429871178	0.387185021	4.732195953	1.104179056	0.082130154
160096	F1034	N	F	1	LF	0.686111111	0.274065936	0.378763209	0.222043789	2.742588477	1.198240693	0.212748863
160096	F1034	N	F	1	RF	0.7	0.29401137	0.492950246	0.396729922	2.466115815	1.056906778	0.143300035
160096	F1034	N	F	1	LH	0.741798942	0.231716344	0.531879117	0.429699385	3.244484876	1.128606264	0.126865648
160096	F1034	N	F	1	RH	0.65	0.215953014	0.629323057	0.394295405	3.033516531	1.624307494	0.203931904

160096	F1034	N	F	2	LF	0.672580645	0.289285909	0.77829209	0.506178867	2.421333595	1.211315105	0.129373467
160096	F1034	N	F	2	RF	0.581989247	0.259874561	0.858396017	0.546935876	2.278368307	1.63761774	0.127087492
160096	F1034	N	F	2	LH	0.666666667	0.292779759	0.747284495	0.544820757	2.278368307	1.137239232	0.105500537
160096	F1034	N	F	2	RH	0.603896104	0.327035412	0.69095751	0.385198218	1.850769057	1.209370183	0.117276378
160096	F1034	N	F	4	LF	0.680194805	0.278603145	0.820506033	0.606824158	2.487204461	1.170595971	0.15248013
160096	F1034	N	F	4	RF	0.64258312	0.250498519	0.797329514	0.528663817	2.712462778	1.46262101	0.145680942
160096	F1034	N	F	4	LH	0.633040936	0.217634268	0.909133004	0.569758978	3.081237799	1.677896066	0.212048167
160096	F1034	N	F	4	RH	0.62084399	0.250498519	0.870857255	0.558607006	2.640704426	1.534379362	0.155231639
160096	F1034	N	F	6	LF	0.719375	0.248395165	0.833683342	0.595840162	2.949066873	1.150896863	0.124382779
160096	F1034	N	F	6	RF	0.64031339	0.262883195	0.820279827	0.541108669	2.445443157	1.36211747	0.065595304
160096	F1034	N	F	6	LH	0.597297297	0.233868339	0.875497844	0.60148278	2.663469427	1.799871426	0.147969337
160096	F1034	N	F	6	RH	0.615942029	0.248702769	0.91100743	0.556367771	2.666872258	1.581845156	0.128460011
160096	F1034	N	F	8	LF	0.778677463	0.178187181	0.688400447	0.531696896	4.376733723	1.237748711	0.113557575
160096	F1034	N	F	8	RF	0.764502165	0.201845345	0.702275517	0.536626731	3.790093111	1.166528775	0.081233156
160096	F1034	N	F	8	LH	0.688744589	0.201937004	0.73265303	0.475353369	3.430617206	1.528255497	0.102595758
160096	F1034	N	F	8	RH	0.665750916	0.16952641	0.740511911	0.479358202	3.9347838	1.969080012	0.125349674
160096	F1034	N	F	24	LF	0.749843652	0.178335878	0.799737886	0.560108493	4.20725001	1.402606565	0.10584821
160096	F1034	N	F	24	RF	0.68670151	0.17181655	0.88894885	0.626556149	3.997143868	1.823388535	0.114993704
160096	F1034	N	F	24	LH	0.666666667	0.169754778	0.894376855	0.616990943	3.927298382	1.963649191	0.110450304
160096	F1034	N	F	24	RH	0.641768293	0.176059999	0.837886431	0.523066635	3.646207384	2.034064362	0.108139474
160096	F1034	N	F	26	LF	0.732142857	0.196382124	0.920473273	0.668295703	3.728872033	1.363441798	0.064856948
160096	F1034	N	F	26	RF	0.692234848	0.214515748	0.98553983	0.660521494	3.227317761	1.434612362	0.091873714
160096	F1034	N	F	26	LH	0.616366366	0.19100032	1.080558768	0.667828143	3.227317761	2.008457307	0.151859562
160096	F1034	N	F	26	RH	0.615384615	0.178742283	1.202521514	0.72660294	3.444189777	2.150798435	0.094252834
160096	F1034	N	F	28	LF	0.748005319	0.18617238	0.928071955	0.628361453	4.249968326	1.341938733	0.078703401
160096	F1034	N	F	28	RF	0.655555556	0.179924575	1.114389623	0.750493363	3.752132371	1.907235173	0.095204983
160096	F1034	N	F	28	LH	0.660080645	0.202826474	0.961043981	0.699075914	3.325598786	1.69716995	0.079041067
160096	F1034	N	F	28	RH	0.612601626	0.204527604	0.982602091	0.570813709	3.115533562	1.908515801	0.125585471
160096	F1034	N	F	96	LF	0.695601852	0.250275951	1.095414486	0.769602578	2.798633059	1.228868599	0.107645774
160096	F1034	N	F	96	RF	0.655913978	0.253953336	1.057227919	0.708550139	2.593625971	1.365344336	0.131917465
160096	F1034	N	F	96	LH	0.579831933	0.238703096	1.108702034	0.651437774	2.457737198	1.774771548	0.096854202
160096	F1034	N	F	96	RH	0.635746606	0.248811557	1.092233316	0.672138594	2.594212936	1.502407038	0.07688984
160121	F1546	N	F	0	LF	0.87804878	0.166941214	0.229698717	0.209724918	5.25962859	0.730503971	0.099869004

160121	F1546	N	F	0	RF	0.720930233	0.159176506	0.44775099	0.186336841	4.529124619	1.75320953	0.138580294
160121	F1546	N	F	0	LH	0.777777778	0.190127493	0.490146358	0.321730944	4.090822236	1.168806353	0.039947602
160121	F1546	N	F	0	RH	0.512195122	0.166941214	0.244679068	0.170018752	3.068116677	2.922015883	0.03987732
160121	F1546	N	F	1	LF	0.723011364	0.18714623	0.737248674	0.520412765	4.013811321	1.531471779	0.13762348
160121	F1546	N	F	1	RF	0.746700189	0.173849503	0.81516257	0.567101807	4.374226751	1.44658588	0.178772986
160121	F1546	N	F	1	LH	0.780357143	0.168466607	0.894711298	0.682510888	4.649756283	1.306359035	0.139210276
160121	F1546	N	F	1	RH	0.737373737	0.182087008	0.763383329	0.552621012	4.238924065	1.451510039	0.142912636
160121	F1546	N	F	2	LF	0.778677463	0.267099507	0.741999946	0.605848798	3.367442645	0.878670006	0.0937663
160121	F1546	N	F	2	RF	0.607142857	0.182810761	0.730223197	0.578529438	3.733695732	2.415920767	0.143860317
160121	F1546	N	F	2	LH	0.683181818	0.21406778	0.620994044	0.49329332	3.732928913	1.31884851	0.123705671
160121	F1546	N	F	2	RH	0.50297619	0.269173524	0.726343057	0.403912203	2.049667681	1.830958703	0.093532223
160121	F1546	N	F	4	LF	0.785714286	0.242396634	0.697985198	0.590927305	3.241440578	0.884029248	0.117253883
160121	F1546	N	F	4	RF	0.827586207	0.234038129	0.621955382	0.502078401	3.536116994	0.73669104	0.086665911
160121	F1546	N	F	4	LH	0.756756757	0.183435291	0.776187904	0.586269416	4.125469826	1.326043873	0.142743858
160121	F1546	N	F	4	RH	0.612903226	0.218938895	0.69351195	0.494505508	2.799425953	1.768058497	0.158037842
160121	F1546	N	F	6	LF	0.752619967	0.166319088	0.908900143	0.711073675	4.654785014	1.501390075	0.144450009
160121	F1546	N	F	6	RF	0.74005102	0.159129436	0.954524785	0.70878641	4.72333992	1.644287735	0.138821555
160121	F1546	N	F	6	LH	0.744135297	0.16440126	0.960901154	0.712696249	4.578512978	1.573803546	0.13927448
160121	F1546	N	F	6	RH	0.735459184	0.159129436	0.966250781	0.696167129	4.727198486	1.64042917	0.210469803
160121	F1546	N	F	8	LF	0.846128822	0.181480266	0.553098155	0.497285815	4.981555056	0.935125515	0.133001885
160121	F1546	N	F	8	RF	0.862533693	0.134736315	0.495580293	0.462534601	6.402123906	1.020055785	0.098446793
160121	F1546	N	F	8	LH	0.685185185	0.157972012	0.448603597	0.367897653	4.605180276	1.908872286	0.110993334
160121	F1546	N	F	8	RH	0.750215703	0.145575151	0.619673728	0.487878609	5.540305791	1.617427776	0.109962447
160121	F1546	N	F	24	LF	0.731191223	0.206857983	0.962686679	0.729749493	3.728443758	1.312679483	0.178780237
160121	F1546	N	F	24	RF	0.734058515	0.200751381	0.999298483	0.747776689	3.797751917	1.313097037	0.12301838
160121	F1546	N	F	24	LH	0.695869837	0.183302085	0.995894973	0.769337952	3.935950681	1.658385171	0.123553228
160121	F1546	N	F	24	RH	0.627327128	0.189932745	1.068917919	0.653176505	3.520519281	1.935200253	0.136626575
160121	F1546	N	F	26	LF	0.696969697	0.227789121	0.879679801	0.629734149	3.073304369	1.326981054	0.118189794
160121	F1546	N	F	26	RF	0.675	0.23012329	0.830973653	0.589144865	3.003694825	1.39677782	0.152262323
160121	F1546	N	F	26	LH	0.642045455	0.257637686	0.793217814	0.534592406	2.514462186	1.46704264	0.11368016
160121	F1546	N	F	26	RH	0.625874126	0.246157469	0.829660899	0.501587194	2.584446174	1.536652185	0.104592917
160121	F1546	N	F	28	LF	0.731547017	0.163493703	0.865798565	0.631406658	4.472415204	1.652206004	0.124015
160121	F1546	N	F	28	RF	0.725998962	0.165951326	0.968931949	0.671323947	4.404971932	1.651425365	0.066193837

160121	F1546	N	F	28	LH	0.734901278	0.175127996	0.945417507	0.666055516	4.197567963	1.513806585	0.106394721
160121	F1546	N	F	28	RH	0.739750908	0.165951326	0.879868381	0.637095723	4.473195843	1.583201454	0.095188547
160121	F1546	N	F	96	LF	0.751710655	0.229635626	0.942025612	0.700111579	3.269768791	1.087196923	0.112735309
160121	F1546	N	F	96	RF	0.732941176	0.254516405	1.028401454	0.733241029	2.925200561	1.087196923	0.123772638
160121	F1546	N	F	96	LH	0.668734491	0.21278875	1.18607484	0.745126156	3.20459367	1.566788552	0.080958551
160121	F1546	N	F	96	RH	0.591594828	0.241240356	1.141220665	0.664307766	2.451450377	1.700643662	0.142233501
160153	F1768	N	M	0	LF	0.790640394	0.141057451	0.840312323	0.928453451	7.13552836	1.573553918	0.094490078
160153	F1768	N	M	0	RF	0.583333333	0.31439551	0.490496351	0.148553101	2.180652708	1.389225725	0.183961658
160153	F1768	N	M	0	LH	0.554347826	0.292835996	0.508743311	0.162289157	2.068195782	1.573553918	0.203050608
160153	F1768	N	M	0	RH	0.709330144	0.268941346	0.352132196	0.258286087	2.679523078	1.119497712	0.004578688
160153	F1768	N	M	1	LF	0.775143678	0.248649564	0.492456802	0.396029039	3.128246136	0.913518961	0.137072156
160153	F1768	N	M	1	RF	0.746376812	0.250583274	0.470314776	0.339068558	2.979060955	1.059022567	0.107922486
160153	F1768	N	M	1	LH	0.787179487	0.234560355	0.452935537	0.338357119	3.359387057	0.913518961	0.099721328
160153	F1768	N	M	1	RH	0.676339286	0.219507265	0.453868558	0.314050735	3.150335586	1.443030245	0.085993254
160153	F1768	N	M	2	LF	0.755698006	0.248887192	0.604786493	0.491554915	3.033205792	0.993656724	0.126543307
160153	F1768	N	M	2	RF	0.928212851	0.095042002	0.585038708	0.504828073	10.14929763	0.759378823	0.120390213
160153	F1768	N	M	2	LH	0.942361111	0.077664449	0.647181725	0.594747997	12.13713267	0.750759819	0.08638178
160153	F1768	N	M	2	RH	0.884060833	0.094116235	0.679305537	0.572773292	9.771762965	1.210696616	0.095444999
160153	F1768	N	M	4	LF	0.760714286	0.20944954	0.576540145	0.489967125	3.692358477	1.153178119	0.156331516
160153	F1768	N	M	4	RF	0.820855615	0.194366839	0.548754576	0.436959766	4.22623549	0.921917211	0.094527229
160153	F1768	N	M	4	LH	0.768067227	0.188674138	0.593631069	0.405557945	4.07101941	1.229222948	0.063851615
160153	F1768	N	M	4	RH	0.772043011	0.213426692	0.51858994	0.41996805	3.614750437	1.070880447	0.108501581
160153	F1768	N	M	6	LF	0.857142857	0.232627966	0.31676712	0.272308578	3.684607962	0.614101327	0.061130495
160153	F1768	N	M	6	RF	0.833333333	0.271399294	0.238964668	0.224943534	3.070506635	0.614101327	0.044458542
160153	F1768	N	M	6	LH	0.857142857	0.116313983	0.955858668	0.72245132	7.369215923	1.228202654	0.12226099
160153	F1768	N	M	6	RH	0.777777778	0.241243817	0.288980531	0.222292715	3.224031967	0.92115199	0.077802448
160153	F1768	N	M	8	LF	0.693181818	0.290069884	1.024888532	0.665537091	2.41003196	1.049780311	0.127551582
160153	F1768	N	M	8	RF	0.645833333	0.277261035	1.008033084	0.739262148	2.336101062	1.271573003	0.152513089
160153	F1768	N	M	8	LH	0.658333333	0.305440502	0.854276861	0.636446873	2.183323352	1.128627124	0.138831467
160153	F1768	N	M	8	RH	0.561904762	0.291934678	1.04741886	0.594511915	1.959072702	1.503197527	0.12053225
160153	F1768	N	M	24	LF	0.764423077	0.225965542	0.945717068	0.785817159	3.694188704	1.048464013	0.136767692
160153	F1768	N	M	24	RF	0.759461733	0.197361939	0.963755045	0.725876428	4.067002493	1.194969299	0.166789631
160153	F1768	N	M	24	LH	0.723577236	0.206079298	0.93296949	0.687500625	3.767441346	1.34802516	0.141248844

160153	F1768	N	M	24	RH	0.75	0.252311836	0.801184249	0.635788431	3.474430775	1.051739301	0.168861945
160153	F1768	N	M	26	LF	0.700909091	0.29631352	0.935566842	0.655632114	2.370423941	1.00834946	0.101436338
160153	F1768	N	M	26	RF	0.680555556	0.272945528	0.888080463	0.683434128	2.505940816	1.160932429	0.074425596
160153	F1768	N	M	26	LH	0.713218391	0.235479341	0.904134006	0.724487717	3.033581426	1.213758035	0.115095014
160153	F1768	N	M	26	RH	0.60989011	0.257409063	1.044976038	0.653657698	2.370423941	1.514657451	0.153051203
160153	F1768	N	M	28	LF	0.732954545	0.245846067	1.030740123	0.732822692	3.080396327	1.099318314	0.070551485
160153	F1768	N	M	28	RF	0.704301075	0.272914294	0.973463522	0.737182186	2.714547927	1.099318314	0.108368608
160153	F1768	N	M	28	LH	0.626451613	0.246755393	0.942262794	0.651568324	2.565371585	1.539754885	0.090639664
160153	F1768	N	M	28	RH	0.65954416	0.257683849	1.039017392	0.676815754	2.565371585	1.317763486	0.112904923
160153	F1768	N	M	96	LF	0.726923077	0.232234521	1.0529912	0.754473195	3.264390793	1.155105002	0.151850671
160153	F1768	N	M	96	RF	0.678523936	0.182077599	1.239467823	0.879449728	3.839117795	1.887641251	0.155863177
160153	F1768	N	M	96	LH	0.704861111	0.204264836	1.037104166	0.740335969	3.474733337	1.446235835	0.104954477
160153	F1768	N	M	96	RH	0.660714286	0.194882136	1.213450587	0.774660953	3.405247045	1.729832004	0.141133212
160777	F1745	N	M	1	LF	0.889203779	0.10093979	0.82759916	0.717886469	8.861165148	1.069097325	0.118639526
160777	F1745	N	M	1	RF	0.718181818	0.261962379	0.338647173	0.273391551	2.762190159	1.06351711	0.085031118
160777	F1745	N	M	1	LH	0.763111888	0.188607213	0.475216772	0.436534625	4.422622801	1.306583275	0.07227639
160777	F1745	N	M	1	RH	0.854876161	0.098821186	0.807184191	0.647239244	8.703771217	1.466767314	0.003036778
160777	F1745	N	M	2	LF	0.766869096	0.1676118	0.855886467	0.591347793	4.573843482	1.392493044	0.187962735
160777	F1745	N	M	2	RF	0.763655462	0.210272139	0.777257063	0.663124322	3.642745472	1.165356074	0.10106659
160777	F1745	N	M	2	LH	0.747916667	0.175066955	0.626207516	0.608570916	4.501691705	1.553412482	0.097166209
160777	F1745	N	M	2	RH	0.756390977	0.177027113	0.829687923	0.618180483	4.263873095	1.391306195	0.096615791
160777	F1745	N	M	4	LF	0.857334526	0.203468799	0.547276673	0.479183462	4.526652029	0.681664108	0.075486579
160777	F1745	N	M	4	RF	0.836170213	0.180081154	0.516297312	0.402295673	4.905674588	0.907924648	0.124697236
160777	F1745	N	M	4	LH	0.706782713	0.164348504	0.725501685	0.421836587	4.526652029	1.742350776	0.172981764
160777	F1745	N	M	4	RH	0.779710145	0.181624175	0.540558212	0.425651161	4.528093274	1.210566197	0.098599813
160777	F1745	N	M	6	LF	0.727272727	0.182430863	0.518013298	0.340970777	3.986566276	1.494962354	0.209828174
160777	F1745	N	M	6	RF	0.846153846	0.154364577	0.782569551	0.631755553	5.48152863	0.996641569	0.190156778
160777	F1745	N	M	6	LH	0.787234043	0.128089755	1.001991498	0.839312685	6.145956342	1.661069282	0.12458548
160777	F1745	N	M	6	RH	0.631578947	0.158426802	0.688498687	0.360642167	3.986566276	2.325496994	0.131142608
160777	F1745	N	M	8	LF	0.801238739	0.16193336	0.772331488	0.626346068	5.003646671	1.249576986	0.179281324
160777	F1745	N	M	8	RF	0.808552632	0.173951521	0.511562873	0.445640311	4.646958502	1.102096865	0.090058412
160777	F1745	N	M	8	LH	0.806398687	0.137890949	0.54256509	0.464087221	5.909882303	1.399726471	0.105901643
160777	F1745	N	M	8	RH	0.769345238	0.151584406	0.746082728	0.64823156	5.089398865	1.555214681	0.129416561

160777	F1745	N	M	24	LF	0.782732448	0.172763884	0.762501815	0.582682115	4.841579469	1.270568955	0.103398666
160777	F1745	N	M	24	RF	0.727732794	0.173714316	0.896121582	0.615944347	4.186808745	1.574124953	0.128969103
160777	F1745	N	M	24	LH	0.726633581	0.193866518	0.858047884	0.620453594	3.822564662	1.422346954	0.161880534
160777	F1745	N	M	24	RH	0.680555556	0.185711468	0.751345179	0.498495713	3.664271986	1.721559833	0.17416882
160777	F1745	N	M	26	LF	0.761498708	0.15777559	0.892439314	0.677609713	4.827034606	1.512589764	0.116641628
160777	F1745	N	M	26	RF	0.775543478	0.162260248	0.849887682	0.693230114	4.832298202	1.367430747	0.099196197
160777	F1745	N	M	26	LH	0.721583065	0.150239168	0.824852768	0.652840205	4.904877711	1.874434587	0.181988821
160777	F1745	N	M	26	RH	0.720930233	0.161388194	0.892375781	0.616408494	4.468347941	1.728222851	0.223411183
160777	F1745	N	M	28	LF	0.78154825	0.230253774	0.785100555	0.611199987	3.662270623	1.025371413	0.088190143
160777	F1745	N	M	28	RF	0.70846395	0.194254889	0.856484795	0.616433508	3.810821003	1.540470662	0.08472289
160777	F1745	N	M	28	LH	0.73855964	0.188585265	0.833696578	0.644554004	4.032037547	1.395138337	0.138416233
160777	F1745	N	M	28	RH	0.696457327	0.199452641	0.872384195	0.616863432	3.665488679	1.682584931	0.253691592
160777	F1745	N	M	96	LF	0.775107604	0.190658961	0.965421684	0.746840334	4.100299285	1.203358338	0.104898289
160777	F1745	N	M	96	RF	0.757142857	0.202271818	0.880216924	0.682453487	3.747963184	1.196588848	0.129358871
160777	F1745	N	M	96	LH	0.730803571	0.211866892	0.835224392	0.638590575	3.463724981	1.271456237	0.090562835
160777	F1745	N	M	96	RH	0.701778386	0.18669146	0.98377037	0.712178163	3.819445827	1.627177083	0.084308228
