# Supplementary material for: Does intrauterine crowding affect locomotor development? A comparative study of motor performance, neuromotor maturation and gait variability among piglets that differ in birth weight and vitality
Source: PLoS One. 2018 Apr 24;13(4):e0195961. doi: 10.1371/journal.pone.0195961 (PMC5915318; doi:10.1371/journal.pone.0195961)
Supplement: S6 Table — (PDF) [file pone.0195961.s006.pdf]

S6. ASYMMETRY INDICES

PIGLET	SOW	CATEGORY	GENDER	AGE (h)	LEG	AI STRIDE FREQ (%)	AI STRIDE LENGTH(%)	AI STEP LENGTH (%)	AI STANCE DURATION (%)	AI SWING DURATION (%)	AI DUTY FACTOR (%)
151301	F1816	L	F	1	FRONT	12.27180527	38.47666722	13.19515228	24.40191388	32.9004329	12.20438327
151301	F1816	L	F	1	HIND	17.70334928	24.80283306	22.51695716	16.97761194	19.04761905	0.736877286
151301	F1816	L	F	2	FRONT	11.42857143	39.0198726	9.986309889	24.49392713	21.11111111	13.24068598
151301	F1816	L	F	2	HIND	73.10344828	25.06548688	23.4307074	94.30379747	33.80952381	25.28755916
151301	F1816	L	F	4	FRONT	5.74452003	29.14291442	85.56570331	33.61823362	33.93939394	28.01215805
151301	F1816	L	F	4	HIND	34.21470122	19.66689128	21.52284275	47.17171717	23.61111111	13.42599581
151301	F1816	L	F	6	FRONT	9.781529295	6.602319627	9.685752452	24.04540764	31.57894737	18.05016689
151301	F1816	L	F	6	HIND	11.40722291	28.45422215	40.30492105	17.77777778	9.263157895	6.392830471
151301	F1816	L	F	8	FRONT	3.511404562	7.094011311	4.111704883	9.25186E-15	20.19230769	3.511404562
151301	F1816	L	F	8	HIND	12.5	56.94661199	72.75878718	22.97297297	24.13793103	10.56162546
151301	F1816	L	F	24	FRONT	7.699275362	11.12305626	10.10683775	4.545454545	15.88235294	3.158677072
151301	F1816	L	F	24	HIND	5.857385399	12.87361564	13.0424608	14.43396226	13.04347826	8.596372865
151301	F1816	L	F	26	FRONT	25.83237658	2.164463867	0.710875612	29.16666667	17.76315789	3.395184559
151301	F1816	L	F	26	HIND	18.97363465	12.38829781	20.49664583	43.43275772	31.73913043	25.25248287
151301	F1816	L	F	28	FRONT	4.166666667	2.219052857	11.01995355	18.76832845	23.52941176	14.62397252
151301	F1816	L	F	28	HIND	7.621161672	4.454717206	7.851749919	6.060606061	21.11111111	7.622716872
151301	F1816	L	F	96	FRONT	21.62679426	14.76447904	13.11044951	38.38383838	27.27272727	17.30103806
151301	F1816	L	F	96	HIND	30.26004728	7.539409658	4.987056302	38.38383838	11.11111111	8.571428571
151302	F1816	L	F	0	FRONT	14.04761905	9.026641191	12.58652321	25.33936652	38.81578947	12.69949066
151302	F1816	L	F	0	HIND	24.4191486	14.49362641	61.21779661	44.75655431	67.24137931	21.41618909
151302	F1816	L	F	1	FRONT	23.88438134	19.97061102	7.627736299	31.43225806	13.43873518	8.00885948
151302	F1816	L	F	1	HIND	37.69216348	11.43294565	22.25663249	46.49350649	47.51718869	9.920485103
151302	F1816	L	F	2	FRONT	17.94107911	18.50779275	29.60922138	7.827260459	40.60150376	10.16397222
151302	F1816	L	F	2	HIND	12.28070175	30.89018403	25.44038147	25.2782194	28.18181818	13.01832208
151302	F1816	L	F	4	FRONT	14.29391504	33.39148104	34.65865376	12.13666497	20.95238095	2.168531784
151302	F1816	L	F	4	HIND	20.16042781	43.45796402	26.21320903	20.10810811	22.22222222	5.419841462
151302	F1816	L	F	6	FRONT	10.06493506	10.00878909	10.18585539	14.60628315	33.07692308	11.72583231
151302	F1816	L	F	6	HIND	23.39181287	27.00011821	33.66491824	19.68253968	29.29292929	3.748733536
151302	F1816	L	F	8	FRONT	9.047619048	22.159918	29.63136299	8.104575163	25.26315789	5.720077439

151302	F1816	L	F	8	HIND	10.72261072	8.15235167	23.6359665	15.23809524	10.6442577	4.528680449
151302	F1816	L	F	24	FRONT	6.079854809	3.711708644	26.97607611	7.886904762	58.33333333	13.94857012
151302	F1816	L	F	24	HIND	5.535448158	5.579984516	6.423996459	4.589495156	8.333333333	3.07461939
151302	F1816	L	F	26	FRONT	15.89642151	39.13580635	35.85063669	16.78240741	12.95546559	1.00435304
151302	F1816	L	F	26	HIND	6.060606061	2.878227163	18.71317689	13.04347826	10	7.038512616
151302	F1816	L	F	28	FRONT	4.761904762	19.72772495	21.06533744	6.564102564	17.77777778	3.327461343
151302	F1816	L	F	28	HIND	7.151979566	32.9998005	37.42326064	15.58441558	19.73244147	9.658021795
151302	F1816	L	F	96	FRONT	7.142857143	16.0491804	18.96823094	32.46753247	62.74509804	25.46992481
151302	F1816	L	F	96	HIND	17.85714286	8.769881757	11.36245778	29.32432432	53.28947368	26.07387604
151306	F1349	L	F	4	FRONT	13.67816092	26.1070161	4.820516167	21.54222766	49.41176471	18.12585694
151306	F1349	L	F	4	HIND	11.18834823	42.99617082	32.25722091	16.50055371	37.66233766	8.811118229
151306	F1349	L	F	6	FRONT	5.882352941	19.37653038	25.30412497	0.425531915	26.66666667	6.307884856
151306	F1349	L	F	6	HIND	17.87878788	12.75064122	53.30264114	55.35714286	44.98023715	39.92030531
151309	F943	L	F	1	FRONT	5.714285714	20.11388377	49.02549583	5.128205128	8.695652174	0.586510264
151309	F943	L	F	1	HIND	58.82352941	46.49904847	9.003887185	72.16494845	25.64102564	14.92537313
151309	F943	L	F	2	FRONT	10.70921986	5.007477407	13.94118372	25.951417	29.06906907	16.93303349
151309	F943	L	F	2	HIND	22.80854552	46.86438572	46.07596219	18.81188119	36.19047619	4.141172485
151309	F943	L	F	4	FRONT	24.12949369	18.48987613	16.70346759	3.858998145	85.71428571	23.9462486
151309	F943	L	F	4	HIND	8.095238095	13.16407907	13.9337057	14.8255814	29.62962963	18.17845081
151309	F943	L	F	6	FRONT	24.63235294	6.451633015	5.220158364	7.42081448	76.66666667	31.77774125
151309	F943	L	F	6	HIND	11.31313131	5.586516726	13.05695037	20.06954794	3.448275862	8.844011426
151309	F943	L	F	8	FRONT	39.02362698	32.544633	23.20384401	41.04499274	30.55555556	4.062009108
151309	F943	L	F	8	HIND	23.98351648	18.23844261	6.147980522	26.66666667	17.69230769	4.473884574
151309	F943	L	F	24	FRONT	22.78406296	27.78372486	25.72612409	17.54791804	48.14814815	5.384487307
151309	F943	L	F	24	HIND	51.81776346	46.32067668	44.78501481	39.49548193	96.1352657	13.64261835
151309	F943	L	F	26	FRONT	15.54252199	9.232209142	6.437332259	21.59090909	7.70988E-14	6.103143119
151309	F943	L	F	26	HIND	13.07477009	10.0693742	14.79282818	11.69491525	17.80538302	5.003949299
151309	F943	L	F	28	FRONT	17.19106247	9.578842118	7.702873509	13.15536316	50.28571429	11.75709297
151309	F943	L	F	28	HIND	13.73743527	2.727448912	12.33378879	22.91247485	11.11111111	9.410988977
151310	F943	L	F	0	FRONT	53.46534653	35.84672954	53.38120507	83.5443038	54.54545455	33.86004515
151310	F943	L	F	0	HIND	103.1578947	146.6666676	128.5608189	89.23076923	133.3333333	18.09002945
151310	F943	L	F	1	FRONT	22.72727273	13.52248208	23.90060768	24.14486922	44.33333333	15.21818428
151310	F943	L	F	1	HIND	77.62237762	53.99237374	61.74736142	114.2857143	52.27272727	49.61079398

151310	F943	L	F	2	FRONT	15.09020619	12.69994651	11.22129158	2.040816327	75.45787546	13.05069891
151310	F943	L	F	2	HIND	38.64152261	47.35447462	57.95573466	47.60312152	13.02521008	12.87502265
151310	F943	L	F	4	FRONT	24.40976391	27.62428374	24.42963808	36.57894737	18.18181818	12.86878783
151310	F943	L	F	4	HIND	14.68253968	22.58561192	3.099392656	11.76470588	21.11111111	4.233680227
151310	F943	L	F	6	FRONT	6.987042808	13.68606367	8.567298067	14.3956044	47.61904762	12.70476418
151310	F943	L	F	6	HIND	3.03030303	18.57103639	10.06437213	11.58730159	27.07692308	14.57823814
151310	F943	L	F	8	FRONT	29.743004	107.8631731	124.5883344	26.3732834	50.54945055	3.543069281
151310	F943	L	F	8	HIND	39.21568627	91.86894657	118.3953823	57.80885781	24.46236559	20.83733544
151310	F943	L	F	24	FRONT	19.02197023	3.467908518	9.707076724	21.258572	9.090909091	2.257083116
151310	F943	L	F	24	HIND	21.69934641	10.4099754	29.97248293	19.40144479	44.80519481	18.98640297
151310	F943	L	F	26	FRONT	9.204819277	12.86102511	17.78562984	12.38971998	31.93277311	6.538724351
151310	F943	L	F	26	HIND	7.945541792	11.75000502	15.21220286	12.88109756	9.109730849	4.956022368
151310	F943	L	F	28	FRONT	16.96594427	3.468565312	3.706589321	18.81977671	21.05263158	1.854493581
151310	F943	L	F	28	HIND	14.02398401	18.5437566	12.18994043	8.579545455	51.42857143	11.12396767
151310	F943	L	F	96	FRONT	13.81381381	17.98229365	30.17022716	15.38461538	55.84415584	23.84597541
151310	F943	L	F	96	HIND	24.62462462	26.64131401	37.12744106	38.18181818	3.96508E-14	13.88316151
152686	F1158	L	F	1	FRONT	12.6984127	9.73524931	21.01763365	17.85714286	28.57142857	5.188141391
152686	F1158	L	F	1	HIND	133.3333333	212.9326383	206.0317783	164.9122807	13.33333333	70.12987013
152686	F1158	L	F	2	FRONT	10.15151515	8.521187793	32.65593114	26.51515152	43.93939394	16.52020373
152686	F1158	L	F	2	HIND	9.779411765	30.76739176	24.5884314	11.13172542	35.78947368	9.793958529
152686	F1158	L	F	4	FRONT	11.12497771	14.58530802	21.73796959	13.1778058	5.263157895	2.072229141
152686	F1158	L	F	4	HIND	7.724046872	18.76855668	8.789355245	7.577268195	8.333333333	3.697779022
152686	F1158	L	F	6	FRONT	5.594405594	9.605688001	23.7405728	10.99415205	19.04761905	7.030446798
152686	F1158	L	F	6	HIND	14.33691756	10.17679064	10.03378444	20.63492063	21.11111111	6.317411402
152686	F1158	L	F	8	FRONT	4.203446826	7.922313155	9.350545834	11.95286195	14.21568627	7.760798813
152686	F1158	L	F	8	HIND	11.47172892	5.285604233	12.62368661	19.44444444	4.347826087	8.018600951
152686	F1158	L	F	24	FRONT	11.35443228	14.11852161	9.92238107	8.888888889	24.34782609	4.32070791
152686	F1158	L	F	24	HIND	4.95049505	15.26379706	16.86338378	4	7.692307692	0.952380952
152686	F1158	L	F	26	FRONT	8.641975309	22.20373259	14.83209411	7.775175644	25.78947368	8.245617878
152686	F1158	L	F	26	HIND	10.32388664	15.56926011	22.30316154	15.33779671	9.109730849	5.033316758
152686	F1158	L	F	28	FRONT	3.837719298	1.553730692	2.835176547	11.54918359	34.28571429	7.720149727
152686	F1158	L	F	28	HIND	12.82253127	12.64271387	23.68765911	22.31598687	38.33333333	15.47222069
154983	F1571	L	F	4	FRONT	41.31662982	35.94349874	20.42102178	53.40167754	63.63636364	15.96740544

154983	F1571	L	F	4	HIND	43.48198971	34.7192286	76.82993499	46.05263158	36.66666667	2.721165034
154983	F1571	L	F	6	FRONT	19.37686726	44.30955651	36.5510378	21.59090909	25.92592593	7.587021469
154983	F1571	L	F	6	HIND	25.76286979	61.81815786	57.84367069	44.9657869	6.666666667	21.19939226
154983	F1571	L	F	8	FRONT	52.01328352	41.79557192	54.11437811	48.14814815	42.59259259	16.782271
154983	F1571	L	F	8	HIND	38.63157895	32.53736005	59.43177889	55.36653263	29.09090909	19.03171796
159811	F1541	L	M	1	FRONT	15.5728114	20.762294	37.62088736	23.4520927	43.33333333	10.5296139
159811	F1541	L	M	1	HIND	22.85085305	25.55539594	15.01520769	30.57097542	62.96296296	18.34108875
159811	F1541	L	M	2	FRONT	5.3747774	7.270364308	20.48537953	16.66666667	35.78947368	13.64477489
159811	F1541	L	M	2	HIND	43.9173993	61.2899079	48.36676448	50.98039216	46.59090909	9.320316817
159811	F1541	L	M	4	FRONT	2.43902439	11.42107893	20.08778284	13.125	28.57142857	10.68649886
159811	F1541	L	M	4	HIND	14.83516484	11.47609156	6.381504031	14.63815789	14.28571429	4.686215401
159811	F1541	L	M	6	FRONT	24.96532594	14.20898548	7.35351422	28.94655545	30	8.681383291
159811	F1541	L	M	6	HIND	15.87078652	19.2037471	16.72052389	22.68133842	28.28947368	11.05447944
159811	F1541	L	M	8	FRONT	5.714285714	20.23219686	9.188708368	22.47997791	44.39359268	16.78873239
159811	F1541	L	M	8	HIND	14.23987777	29.57837943	26.91389929	6.382978723	47.40740741	18.02967205
159811	F1541	L	M	24	FRONT	8.412698413	19.46930281	23.85936307	10.8995894	19.71014493	8.213899365
159811	F1541	L	M	24	HIND	19.11175828	17.69861444	16.74486327	21.5892054	32.14285714	11.48300974
159811	F1541	L	M	26	FRONT	8.11965812	1.962934747	5.100254157	3.86002886	26.66666667	4.264177205
159811	F1541	L	M	26	HIND	14.64953271	4.420947036	9.156573928	11.11111111	23.07692308	3.552631579
159811	F1541	L	M	28	FRONT	3.333333333	11.8495573	7.0965912	4.382284382	10.02506266	2.588070612
159811	F1541	L	M	28	HIND	13.44322344	10.69912114	18.68986075	41.74228675	33.61823362	28.58431321
159811	F1541	L	M	96	FRONT	10.4993598	8.719813808	8.625608294	7.417582418	39.59899749	10.78921889
159811	F1541	L	M	96	HIND	7.331628303	1.740740932	14.78992086	27.18346253	37.33333333	22.75170494
159828	F1546	L	F	0	FRONT	44.97574498	34.17303899	50.07350826	51.51515152	29.71014493	7.171656925
159828	F1546	L	F	0	HIND	44.56893869	31.88542384	44.6515555	53.04761905	32.45614035	9.270555603
159828	F1546	L	F	1	FRONT	1.886792453	7.69544542	19.64853737	16.99449253	35.82887701	18.86020804
159828	F1546	L	F	1	HIND	24.26356589	26.20779312	5.501613488	7.692307692	51.17647059	16.78787879
159828	F1546	L	F	2	FRONT	2.325581395	33.08420759	6.256469095	7.969303424	12.54901961	5.651737462
159828	F1546	L	F	2	HIND	29.55766846	77.36837534	71.40276656	31.85904351	20	2.388217226
159828	F1546	L	F	4	FRONT	44.25438596	11.83992902	91.13101495	85.43859649	56.77749361	56.74299764
159828	F1546	L	F	4	HIND	73.17200104	82.72659213	107.7673947	89.58958959	36.11111111	42.77572874
159828	F1546	L	F	6	FRONT	14.70588235	20.63071385	29.64197706	30.20408163	47.95321637	21.53965954
159828	F1546	L	F	6	HIND	28.00269906	49.98647406	44.93367067	30.5764411	20.95238095	2.656948162

159828	F1546	L	F	8	FRONT	6.779661017	6.61117363	9.687288073	14.66905188	10	7.903956109
159828	F1546	L	F	8	HIND	8.210180624	4.807859114	9.865251559	7.827260459	8.333333333	4.018670816
159828	F1546	L	F	24	FRONT	0	14.11883211	13.23651496	6.767676768	20.16806723	6.767676768
159828	F1546	L	F	24	HIND	8.253968254	16.5949771	24.51304361	10.5471325	4.347826087	2.301328035
159828	F1546	L	F	26	FRONT	5	9.819256128	20.9704265	10.42502005	26.73796791	8.531148331
159828	F1546	L	F	26	HIND	4.41712204	1.614540989	6.485613616	3.846153846	4.761904762	2.708862979
159828	F1546	L	F	28	FRONT	6.308610401	7.050269259	3.651806054	5.555555556	19.16666667	7.946517472
159828	F1546	L	F	28	HIND	6.325581395	4.430811495	9.768078194	9.677419355	10.52631579	8.025063261
159828	F1546	L	F	96	FRONT	8.794926004	3.858266705	12.40754589	17.14285714	6.666666667	8.422051375
159828	F1546	L	F	96	HIND	8.863536639	3.908041251	2.969415734	9.090909091	9.523809524	4.014452027
160446	F1546	L	M	0	FRONT	22.68041237	58.36344325	78.68102603	38.88888889	24	16.57393851
160446	F1546	L	M	0	HIND	61.05263158	38.08223474	9.183347425	52.38095238	127.2727273	9.425224734
160446	F1546	L	M	1	FRONT	8.452380952	24.2647089	28.30278633	13.04820587	25	6.515118809
160446	F1546	L	M	1	HIND	8.971600688	7.690010594	21.34022964	6.741940763	16.66666667	2.231384366
160446	F1546	L	M	2	FRONT	16.11253197	16.03758784	9.366815462	14.19098143	25.88235294	1.939884226
160446	F1546	L	M	2	HIND	10.07255655	19.58779156	30.36601725	11.93158114	15.78947368	7.935506806
160446	F1546	L	M	4	FRONT	7.692307692	2.214509798	6.966985661	3.185493752	40	7.112654787
160446	F1546	L	M	4	HIND	13.23877069	7.007253665	14.64610148	17.85714286	23.61111111	9.010250668
160446	F1546	L	M	6	FRONT	7.662447257	2.850616378	7.3872537	6.612948041	11.11111111	1.777150747
160446	F1546	L	M	6	HIND	10.1972102	6.901289527	8.482539138	6.958073149	19.09090909	3.244618152
160446	F1546	L	M	8	FRONT	9.160559627	2.370486962	3.981679889	11.22702434	14.35897436	2.069108697
160446	F1546	L	M	8	HIND	4.651162791	12.44120225	12.43557377	10.63321386	22.61904762	8.834905529
160446	F1546	L	M	24	FRONT	5.376344086	1.731043173	5.270611968	16.6061706	41.91176471	11.28526646
160446	F1546	L	M	24	HIND	3.616734143	4.862516182	10.14262164	4.326494202	10.52631579	2.448950089
160446	F1546	L	M	26	FRONT	6.132756133	15.47895858	9.423789952	3.620196888	36.30952381	9.74760526
160446	F1546	L	M	26	HIND	4.301470588	4.26886146	20.19425498	19.46360153	38.59649123	17.00096612
160446	F1546	L	M	28	FRONT	3.833333333	3.194954022	9.006661117	3.225806452	7.692307692	2.059725585
160446	F1546	L	M	28	HIND	1.265822785	4.531889962	9.562905476	4.920634921	10	3.654876742
160446	F1546	L	M	96	FRONT	10.54964539	10.44885828	13.30662855	18.01742561	23.52941176	7.513376573
160446	F1546	L	M	96	HIND	5.307950728	3.338655416	6.028269395	15.04761905	27.78947368	9.762722429
160639	F1546	L	F	0	FRONT	17.21212121	51.4471148	67.77626096	9.523809524	67.13286713	7.758852795
160639	F1546	L	F	0	HIND	71.32275132	73.90278185	76.08348281	74.24829754	23.07692308	5.752798639
160639	F1546	L	F	1	FRONT	35.27204503	14.46421714	24.55619526	54.28413488	22.61904762	19.83021013

160639	F1546	L	F	1	HIND	25.52845528	14.54547456	12.95699236	37.86816269	14.28571429	12.66955751
160639	F1546	L	F	2	FRONT	34.9825784	12.04565251	13.97086959	45.75305292	26.66666667	12.76571126
160639	F1546	L	F	2	HIND	3.818089258	20.73360041	6.143989741	5.037481259	18.28571429	4.66144573
160639	F1546	L	F	4	FRONT	36.0135311	54.28170323	49.7486362	54.72222222	45.83333333	20.05630327
160639	F1546	L	F	4	HIND	11.10889111	16.16249243	18.66484395	5.831363278	24.15458937	5.286698828
160639	F1546	L	F	6	FRONT	3.571428571	23.11289309	26.31504286	11.52993348	35.78947368	15.09613102
160639	F1546	L	F	6	HIND	5.65920398	14.5575213	27.36774487	15.47619048	26.31578947	12.79873922
160639	F1546	L	F	8	FRONT	16.2037037	8.685527325	11.53490127	9.090909091	30	7.151979566
160639	F1546	L	F	8	HIND	7.738095238	6.754728859	26.67322918	12.22222222	8.94346E-14	4.495035359
160639	F1546	L	F	24	FRONT	10.12345679	3.625393272	11.26233054	2.941176471	31.27272727	7.197870654
160639	F1546	L	F	24	HIND	8.235294118	6.929762749	5.10157854	11.41581633	8.761904762	3.189389872
160639	F1546	L	F	26	FRONT	9.679821296	5.107971393	11.5538781	9.090909091	17.64705882	7.023274806
160639	F1546	L	F	26	HIND	8.890637293	8.362262249	15.11815191	13.89003721	9.109730849	6.533473178
160639	F1546	L	F	28	FRONT	13.24786325	8.176143464	10.08235379	15.38461538	17.69230769	5.593659113
160639	F1546	L	F	28	HIND	9.790491539	15.36624922	14.27266453	19.33395005	17.98941799	9.578680243
160639	F1546	L	F	96	FRONT	51.92307692	5.287858101	14.78987375	59.40144479	31.57894737	9.923581961
160639	F1546	L	F	96	HIND	48.14814815	10.94306609	5.977881082	66.78981938	24.28571429	25.23605525
151303	F943	N	F	0	FRONT	32.79136691	25.25686652	35.04368483	37.19512195	2.31296E-13	4.597078281
151303	F943	N	F	0	HIND	78.76638728	96.79712175	108.7992215	91.66666667	5.263157895	17.86550164
151303	F943	N	F	1	FRONT	18.53815261	39.95438774	34.19392505	13.74458874	54.48916409	10.97787553
151303	F943	N	F	1	HIND	62.42424242	22.26570857	34.54339888	65.0931677	53.96825397	5.087875562
151303	F943	N	F	2	FRONT	12.40854551	18.1716375	30.83676599	21.07142857	17.76315789	8.701168374
151303	F943	N	F	2	HIND	17.467582	28.23151983	55.86477483	39.65381589	32.5	22.59054452
151303	F943	N	F	4	FRONT	21.45344788	5.717581597	10.01651535	22.6984127	17.64705882	6.223606758
151303	F943	N	F	4	HIND	13.02521008	2.70512119	16.26243379	20.19230769	15.88235294	7.219152855
151303	F943	N	F	6	FRONT	14.63414634	22.84297783	25.57251158	17.97990481	14.76190476	6.725840251
151303	F943	N	F	6	HIND	9.948652118	27.93952696	26.50273037	17.96151105	29.74358974	8.071001429
151303	F943	N	F	8	FRONT	4.813108039	4.298806613	2.329253605	9.109730849	25.88235294	10.73789106
151303	F943	N	F	8	HIND	5.882352941	9.675515996	2.50546668	18.0108857	55.07246377	23.84735835
151303	F943	N	F	24	FRONT	16.11445783	13.58902865	17.84221428	12.8342246	28.95927602	3.314323858
151303	F943	N	F	24	HIND	7.142857143	7.592681726	21.62456653	15.69264069	20.2020202	8.593262633
151303	F943	N	F	26	FRONT	15.42002301	11.54976913	9.160561356	17.34627832	5.263157895	1.943261126
151303	F943	N	F	26	HIND	7.399057184	28.13567233	10.67449234	6.364404393	45.12820513	4.807018619

151303	F943	N	F	28	FRONT	28.13852814	25.17564994	9.655929133	24.7047709	43.06220096	3.491118841
151303	F943	N	F	28	HIND	11.55511156	6.829292751	17.07637178	9.375	19.54887218	2.188668936
151303	F943	N	F	96	FRONT	2.564102564	6.923039832	15.48259579	4.615384615	7.692307692	2.05371248
151303	F943	N	F	96	HIND	7.648801508	25.22995868	27.4815125	8.615384615	32.46753247	13.70805638
151307	F943	N	F	0	FRONT	66.75020886	39.4387373	60.7649678	65.63255439	73.5	1.528795767
151307	F943	N	F	0	HIND	128.114892	135.0824011	116.8916528	135.4844382	61.66666667	15.5663193
151307	F943	N	F	1	FRONT	12.46550885	17.33025195	36.64081548	31.35964912	51.07692308	19.13328975
151307	F943	N	F	1	HIND	65.66210046	76.07675611	75.8001434	66.3312369	60	27.39634382
151307	F943	N	F	2	FRONT	8.929045974	12.14656878	18.85035188	16.78321678	28.57142857	11.62707306
151307	F943	N	F	2	HIND	1.694915254	14.28995828	32.54391799	4.143763214	16.84782609	5.837094348
151307	F943	N	F	4	FRONT	11.65845649	18.05577653	16.57057535	12.78385198	25.33936652	10.07559897
151307	F943	N	F	4	HIND	13.78178835	11.64426225	13.96303794	33.51351351	14.28571429	19.92170509
151307	F943	N	F	6	FRONT	12.23162004	21.36417311	18.45689076	7.326007326	39.28571429	10.0593038
151307	F943	N	F	6	HIND	7.081339713	20.89340114	32.30304966	14.28571429	27.69230769	10.90909091
151307	F943	N	F	8	FRONT	10.4679803	24.83801966	23.62766327	13.04347826	29.16666667	9.774151718
151307	F943	N	F	8	HIND	11.11111111	49.29051034	66.20945514	34.7826087	44.3438914	23.69991475
151307	F943	N	F	24	FRONT	4.177489177	13.32835694	21.36066028	0.689655172	12.5	3.655026632
151307	F943	N	F	24	HIND	0	27.47709209	47.62642242	25.33625274	60.2006689	25.33625274
151307	F943	N	F	26	FRONT	6.060606061	1.871756458	8.704608953	12.24489796	21.67182663	6.184522226
151307	F943	N	F	26	HIND	9.741784038	14.85640234	4.67942134	14.3707483	8.695652174	4.646628037
151307	F943	N	F	28	FRONT	4.87012987	6.664051553	10.30361497	14.28571429	35.23809524	12.06793207
151307	F943	N	F	28	HIND	23.54920101	17.63901608	33.28122475	39.39393939	23.37662338	16.24809436
151307	F943	N	F	96	FRONT	10.84529506	1.867493811	8.502881165	5.157962605	47.27272727	9.957945081
151307	F943	N	F	96	HIND	4.07996736	10.04296728	21.77572568	12.14285714	12.54901961	8.073754746
152750	F1571	N	F	0	FRONT	66.61600811	112.3897935	167.806519	73.29192547	43.50877193	7.55309998
152750	F1571	N	F	0	HIND	16.88205585	11.33505252	61.6929282	61.53478935	119.5652174	48.31844772
152750	F1571	N	F	1	FRONT	95.40229885	71.35731404	99.1236781	110.6408169	45.6043956	29.68083503
152750	F1571	N	F	1	HIND	43.34507276	49.68468219	53.95512411	38.56294744	89.8989899	5.03829329
152750	F1571	N	F	2	FRONT	13.38141026	17.08431824	16.33177546	24.12698413	46.31578947	17.10657707
152750	F1571	N	F	2	HIND	26.63414634	30.37730903	42.12906104	16.58268438	53.51170569	10.16705022
152750	F1571	N	F	4	FRONT	29.80816527	10.30176574	5.881463745	31.66915052	22.45614035	2.69883774
152750	F1571	N	F	4	HIND	24.31941924	48.96699382	34.72481042	26.78571429	32.5	5.132583367
152750	F1571	N	F	6	FRONT	81.08108108	6.527349476	6.633589575	100	40	23.72881356

152750	F1571	N	F	6	HIND	90.90909091	72.51357821	126.0663808	136	31.57894737	65.26315789
152750	F1571	N	F	8	FRONT	19.34346772	19.62839665	7.020796726	7.290803645	62.74509804	14.92784961
152750	F1571	N	F	8	HIND	14.22558923	13.57379612	15.45225422	10.31982942	20.55137845	5.207716051
152750	F1571	N	F	24	FRONT	7.209884266	13.66409874	28.07511487	2.479338843	33.33333333	4.733741594
152750	F1571	N	F	24	HIND	17.9144385	15.67719197	12.97229333	22.16705607	25.57471264	8.922961456
152750	F1571	N	F	26	FRONT	8.709032159	13.35031526	7.642956318	19.48717949	33.97129187	13.80744349
152750	F1571	N	F	26	HIND	6.055619549	17.20016989	25.42467006	10.52631579	8.761904762	6.441725804
152750	F1571	N	F	28	FRONT	12.32704403	19.24653524	9.922830406	15.87301587	16.99346405	7.396060093
152750	F1571	N	F	28	HIND	30.37433155	12.66326801	4.893104434	42.42424242	9.090909091	12.58426966
152750	F1571	N	F	96	FRONT	5.505952381	15.48231317	4.490364332	10.44776119	40.52287582	11.21192554
152750	F1571	N	F	96	HIND	2.5	29.83686556	27.44587276	10.45003814	24.34782609	9.441593376
152776	F1571	N	M	0	FRONT	20.05649718	24.52445218	17.44830384	37.25490196	8.333333333	18.17950475
152776	F1571	N	M	0	HIND	44.28571429	62.18116155	64.18906153	57.50350631	50.79365079	25.48507994
152776	F1571	N	M	1	FRONT	3.666121113	17.01726836	20.4794522	12.74961598	21.73913043	12.14498742
152776	F1571	N	M	1	HIND	10.20408163	20.03602793	29.0288426	7.071960298	33.33333333	10.84747485
152776	F1571	N	M	2	FRONT	52.25455708	19.54349355	29.77973335	54.54545455	35.23809524	4.244244244
152776	F1571	N	M	2	HIND	12.50956807	18.88793082	10.07757602	20.78740157	12.40601504	9.765823428
152776	F1571	N	M	4	FRONT	16.4657202	29.26118694	36.47504495	12.614484	38.88888889	3.864540073
152776	F1571	N	M	4	HIND	35.79831933	38.89246096	84.73875648	64.63768116	57.5	30.94964344
152776	F1571	N	M	6	FRONT	6.211180124	22.79918175	20.62848691	13.59649123	14.28571429	7.394894667
152776	F1571	N	M	6	HIND	30.79340141	34.31161152	44.5713672	32.04545455	41.73913043	10.28133508
152776	F1571	N	M	8	FRONT	12.64679313	8.727005131	14.84677645	8.193979933	36.84210526	5.759689059
152776	F1571	N	M	8	HIND	5.499343477	12.14262019	12.3376623	8.376362593	45.83333333	6.646382728
152776	F1571	N	M	24	FRONT	57.43589744	7.636829098	10.64385447	64.05023548	12.5	10.16046967
152776	F1571	N	M	24	HIND	51.10602593	0.73387245	7.385093543	59.03122264	4.347826087	9.769133316
152776	F1571	N	M	26	FRONT	14.35692921	15.13260912	21.73723784	18.9258312	13.57466063	4.619970194
152776	F1571	N	M	26	HIND	14.33691756	3.905553452	2.552832166	21.19565217	21.59090909	6.893682772
152776	F1571	N	M	28	FRONT	1.492537313	4.864784421	10.05753911	6.857142857	24.31372549	8.346403
152776	F1571	N	M	28	HIND	6.644880174	4.920162831	9.947906532	10.59782609	0	3.960654881
152776	F1571	N	M	96	FRONT	8.847320526	6.684133046	14.44656435	5.26525728	33.04347826	5.422415606
152776	F1571	N	M	96	HIND	12.86516854	10.81980575	15.82020404	29.76796831	32.17592593	17.06564132
154850	F998	N	F	0	FRONT	36.76814988	34.77908077	24.3246236	54.34782609	30	18.70254881
154850	F998	N	F	0	HIND	92.80397022	103.9062806	86.48551854	94.59951456	53.80952381	43.81200266

154850	F998	N	F	1	FRONT	65	49.53498975	61.17962169	83.37428337	31.11111111	21.59454541
154850	F998	N	F	1	HIND	15.86445899	27.80036468	25.98327805	15.2027027	65.44117647	25.38034235
154850	F998	N	F	2	FRONT	17.26190476	11.12065712	3.900697179	20.55576703	16.78321678	3.318949369
154850	F998	N	F	2	HIND	25.86734694	28.3687319	21.21915248	19.96512642	44.44444444	6.030860169
154850	F998	N	F	4	FRONT	11.22807018	17.78321513	12.55373239	14.66666667	15.88235294	7.020540249
154850	F998	N	F	4	HIND	17.29323308	20.75601413	26.84172183	15.06883605	21.73913043	9.21537456
154850	F998	N	F	6	FRONT	31.43536875	25.17252795	6.282013147	16.33333333	70.83854819	29.23705443
154850	F998	N	F	6	HIND	2.957164601	5.767741156	11.99105373	15.21335807	24.12280702	12.27102613
154850	F998	N	F	8	FRONT	47.3212883	65.70931809	73.70837405	51.79361179	47.61904762	7.405951605
154850	F998	N	F	8	HIND	3.226544622	12.71639191	23.74097037	7.462686567	23.37662338	8.589007383
154850	F998	N	F	24	FRONT	11.32376396	7.724797887	3.109598949	4.347826087	30	6.980463047
154850	F998	N	F	24	HIND	1.449275362	5.144892542	21.98654964	2.040816327	1.05976E-13	0.591715976
154850	F998	N	F	26	FRONT	10	2.958638162	5.842467452	4.545454545	25	5.479452055
154850	F998	N	F	26	HIND	2.777777778	5.619481085	14.22085672	10.37414966	33.04347826	13.14919736
154850	F998	N	F	28	FRONT	0	11.14185821	11.88585448	2.040816327	4.761904762	2.040816327
154850	F998	N	F	28	HIND	3.53335364	12.18320713	28.10818441	3.225806452	4.347826087	2.678308064
154850	F998	N	F	96	FRONT	3.03657695	8.575356575	5.951185556	3.703703704	11.42857143	3.842940685
154850	F998	N	F	96	HIND	5.714285714	17.60854041	6.512904898	18.36734694	23.80952381	12.78727166
155005	F1158	N	M	0	FRONT	76.26790554	50.95167514	116.9050469	88.96011396	102.9239766	22.03587806
155005	F1158	N	M	0	HIND	74.50980392	40.49290901	43.77323786	82.37934905	57.64411028	15.87446552
155005	F1158	N	M	1	FRONT	42.5463336	18.00670762	38.94531593	56.66666667	28.34008097	16.41275645
155005	F1158	N	M	1	HIND	48.85654886	5.972913754	22.01961185	56.1423221	6.666666667	9.431199279
155005	F1158	N	M	2	FRONT	0	12.83663499	16.47585353	5.263157895	16.66666667	5.263157895
155005	F1158	N	M	2	HIND	21.21699196	45.09573067	29.57543095	34.28571429	20	13.33006616
155005	F1158	N	M	4	FRONT	5.532212885	19.03610946	26.31845906	2.564102564	14.28571429	4.175050302
155005	F1158	N	M	4	HIND	14	26.9282726	42.70751665	10.6442577	22.22222222	7.149950348
155005	F1158	N	M	6	FRONT	13.0212537	20.18087666	17.14220622	22.05882353	56.66666667	9.100939934
155005	F1158	N	M	6	HIND	13.65126677	33.27903403	35.14202989	21.32867133	5.882352941	7.74832138
155005	F1158	N	M	8	FRONT	32.72727273	35.27028013	29.84187851	25.20458265	62.5	7.808363296
155005	F1158	N	M	8	HIND	18.98039216	29.91619967	32.3908734	34.00809717	47.69230769	15.45641217
155005	F1158	N	M	24	FRONT	4.87804878	13.12237175	4.101757586	9.788267963	13.33333333	4.919662506
155005	F1158	N	M	24	HIND	10.6504065	12.65092327	12.53796799	11.47540984	14.76190476	7.52688172
155005	F1158	N	M	26	FRONT	14.44444444	5.891537594	6.495061678	8.992248062	28.10457516	5.486145687

155005	F1158	N	M	26	HIND	7.462686567	0.826758671	16.14583763	22.73901809	23.80952381	15.30683674
155005	F1158	N	M	28	FRONT	5.699088146	12.20167166	4.168461967	6.25	19.16666667	7.699258132
155005	F1158	N	M	28	HIND	7.893569845	8.067770781	10.34781442	8.571428571	7.692307692	5.570548399
155005	F1158	N	M	96	FRONT	14.21568627	20.215296	6.295987693	5.960784314	35.82887701	8.273923085
155005	F1158	N	M	96	HIND	9.100758397	18.77101189	2.511224334	6.207482993	41.84782609	12.47770392
155029	F1571	N	F	0	FRONT	99.50738916	71.09897092	60.29480507	98.92473118	105.8823529	0.772851823
155029	F1571	N	F	0	HIND	133.3333333	185.3822709	178.5651977	129.8245614	155.5555556	6.18556701
155029	F1571	N	F	1	FRONT	15.29411765	10.82643406	25.29483202	24.25164891	30.95238095	9.127996549
155029	F1571	N	F	1	HIND	20.23972603	23.94356209	39.96302209	5.703586346	62.22222222	20.40118546
155029	F1571	N	F	2	FRONT	9.733124019	18.5359514	9.90467333	14.32874355	17.77777778	4.621894562
155029	F1571	N	F	2	HIND	45.38828851	28.7874836	25.48262557	46.40292149	36.11111111	10.48210904
155029	F1571	N	F	4	FRONT	22.66375546	69.12967269	105.5070211	30.56154282	5.263157895	8.034976754
155029	F1571	N	F	4	HIND	34.50655625	7.299223551	15.86136637	44.53519097	13.57466063	10.53147289
155029	F1571	N	F	8	FRONT	41.52080344	17.1068915	20.56460902	59.61030798	82.11143695	19.56322504
155029	F1571	N	F	8	HIND	40.11266094	14.62945595	13.20565947	43.68491322	56.31578947	3.717961833
155029	F1571	N	F	24	FRONT	7.03030303	17.80380107	11.58663327	11.62947938	21.97802198	4.608294931
155029	F1571	N	F	24	HIND	13.75605646	13.45373799	14.11803311	18.74178404	5.263157895	5.089138211
155029	F1571	N	F	26	FRONT	55.35714286	7.064860101	3.398897732	65.11123169	22.05128205	12.97767921
155029	F1571	N	F	26	HIND	60.19585253	21.37739803	34.41042195	76.61233168	21.63009404	26.36570731
155029	F1571	N	F	28	FRONT	20.31719989	7.359451868	2.566877308	35.66037736	27.88461538	16.01455939
155029	F1571	N	F	28	HIND	11.34020619	10.35364518	5.262584195	25.72502685	37.33333333	14.72551106
155029	F1571	N	F	96	FRONT	28.13852814	1.738579985	5.639635788	31.41025641	13.85281385	3.371127077
155029	F1571	N	F	96	HIND	19.59050274	17.94748574	9.033793903	32.41025641	35.69230769	13.13655171
155362	F1158	N	M	0	FRONT	73.68421053	33.79501414	21.91780874	110.5263158	73.68421053	46.26086957
155362	F1158	N	M	0	HIND	55.73770492	6.434423429	56.57017054	75.55555556	0	22.14983713
155362	F1158	N	M	1	FRONT	46.01449275	39.63542969	45.43236555	68.68686869	26.66666667	27.72634906
155362	F1158	N	M	1	HIND	23.33762334	14.47516695	4.611037017	38.71428571	29.91452991	15.85156154
155362	F1158	N	M	2	FRONT	21.24163116	60.17284316	43.94034869	13.35972291	100	21.98600523
155362	F1158	N	M	2	HIND	12.32876712	37.25036719	38.2610694	19.15933529	14.97326203	6.907670028
155362	F1158	N	M	4	FRONT	9.733124019	6.37964352	8.202194423	8.585858586	25	7.213907914
155362	F1158	N	M	4	HIND	20	13.55936259	10.44666848	30.66666667	12.54901961	11.04225352
155362	F1158	N	M	6	FRONT	111.9791667	86.42261758	95.6040189	117.8279975	75.55555556	9.709537708
155362	F1158	N	M	6	HIND	78.24933687	52.48221104	73.79503853	94.49838188	15.38461538	20.15608332

155362	F1158	N	M	8	FRONT	9.703703704	12.47495645	24.61264071	14.6899841	50.98039216	14.68609159
155362	F1158	N	M	8	HIND	17.18082582	32.84257297	29.61591746	17.64705882	24.31372549	14.55620629
155362	F1158	N	M	24	FRONT	7.579318449	2.58592315	13.32162526	1.639344262	34.18803419	5.943314374
155362	F1158	N	M	24	HIND	8.475120385	16.56896203	9.070448428	15.90230665	42.42424242	13.15705478
155362	F1158	N	M	26	FRONT	10.71770335	15.77590128	9.519133519	10	36.07843137	10.69200899
155362	F1158	N	M	26	HIND	12.93859649	14.02119228	20.11979054	16.67953668	47.69230769	21.18137821
155362	F1158	N	M	28	FRONT	6.406406406	6.035411742	12.15482739	8.448275862	3.46945E-14	2.044969955
155362	F1158	N	M	28	HIND	1.492537313	16.49226678	21.39337359	9.415768576	13.04347826	7.923575941
160013	F1546	N	M	0	FRONT	26.26262626	1228.479549	1213.831792	92.53731343	112.5	70.56179775
160013	F1546	N	M	0	HIND	36.89320388	67.83216783	135.1677614	53.65853659	28.57142857	17.63826607
160013	F1546	N	M	1	FRONT	6.083916084	15.42670304	8.882877067	5.266622778	23.07692308	8.262395433
160013	F1546	N	M	1	HIND	22.36135957	18.2948052	43.72235721	37.36780259	36.66666667	29.12693638
160013	F1546	N	M	2	FRONT	23.54978355	14.52362786	22.16970378	21.59090909	27.27272727	4.151023374
160013	F1546	N	M	2	HIND	17.57575758	13.43098094	2.429898587	32.26544622	15.88235294	14.97927251
160013	F1546	N	M	4	FRONT	12.94117647	10.3516396	26.54746317	20.54507338	47.27272727	12.30454634
160013	F1546	N	M	4	HIND	8.991008991	13.27114764	1.152391109	33.03167421	38.46153846	24.17034232
160013	F1546	N	M	6	FRONT	22.37762238	14.10969778	10.27771007	35.94202899	33.15508021	13.76565221
160013	F1546	N	M	6	HIND	23.34337349	13.97731274	23.68708146	26.28685657	32.46753247	7.371750108
160013	F1546	N	M	8	FRONT	5.158730159	7.05110875	12.85991724	7.390817469	9.63735E-14	2.235455795
160013	F1546	N	M	8	HIND	3.185493752	7.058536678	10.93117392	7.373737374	14.97326203	6.78876011
160013	F1546	N	M	24	FRONT	7.448275862	8.75294315	9.461002994	25.35612536	31.07769424	18.00828076
160013	F1546	N	M	24	HIND	21.62955713	16.50396741	15.75391918	18.30663616	44.72049689	13.79836304
160013	F1546	N	M	26	FRONT	6.25	7.546939311	19.45349875	15.47388781	39.41176471	15.47195708
160013	F1546	N	M	26	HIND	3.03657695	11.77002128	13.01508445	7.127659574	4.347826087	4.094002516
160013	F1546	N	M	28	FRONT	4.102564103	25.25870861	16.48179442	9.251412429	43.9628483	13.3403623
160013	F1546	N	M	28	HIND	12.71963331	13.3164036	17.3138265	14.17565485	8.333333333	1.460543102
160013	F1546	N	M	96	FRONT	7.874786011	3.093341545	1.686848783	4.225352113	48.05491991	12.09443183
160013	F1546	N	M	96	HIND	6.923597025	4.359633394	8.127730267	3.225806452	16.76190476	3.70247356
160021	F1768	N	M	0	FRONT	49.48002189	77.93975261	118.285624	86.66666667	16.74718196	56.99465067
160021	F1768	N	M	0	HIND	30.90206186	73.84726316	76.88553765	36.96581197	18.66666667	6.307683941
160021	F1768	N	M	1	FRONT	21.56028369	20.51565993	10.8465725	21.0755814	24.31372549	8.31499435
160021	F1768	N	M	1	HIND	3.620196888	28.91052576	27.8244032	17.39130435	41.66666667	20.97727294
160021	F1768	N	M	2	FRONT	47.70191879	19.08525432	18.50417111	60.31909418	4.761904762	13.85026911

160021	F1768	N	M	2	HIND	42.10313448	39.48879012	49.10510342	56.25	15.78947368	15.12115884
160021	F1768	N	M	4	FRONT	9.836065574	11.51381553	24.12775007	16.7405765	5.882352941	6.947049618
160021	F1768	N	M	4	HIND	13.40286832	12.53244784	6.64842559	18.18181818	57.14285714	19.48197238
160021	F1768	N	M	6	FRONT	6.612948041	21.24450569	22.36215996	2.66758E-14	24.76190476	6.612948041
160021	F1768	N	M	6	HIND	4.347826087	4.325953681	9.200397309	20.50739958	25.88235294	16.2698045
160021	F1768	N	M	8	FRONT	8.333333333	5.33723212	10.89811558	12.40487062	25.54347826	7.048092869
160021	F1768	N	M	8	HIND	14.68253968	5.964623221	6.570866905	22.21462748	20.2020202	7.565927397
160021	F1768	N	M	24	FRONT	8.823529412	11.4123077	3.678246129	14.48445172	15.87301587	5.67811847
160021	F1768	N	M	24	HIND	4.825870647	7.886420994	12.56466008	12.74853801	9.090909091	7.948236866
160021	F1768	N	M	26	FRONT	7.573632539	9.947018429	14.618808	10.63348416	32.14285714	11.75730543
160021	F1768	N	M	26	HIND	14.3707483	14.67020351	19.13055175	22.31486996	7.703703704	8.029411734
160021	F1768	N	M	28	FRONT	13.78109453	11.94433867	2.724432464	15.6504065	21.05263158	6.126478778
160021	F1768	N	M	28	HIND	15.4589372	29.00720406	9.343603806	8	64.84210526	23.41613711
160021	F1768	N	M	96	FRONT	10.64030132	6.952001312	4.793917926	10.66037736	10.52631579	0.190114238
160021	F1768	N	M	96	HIND	7.5	8.942234466	3.249925826	24.47174447	24	17.08187709
160096	F1034	N	F	0	FRONT	10.50615595	37.63555335	42.62231071	18.05718762	29.16666667	7.589700286
160096	F1034	N	F	0	HIND	62.70996641	40.95632668	41.89021811	74.35897436	36.36363636	17.38155313
160096	F1034	N	F	1	FRONT	9.090909091	30.08380304	61.53482007	14.34200158	12.95546559	5.266203704
160096	F1034	N	F	1	HIND	6.671608599	21.10912462	24.78308214	6.564102564	36.50793651	13.222117
160096	F1034	N	F	2	FRONT	9.090909091	9.796908768	13.62965919	5.263157895	33.33333333	14.35406699
160096	F1034	N	F	2	HIND	11.01449275	13.9479525	38.95690009	20.19230769	19.54887218	9.227649147
160096	F1034	N	F	4	FRONT	11.89964158	3.693685517	13.58358339	12.97208539	22.5	5.821194962
160096	F1034	N	F	4	HIND	13.55555556	4.350326193	9.986011818	15.14285714	9.090909091	3.317426108
160096	F1034	N	F	6	FRONT	10.43536058	1.669768973	9.073663812	18.61471861	28.10457516	12.08979901
160096	F1034	N	F	6	HIND	5.53652968	5.999821771	7.351167767	11.49068323	11.11111111	8.699360341
160096	F1034	N	F	8	FRONT	12.44292237	9.020511487	17.8391924	14.09888357	17.77777778	6.281303799
160096	F1034	N	F	8	HIND	17.40540541	4.344728361	9.419725103	22.21350078	27.27272727	15.74321656
160096	F1034	N	F	24	FRONT	3.704819277	10.69040834	11.18589555	5.087719298	26.08695652	8.788851615
160096	F1034	N	F	24	HIND	3.643843667	7.042581642	16.256582	7.478559177	3.448275862	3.839303384
160096	F1034	N	F	26	FRONT	8.825438027	7.514129426	2.770148004	14.38297872	5.263157895	5.575749491
160096	F1034	N	F	26	HIND	6.631578947	11.27734482	20.13596916	6.382978723	6.666666667	5.020657446
160096	F1034	N	F	28	FRONT	6.651524984	18.58342608	17.75188715	10.89700997	34.84848485	13.20557512
160096	F1034	N	F	28	HIND	2.873912164	2.351216381	19.92454284	8.108108108	12.03703704	7.711941459

160096	F1034	N	F	96	FRONT	1.587301587	3.468609927	8.278579568	7.464607465	10.6442577	5.879707241
160096	F1034	N	F	96	HIND	3.703703704	10.00564269	6.136809436	5.469327421	17.98941799	9.165481789
160121	F1546	N	F	0	FRONT	4.761904762	64.3744532	11.81031834	14.92537313	82.35294118	19.65235899
160121	F1546	N	F	0	HIND	12.98701299	66.80968879	61.70301377	28.57142857	85.71428571	41.17647059
160121	F1546	N	F	1	FRONT	8.395802099	10.48157953	9.876993632	9.15275201	24.28571429	5.307209404
160121	F1546	N	F	1	HIND	13.03731696	16.44682695	21.32728696	17.75599129	10.52631579	5.808695016
160121	F1546	N	F	2	FRONT	37.04367301	7.347933161	8.439932278	12.70772239	89.22413793	24.59582752
160121	F1546	N	F	2	HIND	24.48512586	15.35698746	27.39949139	52.20338983	41.17647059	28.79765786
160121	F1546	N	F	4	FRONT	3.50877193	11.52018768	16.25772002	8.695652174	18.18181818	5.190839695
160121	F1546	N	F	4	HIND	17.64705882	11.25072624	16.98113204	38.29787234	28.57142857	21.00572884
160121	F1546	N	F	6	FRONT	3.896103896	4.989855008	7.427906976	4.904761905	9.109730849	1.658461062
160121	F1546	N	F	6	HIND	3.349156118	13.58778178	2.310231404	8.976833977	22.13438735	8.160882884
160121	F1546	N	F	8	FRONT	25.64102564	11.18607102	6.672607616	27.59535655	39.77272727	2.508755174
160121	F1546	N	F	8	HIND	8.789659224	35.46079718	29.02012463	18.36925961	17.77777778	9.613461262
160121	F1546	N	F	24	FRONT	4.482758621	3.947899938	3.451271847	2.43902439	10.52631579	2.044461902
160121	F1546	N	F	24	HIND	3.03030303	7.586650117	16.10774473	14.26978818	15.11111111	11.2776685
160121	F1546	N	F	26	FRONT	6.389452333	21.92076619	17.56447844	12.10810811	4.761904762	5.732967868
160121	F1546	N	F	26	HIND	4	17.14024378	6.829365083	8.03030303	25.33936652	12.02179177
160121	F1546	N	F	28	FRONT	3.456221198	12.04424178	8.571362183	10.92922484	16.78321678	7.480905094
160121	F1546	N	F	28	HIND	5.617977528	9.173157502	8.488405465	6.254728878	13.0952381	2.644543569
160121	F1546	N	F	96	FRONT	12.20682303	9.03415454	4.673261807	15.76402321	2.77556E-14	3.60072764
160121	F1546	N	F	96	HIND	16.29318394	3.779108306	11.45808837	25.26315789	16.34782609	12.29928669
160153	F1768	N	M	0	FRONT	79.71130714	52.27800314	144.756792	104.2857143	12.54901961	30.80985915
160153	F1768	N	M	0	HIND	10.79365079	39.99853725	60.4562514	30.72100313	35.57692308	24.81509237
160153	F1768	N	M	1	FRONT	3.822574829	23.60653352	21.22637467	4.761904762	28.75816993	8.579272478
160153	F1768	N	M	1	HIND	13.79310345	19.83761751	21.14443078	26.64437012	42.98245614	16.25489257
160153	F1768	N	M	2	FRONT	90.44397463	6.223988415	9.742609122	105.5615844	23.07692308	20.6829934
160153	F1768	N	M	2	HIND	21.04578881	20.91894258	14.44295958	23.61655773	52.5	6.424812987
160153	F1768	N	M	4	FRONT	9.645996674	6.489471863	10.82061323	14.31818182	21.97802198	7.614422504
160153	F1768	N	M	4	HIND	12.31060606	16.37458315	7.321138079	12.5	44.44444444	12.73854962
160153	F1768	N	M	6	FRONT	15.38461538	28.00000066	19.05071635	18.18181818	0	2.816901408
160153	F1768	N	M	6	HIND	69.87951807	107.1428563	105.8823525	78.26086957	28.57142857	9.708737864
160153	F1768	N	M	8	FRONT	4.347826087	12.19731423	10.89821917	3.703703704	15.78947368	8.038585209

160153	F1768	N	M	8	HIND	4.479840717	22.66552241	9.878348001	11.55913978	28.75816993	16.01366499
160153	F1768	N	M	24	FRONT	11.93396226	15.16015344	19.45112088	11.13841114	12.5	1.866779078
160153	F1768	N	M	24	HIND	16.12818492	15.1927876	13.97103085	12.5	25.88235294	3.673592291
160153	F1768	N	M	26	FRONT	8.193979933	16.20294494	17.9358096	11.14058355	25.33936652	11.6419979
160153	F1768	N	M	26	HIND	8.902821317	19.60530211	10.42512553	23.75533428	34.17366947	16.5255523
160153	F1768	N	M	28	FRONT	9.791666667	8.254286853	10.90250429	13.84408602	3.96508E-14	4.073327802
160153	F1768	N	M	28	HIND	8.857336038	16.44305516	4.126984238	8.67362E-15	25.26315789	8.857336038
160153	F1768	N	M	96	FRONT	24.97897393	16.39677455	15.3163652	18.44827586	41.66666667	15.97114996
160153	F1768	N	M	96	HIND	4.47761194	14.63937025	7.626401013	6.648697215	25.26315789	11.10665061
160777	F1745	N	M	1	FRONT	88.74824191	81.55495371	90.76876313	105.3178729	36.11111111	21.65402589
160777	F1745	N	M	1	HIND	57.55213819	57.74934497	49.90547504	66.94470188	12.5	11.41213515
160777	F1745	N	M	2	FRONT	21.973466	8.539573068	12.291343	22.5311943	55.14705882	13.94497656
160777	F1745	N	M	2	HIND	27.42735648	27.56201751	9.236735702	15.49744898	63.15789474	12.06454496
160777	F1745	N	M	4	FRONT	11.58730159	10.08991986	17.2778507	9.166666667	29.09090909	2.425076241
160777	F1745	N	M	4	HIND	9.407894737	27.77852307	11.5876759	3.624282694	34.92063492	10.42309121
160777	F1745	N	M	6	FRONT	16.66666667	40.68272201	59.78758209	31.57894737	40	15.11111111
160777	F1745	N	M	6	HIND	21.17647059	37.0889833	79.78142138	42.62295082	33.33333333	21.94159432
160777	F1745	N	M	8	FRONT	10.42424242	40.62805169	33.78696847	13.37073399	12.95546559	2.957117223
160777	F1745	N	M	8	HIND	9.495949595	31.21793501	31.56354212	14.24954792	29.29292929	8.168954341
160777	F1745	N	M	24	FRONT	26.035313	16.85537428	10.3782466	26.30985915	20	7.265085661
160777	F1745	N	M	24	HIND	11.46268657	13.21578723	21.60513329	15.81694099	19.09090909	6.539421013
160777	F1745	N	M	26	FRONT	9.253139458	5.136699305	5.182499975	14.97876146	11.11111111	5.743992914
160777	F1745	N	M	26	HIND	12.79761905	8.106326591	7.901756145	11.55407018	16	1.249610858
160777	F1745	N	M	28	FRONT	15.0678733	13.5439261	11.9278968	5.263157895	42.85714286	9.843057386
160777	F1745	N	M	28	HIND	10.26733824	22.3462001	8.436392525	9.523809524	35.57692308	10.26133852
160777	F1745	N	M	96	FRONT	9.344012204	25.1125482	15.85350682	9.215686275	47.22222222	11.89882218
160777	F1745	N	M	96	HIND	13.28671329	24.88648252	29.09434111	9.270516717	23.44497608	4.039757172
