# Supplementary material for: Does intrauterine crowding affect locomotor development? A comparative study of motor performance, neuromotor maturation and gait variability among piglets that differ in birth weight and vitality
Source: PLoS One. 2018 Apr 24;13(4):e0195961. doi: 10.1371/journal.pone.0195961 (PMC5915318; doi:10.1371/journal.pone.0195961)
Supplement: S7 Table — (PDF) [file pone.0195961.s007.pdf]

S7. INTERLIMB VARIABLES

PIGLET	SOW	CATEGORY	GENDER	AGE (h)	FRONT LAG	HIND LAG	PAIR LAG LEFT	PAIR LAG RIGHT	DIA LAG LHRF	DIA LAG RHLF
151301	F1816	L	F	1	0.456896552	0.428323197	0.619318182	0.228448276	0.220522721	0.207009207
151301	F1816	L	F	2	0.412807882	0.365334492	0.40625	0.196599826	0.120879121	0.149038462
151301	F1816	L	F	4	0.426303855	0.264552563	0.918032787	0.45825603	0.562724014	0.547107015
151301	F1816	L	F	6	0.459781529	0.314943527	0.679259259	0.188688689	0.256565657	0.21369248
151301	F1816	L	F	8	0.382953181	0.415725456	0.471998871	0.507775309	0.678411005	0.101487928
151301	F1816	L	F	24	0.465126812	0.390529974	0.665046399	0.246527778	0.202293202	0.199017199
151301	F1816	L	F	26	0.401262916	0.314943527	0.583576933	0.208924949	0.15174443	0.175283733
151301	F1816	L	F	28	0.428333333	0.302345786	0.667693492	0.22	0.19163892	0.250462449
151301	F1816	L	F	96	0.485167464	0.554300608	0.843137255	0.24871001	0.308123249	0.366946779
151302	F1816	L	F	0	0.419047619	0.503909644	0.658333333	0.224821404	0.20158371	0.207918552
151302	F1816	L	F	1	0.243407708	0.352736751	0.324188931	0.088220919	0.12571752	0.112822379
151302	F1816	L	F	2	0.435617345	0.302345786	0.690499834	0.177005013	0.232142857	0.234375
151302	F1816	L	F	4	0.433409874	0.403127715	0.60015528	0.541889483	0.661306043	0.184697856
151302	F1816	L	F	6	0.441558442	0.34013901	0.731481481	0.159739958	0.284188034	0.278727445
151302	F1816	L	F	8	0.552380952	0.415725456	0.787061351	0.30478955	0.289604609	0.244828489
151302	F1816	L	F	24	0.50862069	0.466116421	0.805405405	0.249517271	0.252268603	0.299909256
151302	F1816	L	F	26	0.593957921	0.478714162	0.773173927	0.313189057	0.311085539	0.222477944
151302	F1816	L	F	28	0.485449735	0.415725456	0.790243902	0.183047981	0.290063425	0.296617336
151302	F1816	L	F	96	0.335164835	0.655082537	0.556680162	0.259803922	0.071115288	0.213659148
151306	F1349	L	F	4	0.406321839	0.491311903	0.46743295	0.709687034	1.083333333	0.1
151306	F1349	L	F	6	0.228470588	0.377932233	0.439599086	0.472721823	0.657190083	0.185454545
151309	F943	L	F	1	0.657142857	1.058210252	0.733333333	0.564102564	0.034482759	0.034482759
151309	F943	L	F	2	0.619554205	0.818853171	0.522688337	0.572837266	0.590909091	0.055944056
151309	F943	L	F	4	0.526632788	0.466116421	0.698461538	0.326454034	0.151602564	0.139423077
151309	F943	L	F	6	0.548161765	0.503909644	0.80952381	0.293040293	0.230006609	0.200594845
151309	F943	L	F	8	0.395086841	0.680278019	0.54368471	0.659861168	0.489979706	0.128868595
151309	F943	L	F	24	0.483915396	0.377932233	0.496470588	0.322463768	0.130589431	0.30945122
151309	F943	L	F	26	0.653958944	0.529105126	0.897493876	0.342013214	0.355540751	0.254274928
151309	F943	L	F	28	0.438440492	0.718071242	0.637829457	0.294179104	0.130252101	0.179096639
151310	F943	L	F	0	0.257425743	0.050390964	0.206896552	0.110091743	0.233333333	0.133333333

151310	F943	L	F	1	0.410037879	0.604691573	1.264335664	0.181944444	0.344560114	0.550112268
151310	F943	L	F	2	0.178092784	0.352736751	0.324691358	0.081193838	0.097065064	0.12883074
151310	F943	L	F	4	0.702280912	0.415725456	0.80293501	0.489417989	0.216457961	0.169946333
151310	F943	L	F	6	0.413810071	0.415725456	0.594816054	0.255578093	0.167711599	0.178683386
151310	F943	L	F	8	0.283266705	0.478714162	0.482142857	0.222819783	0.066466767	0.222388806
151310	F943	L	F	24	0.465343728	0.503909644	0.658953998	0.268424611	0.187737385	0.203743896
151310	F943	L	F	26	0.402771084	0.516507385	0.612446959	0.224122207	0.162331355	0.20420939
151310	F943	L	F	28	0.543498452	0.466116421	0.695045695	0.328800389	0.24916388	0.158862876
151310	F943	L	F	96	0.493993994	0.264552563	0.536324786	0.338095238	0.15	0.05
152686	F1158	L	F	1	0.46031746	0.251954822	1.139240506	0.100840336	0.985915493	0.450704225
152686	F1158	L	F	2	0.736363636	0.516507385	0.756079986	0.530778165	0.219341974	0.062612164
152686	F1158	L	F	4	0.588340168	0.453518679	0.701924581	0.333270357	0.26271777	0.16097561
152686	F1158	L	F	6	0.501165501	0.440920938	0.621212121	0.603616813	0.678938356	0.113441781
152686	F1158	L	F	8	0.560739807	0.503909644	0.725829726	0.404436153	0.152258065	0.173978495
152686	F1158	L	F	24	0.461151942	0.655082537	0.752293578	0.212979351	0.28619469	0.273893805
152686	F1158	L	F	26	0.487654321	0.415725456	0.724238026	0.277525675	0.217589428	0.217589428
152686	F1158	L	F	28	0.481359649	0.566898349	0.643539467	0.249331806	0.224453782	0.184033613
154983	F1571	L	F	4	0.488577745	0.088184188	0.317460317	0.34119898	0.18018018	0.191441441
154983	F1571	L	F	6	0.497012377	0.415725456	0.702517162	0.326636051	0.553211439	0.166666667
154983	F1571	L	F	8	0.589248651	0.302345786	0.538137918	0.351984127	0.291362126	0.060797342
159811	F1541	L	M	1	0.349643655	0.440920938	0.500590319	0.191939727	0.164461066	0.164365171
159811	F1541	L	M	2	0.415573903	0.478714162	0.646486668	0.307292707	0.127922078	0.216883117
159811	F1541	L	M	4	0.551942186	0.478714162	0.771043771	0.317647059	0.23406214	0.22301496
159811	F1541	L	M	6	0.396275015	0.466116421	0.5679969	0.27365838	0.129306931	0.099207921
159811	F1541	L	M	8	0.580243161	0.554300608	0.634711779	0.481541883	0.093253968	0.047619048
159811	F1541	L	M	24	0.521031746	0.428323197	0.69023569	0.291530002	0.211764706	0.176470588
159811	F1541	L	M	26	0.524928775	0.655082537	0.786635833	0.294104231	0.234693878	0.265306122
159811	F1541	L	M	28	0.49009009	0.365334492	0.59562212	0.266838267	0.193452381	0.142113095
159811	F1541	L	M	96	0.471007865	0.503909644	0.777670091	0.269927536	0.231092437	0.304621849
159828	F1546	L	F	0	0.582120582	0.440920938	0.560175472	0.2092827	0.513354893	0.202778776
159828	F1546	L	F	1	0.467794405	0.314943527	0.713636364	0.25530504	0.232945736	0.209689922
159828	F1546	L	F	2	0.416750927	0.34013901	0.625	0.209325397	0.224303136	0.173127178
159828	F1546	L	F	4	0.715570175	0.277150304	0.56690233	0.532571182	0.302507837	0.705329154

159828	F1546	L	F	6	0.5	0.415725456	0.867463184	0.241653826	0.298170587	0.276075077
159828	F1546	L	F	8	0.542372881	0.352736751	0.715758858	0.275	0.267651888	0.198686371
159828	F1546	L	F	24	0.501668521	0.34013901	0.709862087	0.219581211	0.274525111	0.225344783
159828	F1546	L	F	26	0.525	0.415725456	0.769264069	0.251147541	0.294748858	0.239954338
159828	F1546	L	F	28	0.494032396	0.314943527	0.772093023	0.267689685	0.233560091	0.253968254
159828	F1546	L	F	96	0.46384778	0.289748045	0.642706131	0.294866169	0.17310789	0.176328502
160446	F1546	L	M	0	0.618556701	0.781059948	0.735632184	0.552380952	0.026315789	0.052631579
160446	F1546	L	M	1	0.49702381	0.57949609	0.598610659	0.382075472	0.11038961	0.127435065
160446	F1546	L	M	2	0.436913896	0.390529974	0.66512605	0.226868906	0.211508248	0.222898665
160446	F1546	L	M	4	0.485284281	0.478714162	0.651242236	0.283264528	0.180289855	0.177826087
160446	F1546	L	M	6	0.570464135	0.541702867	0.73640307	0.38218111	0.186914203	0.175378515
160446	F1546	L	M	8	0.501665556	0.491311903	0.737166992	0.239837398	0.23445122	0.23445122
160446	F1546	L	M	24	0.474571346	0.503909644	0.643369963	0.302851787	0.155833333	0.163333333
160446	F1546	L	M	26	0.476731602	0.453518679	0.656356736	0.312937063	0.158016765	0.162653826
160446	F1546	L	M	28	0.515833333	0.453518679	0.658008658	0.308071675	0.203505355	0.152872444
160446	F1546	L	M	96	0.42087766	0.57949609	0.663272311	0.250244167	0.162440734	0.223459081
160639	F1546	L	F	0	0.313333333	1.347958297	0.76092504	0.395574875	0.576684185	0.360774009
160639	F1546	L	F	1	0.712677566	0.541702867	0.846213094	0.522972206	0.245495495	0.178678679
160639	F1546	L	F	2	0.525783972	0.440920938	0.610834371	0.24168798	0.218689163	0.13259788
160639	F1546	L	F	4	0.449128285	0.478714162	0.763347763	0.202017654	0.254032258	0.434331797
160639	F1546	L	F	6	0.5	0.289748045	0.552941176	0.344671202	0.137254902	0.13029728
160639	F1546	L	F	8	0.490740741	0.314943527	0.732156863	0.245098039	0.223382749	0.221361186
160639	F1546	L	F	24	0.548148148	0.629887055	0.827797203	0.338259442	0.24237013	0.279383117
160639	F1546	L	F	26	0.421072226	0.415725456	0.675961538	0.200379867	0.214772132	0.236423957
160639	F1546	L	F	28	0.599358974	0.541702867	0.755868545	0.449275362	0.149953358	0.181203358
160639	F1546	L	F	96	0.37145749	0.440920938	0.539849624	0.238194786	0.117262496	0.15673618
151303	F943	N	F	0	0.541798523	0.453041049	0.720595992	0.237712895	0.185429654	0.323845328
151303	F943	N	F	1	0.470731707	0.409848485	0.511653117	0.490137328	0.179252846	0.146743295
151303	F943	N	F	2	0.591970121	0.489702517	0.746031746	0.305416964	0.322857143	0.152065081
151303	F943	N	F	4	0.61529884	0.527310924	0.826048171	0.304120879	0.375975474	0.208333333
151303	F943	N	F	6	0.39408707	0.470474968	0.637589344	0.218189833	0.15238764	0.247692308
151303	F943	N	F	8	0.452991453	0.432263815	0.656410256	0.243464052	0.205109127	0.196785304
151303	F943	N	F	24	0.497633782	0.578754579	0.706406989	0.389803922	0.076624473	0.199626866

151303	F943	N	F	26	0.520588235	0.508198401	0.755502846	0.278584392	0.287942478	0.237095363
151303	F943	N	F	28	0.553895072	0.554112554	0.79491256	0.308279221	0.289318304	0.233760495
151303	F943	N	F	96	0.387631976	0.448051948	0.633936652	0.217986315	0.167755991	0.269293924
151307	F943	N	F	0	0.488717666	0.427955831	0.664281783	0.267740012	0.234330887	0.180523256
151307	F943	N	F	1	0.691511387	0.404515461	1.306418219	0.241549592	0.17324628	0.304858156
151307	F943	N	F	2	0.691761364	0.41803653	0.505208333	0.528198908	0.061538462	0.130833333
151307	F943	N	F	4	0.54025974	0.438287701	0.680519481	0.573099415	0.69047619	0.137987013
151307	F943	N	F	6	0.50802139	0.584495488	0.752673797	0.343939394	0.138528139	0.225931677
151307	F943	N	F	8	0.490566038	0.631578947	0.79245283	0.350877193	0.111111111	0.285714286
151307	F943	N	F	24	0.610169492	0.658297258	0.796610169	0.480286738	0.148198971	0.174242424
151307	F943	N	F	26	0.449186992	0.50617284	0.659620596	0.210285863	0.22542749	0.210433604
151307	F943	N	F	28	0.489855072	0.504107981	0.676811594	0.331932773	0.153544849	0.195089286
151307	F943	N	F	96	0.45	0.484440706	0.535087719	0.420571174	0.029462366	0.145604396
152750	F1571	N	F	0	0.476070104	0.487981619	0.644421975	0.311594203	0.167109635	0.171568627
152750	F1571	N	F	1	0.520525452	0.507139943	0.749589491	0.276190476	0.23150267	0.234060762
152750	F1571	N	F	2	0.746551724	0.357917409	0.563793103	0.881587302	0.172910904	0.192208166
152750	F1571	N	F	4	0.272447287	0.215628945	0.416607439	0.137836448	0.172194778	0.110411141
152750	F1571	N	F	6	0.581818182	0.64	0.727272727	0.406779661	0.150943396	0.131147541
152750	F1571	N	F	8	0.410997005	0.489066442	0.633718442	0.232434232	0.121256039	0.198901099
152750	F1571	N	F	24	0.541896024	0.453748006	0.517227319	0.323376623	0.048387097	0.13370341
152750	F1571	N	F	26	0.448721014	0.553451178	0.73129333	0.280172414	0.124901497	0.277344673
152750	F1571	N	F	28	0.438235294	0.582887701	0.726470588	0.289278752	0.134412386	0.281418011
152750	F1571	N	F	96	0.458171371	0.489550765	0.72519858	0.210455564	0.225186104	0.260416667
152776	F1571	N	M	0	0.452731092	0.343582888	0.600840336	0.212820513	0.246704331	0.173469388
152776	F1571	N	M	1	0.489283075	0.497727273	0.705099778	0.281631098	0.212797619	0.218560606
152776	F1571	N	M	2	0.409855769	0.448979592	0.530849359	0.361556982	0.758404746	0.125831202
152776	F1571	N	M	4	0.496163683	0.493877551	0.605498721	0.394029851	0.089220564	0.102941176
152776	F1571	N	M	6	0.477694236	0.5043193	0.713283208	0.296448087	0.118399044	0.219339623
152776	F1571	N	M	8	0.777885235	0.503361345	0.70782291	0.218487395	0.626303128	0.433954451
152776	F1571	N	M	24	0.377338603	0.323016496	0.693280632	0.274958541	0.37515528	0.380035651
152776	F1571	N	M	26	0.333467612	0.450683556	0.586448598	0.195601852	0.12674872	0.245421245
152776	F1571	N	M	28	0.341045182	0.363335876	0.489021956	0.2239819	0.093083315	0.190841055
152776	F1571	N	M	96	0.459510358	0.413381123	0.599811676	0.285895003	0.163925439	0.150219298

154850	F998	N	F	0	0.513315774	0.509259259	0.696224759	0.335738069	0.169340463	0.176920973
154850	F998	N	F	1	0.551001592	0.468913858	0.753247842	0.257936508	0.322802198	0.201808786
154850	F998	N	F	2	0.350949051	0.576923077	1.154245754	0.38697318	0.746666667	0.954042082
154850	F998	N	F	4	0.709558824	0.469772815	0.707107843	0.327438017	0.158107117	0.074772886
154850	F998	N	F	6	0.595468361	0.675255102	0.861287649	0.40091954	0.171333936	0.233433735
154850	F998	N	F	8	0.482954545	0.460150376	0.579545455	0.557028253	0.595604396	0.104174573
154850	F998	N	F	24	0.540246212	0.589258534	0.724905303	0.372139085	0.255555556	0.182307692
154850	F998	N	F	26	0.752305665	0.481922197	0.941040843	0.314144737	0.283228024	0.188052373
154850	F998	N	F	28	0.505582137	0.529710145	0.734449761	0.311594203	0.169047619	0.230499325
154850	F998	N	F	96	0.509790528	0.514705882	0.733151184	0.307675906	0.183665008	0.226932084
155005	F1158	N	M	0	0.531702899	0.504112093	0.793478261	0.240096038	0.29076087	0.260504202
155005	F1158	N	M	1	0.467287597	0.517050691	0.725124943	0.259337068	0.208333333	0.264564275
155005	F1158	N	M	2	0.27643909	0.553391193	0.097222222	0.409375	0.647754137	0.2508519
155005	F1158	N	M	4	0.811428571	0.851091476	0.967261905	0.662912913	0.244530245	0.139640411
155005	F1158	N	M	6	0.413114754	0.349712974	0.432534678	0.392081737	0.064564943	0.125798212
155005	F1158	N	M	8	0.476666667	0.45	0.616666667	0.317577031	0.166556945	0.134615385
155005	F1158	N	M	24	0.568100358	0.584202683	0.759856631	0.375806452	0.175438596	0.179890024
155005	F1158	N	M	26	0.389010989	0.408627451	0.357753358	0.496177648	0.378181818	0.033482143
155005	F1158	N	M	28	0.558641975	0.504878049	0.736419753	0.330702119	0.233549784	0.172413793
155005	F1158	N	M	96	0.441468254	0.489505597	0.614087302	0.335568803	0.10320942	0.183212268
155029	F1571	N	F	0	0.545454545	0.487804878	0.727272727	0.272727273	0.279069767	0.177777778
155029	F1571	N	F	1	0.510198878	0.526248399	0.696328404	0.340829585	0.175668449	0.178333333
155029	F1571	N	F	2	0.514273166	1.185780886	0.605182257	1.148050072	1.160073037	0.084507042
155029	F1571	N	F	4	1.411983033	0.492123288	0.800636267	1.297782037	0.184782609	0.634469697
155029	F1571	N	F	8	0.367647059	0.338862361	0.476470588	0.280828083	0.140180879	0.131028596
155029	F1571	N	F	24	0.238864454	0.732919255	0.854298281	0.125890239	0.110837856	0.514835165
155029	F1571	N	F	26	0.569135802	0.569420601	0.598547567	0.384436783	0	0.027932961
155029	F1571	N	F	28	0.433016771	0.407099221	0.524853506	0.318383284	0.116892911	0.090909091
155029	F1571	N	F	96	0.951597744	0.998847926	1.042763158	0.870910578	0.142407907	0.07572308
155362	F1158	N	M	0	0.391752577	0.350515464	0.515463918	0.20754717	0.136752137	0.139534884
155362	F1158	N	M	1	0.558375044	0.602466544	0.700733326	0.444444444	0.165378007	0.137537345
155362	F1158	N	M	2	0.493913043	0.356111021	0.617391304	0.298965517	0.271978022	0.131086803
155362	F1158	N	M	4	0.475047081	0.628271128	0.730932203	0.42659805	0.08361204	0.220345345

155362	F1158	N	M	6	0.886363636	0.376986301	0.941142191	0.32054561	0.496229261	0.185560054
155362	F1158	N	M	8	0.494032396	0.46031746	0.635549872	0.28647343	0.212121212	0.16252588
155362	F1158	N	M	24	1.120743034	0.875331565	1.153250774	0.8	0.211528292	0.076712905
155362	F1158	N	M	26	0.481981982	0.503559563	0.453828829	0.445714286	0.330978261	0.05370844
155362	F1158	N	M	28	0.372313843	0.495184591	0.637681159	0.220992908	0.127135601	0.253980404
160013	F1546	N	M	0	0.452830189	0.561403509	0.716981132	0.295081967	0.107142857	0.24137931
160013	F1546	N	M	1	0.517214397	0.450757576	0.634324465	0.314108252	0.233589088	0.117822319
160013	F1546	N	M	2	0.958491871	0.472540212	0.529920443	0.896091811	0.621215043	0.358974359
160013	F1546	N	M	4	0.67570009	0.6019678	0.79765131	0.416149068	0.189006342	0.113636364
160013	F1546	N	M	6	0.506238859	0.451515152	0.581996435	0.366826156	0.181081081	0.086206897
160013	F1546	N	M	8	0.481203008	0.541958042	0.563283208	0.685929187	0.532352941	0.115551116
160013	F1546	N	M	24	0.553511236	0.552560241	0.655898876	0.450420168	0.136392506	0.111111111
160013	F1546	N	M	26	0.502116402	0.445969125	0.529100529	0.428571429	0.068064182	0.084772788
160013	F1546	N	M	28	0.574557316	0.454856863	0.632339236	0.391272903	0.185064935	0.062026862
160013	F1546	N	M	96	0.473389356	0.455486542	0.5821662	0.35625	0.122377622	0.110495908
160021	F1768	N	M	0	0.510714286	0.513177998	0.583333333	0.441904762	0.056891026	0.069452287
160021	F1768	N	M	1	0.495901639	0.524386724	0.717798595	0.301587302	0.183388158	0.225757576
160021	F1768	N	M	2	0.451578384	0.51142664	0.647271268	0.300395257	0.136381248	0.190720328
160021	F1768	N	M	4	1.028248588	0.819587629	1.356610169	0.497524752	0.207459207	0.274344569
160021	F1768	N	M	6	0.622295082	0.472848523	0.744262295	0.371212121	0.286225403	0.119860848
160021	F1768	N	M	8	0.373809524	0.325075834	0.484090909	0.225151705	0.123804124	0.138477801
160021	F1768	N	M	24	0.482435597	0.469621904	0.661202186	0.293247242	0.185132576	0.196535245
160021	F1768	N	M	26	0.526923077	0.419565217	0.607692308	0.455801825	0.613659023	0.082258065
160021	F1768	N	M	28	0.428282828	0.398448773	0.612121212	0.225825472	0.202548853	0.200471698
160021	F1768	N	M	96	0.534255599	0.537064677	0.815546772	0.231973435	0.32826087	0.279411765
160096	F1034	N	F	0	0.404040404	0.306122449	0.505050505	0.217391304	0.196078431	0.112359551
160096	F1034	N	F	1	0.440724206	0.438233264	0.614335317	0.275830044	0.148051948	0.176236045
160096	F1034	N	F	2	0.480796586	0.530804598	0.742769085	0.275114155	0.196236559	0.265454545
160096	F1034	N	F	4	0.699622958	0.474193548	0.916422287	0.276158192	0.42268257	0.225579282
160096	F1034	N	F	6	0.605185185	0.385778275	0.56691358	0.432078313	0.613095238	0.12987013
160096	F1034	N	F	8	1.118369793	0.530763529	1.252622116	0.452121212	0.676268412	0.129444444
160096	F1034	N	F	24	0.513636364	0.373913043	0.627272727	0.239832285	0.26780303	0.12195122
160096	F1034	N	F	26	0.441867656	0.394444444	0.563698207	0.299232737	0.141666667	0.131182796

160096	F1034	N	F	28	0.535362319	0.462043379	0.649855072	0.376417234	0.184129902	0.118872549
160096	F1034	N	F	96	0.578416445	0.576756757	0.82679863	0.333694084	0.25	0.227884615
160121	F1546	N	F	0	0.578313253	0.530120482	0.819277108	0.289156627	0.285714286	0.243902439
160121	F1546	N	F	1	0.500434707	0.419512195	0.683707181	0.419231438	0.694011339	0.182346904
160121	F1546	N	F	2	0.547630294	0.510071475	0.804440246	0.250322997	0.309243697	0.252424242
160121	F1546	N	F	4	0.507936508	0.426229508	0.761904762	0.153846154	0.333333333	0.258064516
160121	F1546	N	F	6	0.551413213	0.521487902	0.814981793	0.254435705	0.304561404	0.270515614
160121	F1546	N	F	8	0.512121212	0.505446623	0.712121212	0.316690856	0.204195804	0.204116638
160121	F1546	N	F	24	0.438991241	0.543066129	0.713832679	0.255952381	0.16827997	0.260619348
160121	F1546	N	F	26	0.427041499	0.415465032	0.592871486	0.230844156	0.170909091	0.189599133
160121	F1546	N	F	28	0.458041958	0.437340153	0.503496503	0.404081633	0.045454545	0.05
160121	F1546	N	F	96	0.476425439	0.488264768	0.63870614	0.323469388	0.138449367	0.159554731
160153	F1768	N	M	0	0.4891445	0.527262045	0.714450036	0.307924985	0.107971413	0.216810683
160153	F1768	N	M	1	0.452991453	0.467440361	0.604395604	0.336507937	0.117094017	0.153485858
160153	F1768	N	M	2	0.557239057	0.522424242	0.730639731	0.325163399	0.228506787	0.172077922
160153	F1768	N	M	4	0.501082251	0.515483694	0.710497835	0.296834458	0.200602905	0.203917051
160153	F1768	N	M	6	0.424242424	0.441176471	0.606060606	0.25	0.142857143	0.176470588
160153	F1768	N	M	8	0.431905864	0.46573296	0.683641975	0.222943723	0.200854701	0.27081448
160153	F1768	N	M	24	0.591694352	0.938235294	0.523920266	0.777777778	0.64893617	0.180392157
160153	F1768	N	M	26	0.493408663	0.281280788	0.562335217	0.116509927	0.483333333	0.202850877
160153	F1768	N	M	28	0.374827586	0.634848139	1.026551724	0.672727273	0.970612245	0.604679803
160153	F1768	N	M	96	0.318498739	0.234151329	0.438065569	0.139495482	0.131180079	0.122342065
160777	F1745	N	M	1	0.853164557	0.841794818	1.008051355	0.671838846	0.247771836	0.174185464
160777	F1745	N	M	2	0.704394693	0.610028349	0.789518032	0.503293808	0.218250631	0.160825476
160777	F1745	N	M	4	0.409920635	0.413651316	0.565942029	0.257526882	0.125	0.183788122
160777	F1745	N	M	6	0.305555556	0.282352941	0.45	0.12987013	0.139534884	0.197183099
160777	F1745	N	M	8	0.588787879	0.505963096	0.694089835	0.378332388	0.185407616	0.181699346
160777	F1745	N	M	24	0.582022472	0.498507463	0.828222997	0.241081081	0.431438127	0.20569566
160777	F1745	N	M	26	0.507072042	0.43452381	0.705426357	0.222417761	0.281705948	0.230443975
160777	F1745	N	M	28	0.48959276	0.499806277	0.683862434	0.316269841	0.141954023	0.203448276
160777	F1745	N	M	96	0.535850496	0.585081585	0.747208931	0.379041249	0.160341151	0.18907563
